# Data Availability Statements of Publications in Journals Indexed in the *Dentistry, Oral Surgery and Medicine Category* of Journal Citation Reports: A Meta-Research Study

**DOI:** 10.1016/j.identj.2026.109543

**Published:** 2026-04-08

**Authors:** Romaissa Rakkani, Niels van der Aa, Clovis Mariano Faggion, Naichuan Su

**Affiliations:** a Department of Oral Public Health, Academic Centre for Dentistry Amsterdam, Amsterdam, The Netherlands; b Faculty of Dentistry, Department of Periodontology and Operative Dentistry, University Hospital Münster, Münster, Germany

**Keywords:** Open science, Research integrity, Data sharing, Open data, Meta-research

## Abstract

To evaluate the frequency and types of data availability statements (DASs) of publications in journals indexed in the *Dentistry, Oral surgery and Medical (DOM)* category of Journal Citation Reports (JCR) database, and to identify risk indicators associated with the presence of DASs in publications. We searched PubMed on October 18, 2024, for publications presenting original research involving human subjects, published in journals indexed in the DOM category of JCR database, after July 1, 2023. Each included publication was assessed for DAS, which was categorised into different types using Springer Nature’s standard DAS framework. The risk indicators regarding author, study and journal levels were extracted. Logistic regression analysis were performed to assess the association between the risk indicators and the presence of DASs. A total of 998 publications were included. Fewer than half (49.7%) of the included publications contained a DAS. The 2 most common DAS types were datasets being available from the corresponding author upon reasonable request with a prevalence of 40.4% (N = 403) and authors directly providing the repository and/or weblink for the datasets with a prevalence of 3.0% (N = 30). The presence of DAS was significantly associated with funding status of publications, journal impact factor and journal requirement on DASs. DASs appear infrequent in publications in journals indexed in the DOM category of JCR database. Funded studies published in journals which require a DAS and journals with a higher impact factor were more likely to contain a DAS than studies which were not funded and published in journals which do not require a DAS with a lower impact factor.

## Introduction

Open science refers to a range of methods, tools, platforms and practices aimed at making scientific research more accessible, transparent, reproducible and reliable.^1^ Open science is multidimensional, including open access, open data, open source and other open scholarship practices.^2^, ^3^, ^4^ Recognition and adoption of open research practices has grown in recent years, driven by evolving new policies that promote public access to academic literature and encourage data sharing.^2^ Open science promotes scientific collaboration, greater visibility and impact of research, improved research quality, and enhanced public engagement.

One of the key challenges that open science aims to address is the reproducibility crisis,^5^ defined as the growing number of published scientific results that other researchers have been unable to reproduce or verify.^6^ It has been estimated that 75% to 90% of the published studies are irreproducible.^7^ The reproducibility crisis in science may undermine the credibility of scientific research, waste resources and lead to flawed decision-making across fields. It also erodes public trust in science and delays the development of new technologies and treatments.

Data sharing is a core component of open science^2^ and one of the key strategies to address reproducibility crisis.^4^ Sharing research data fosters transparency, advances discovery in science and fulfils ethical obligations to study participants.^8^ Data sharing can also save resources and help encourage new study designs, avoid duplication, identify errors and reduce fraud.^9^^,^^10^ Data availability statements (DASs) in publications describe where and under what conditions the data for a research paper can be accessed.^11^ Since 2018, the International Committee of Medical Journal Editors (ICMJE) required that clinical trial reports include a DAS, making it a prerequisite for publication in ICMJE member journals.^11^ Additionally, in 2010s, the European Medicines Agency (EMA) introduced new standards for sharing of clinical trial data that have been submitted for marketing authorisation of medicinal products.^12^

To date, an increasing number of scientific journals require or encourage the inclusion of a DAS in publications. A DAS informs readers about the availability and location of the data,^13^ thereby serving an important first step toward promoting transparency, reproducibility, accountability and responsible data sharing in scientific research. In recent years, data sharing practices in medical research have been increasingly investigated. Prior studies in medical fields have evaluated the frequency of DASs in publications across various medical disciplines.^14^, ^15^, ^16^ In the field of *Dentistry, Oral Surgery and Medicine* (DOM), previous bibliometric studies have examined publication trends, authorship patterns and citation impact, providing insights into research visibility and journal characteristics.^17^, ^18^, ^19^ However, these studies have primarily focused on output and impact rather than transparency practices. To date, no studies have specifically examined the frequency and types of DASs in publications in the field of DOM.

Therefore, the aim of the study is to assess the frequency and types of DASs in publications in scientific journals indexed in the DOM category of Journal Citation Reports (JCR) database and to identify risk indicators associated with the presence of DASs.

## Method and materials

In the present study the PRISMA Statement adapted for meta-research studies by Murad and Wang^20^ was used (Appendix Table 1). This meta-research study was based on published literature and did not involve human or animal subjects, and therefore the ethical approval was not required.

### Eligibility criteria

Publications were included if they met the following inclusion criteria:

1. Publications in journals categorised under DOM in the JCR database;
2. Publications presenting original research involving human subjects, including randomised controlled trials (RCTs), prospective or retrospective cohort studies, cross-sectional studies or case-control studies;
3. Published from July 2023 to October 2024.

According to the 2024 JCR database, a total of 157 journals were listed under the DOM category. The journals were classified into 4 quartiles (Q1, Q2, Q3 and Q4) based on journal impact factor rankings. For each quartile, 5 journals were randomly selected. If a selected journal did not contain any publications meeting the inclusion criteria, it was replaced by another randomly selected journal from the same quartile. As a results, 20 journals were included in the final sample (Appendix Table 2).

### Search strategy

PubMed was used to search the publications in the selected journals on October 18th, 2024. The detailed search strategy is presented in Table 1. However, none of the journals selected from the Q4 quartile of the JCR database were indexed in PubMed, including *Oral Science International, Journal of International Oral Health, Asian Pacific Orthodontic Society (APOS) Trends in Orthodontics, Romanian Journal of Oral Rehabilitation* and *Journal of Dentistry for Children*. Therefore, hand searching was conducted using their official journal websites to identify publications meeting the inclusion criteria.

**Table 1:** Search Strategy in PubMed for the meta-research study.

Database	Search strategy
PubMed	(“Eur J Orthod.”[journal] or “J Oral Biosci.”[journal] or “J Clin Periodontol.”[journal] or “Clin Implant Dent Relat Res.”[journal] or “Int Orthod.”[journal] or “Orthod Craniofac Res.”[journal] or “Gerodontology.”[journal] or “J Craniomaxillofac Surg”[journal] or “Clin Exp Dent Res.”[journal] or “Pediatr Dent.”[journal] or “Oral Radiol.”[journal] or “Cleft Palate Craniofac J.”[journal] or “Clin Adv Periodontics.”[journal] or “Aust Dent J”[journal] or “J Periodontal Res” [journal]) AND (“Randomised Controlled Trial”[publication type] or “observational study”[publication type] or “cohort studies”[Mesh] or “case-control studies”[mesh] or “Cross-Sectional Studies”[mesh]) AND (“2023/07/01”[PDAT]: “2024/10/18”[PDAT]) NOT (“review”[publication type] or “systematic review”[publication type] or “meta-analysis”[publication type]) Extra filter: Humans

### Screening of studies

Two reviewers (RR and NS) screened the titles and abstracts of the publications for eligibility. The first 50 studies were screened in duplicate by the 2 reviewers for calibration. If the agreement exceeded 80%,^21^ one reviewer (RR) screened the remaining studies alone. Any uncertainties during the screening process were resolved through discussion between the 2 reviewers. As the eligibility of all studies could be determined solely based on titles and abstracts, full-text screening was not performed.

### Data extraction

Data extraction was performed for each eligible study after title and abstract screening. During the data extraction phase, full texts of the eligible studies were reviewed to extract information on the type of DAS by using the classification developed by Springer Nature,^22^ which distinguished between the following types of DAS:

1. The authors have indicated in which repository they deposited datasets, and they provided a weblink to the datasets
2. The datasets are available from the corresponding author upon reasonable request
3. The datasets generated are not publicly available due to disclosed reasons but are available from the corresponding author upon reasonable request
4. All data generated or analysed are included in the published study and its supplementary information files
5. Data sharing does not apply to this study because no datasets were generated or analysed during the study
6. The data are available from a third party, and restrictions apply regarding data sharing because data were used under license and therefore are not publicly available. Data are, however, available upon reasonable request and with the permission of the license holder.
7. Data is not possible to share due to some reasons
8. Other

In the present study, DAS types 2 and 3 were combined into 1 single category “The datasets are available from the corresponding author upon reasonable request” due to the similarities.

The characteristics of the included studies at author, publication and journal levels (ie, risk indicators) were also extracted. The list of the risk indicators is presented in Table 2. The first 50 studies were extracted in duplicate by the 2 reviewers (RR and NS) for calibration. If the agreement exceeded 80%,^21^ one review (RR) extracted the data for the remaining studies. Subsequently, a random sample of 20% of the studies were reviewed by NS to check the accuracy of the extracted data. Any discrepancies were resolved through discussion between RR and NS.

**Table 2:** List of the potential risk indicators at author, publication, and journal levels for the reporting of data availability statements (DASs).

Risk indicators	Categories	Definition
Author-level		
Country of first authors	Developing/in transition Developed	The variable is determined based on the affiliations of the first author. If an author had multiple affiliations, the first-listed affiliation was used. The countries were categorised into developing/in transition” or “developed” according to a standard classification system.^23^
Country of last authors	Developing/in transition Developed	The variable is determined based on the affiliations of the last author. If an author had multiple affiliations, the first-listed affiliation was used. The countries were categorised into developing/in transition” or “developed” according to a standard classification system.^23^
Total number of authors	Continuous	
International collaboration	Yes No	The variable is defined as authors affiliated with institutions in at least 2 different countries. If an author had multiple affiliations in different countries, the first-listed affiliation was used when determining international collaborations.^24^
Study-level		
Study type	RCTs Observational studies	
Type of datasets	Original datasets Publicly accessed datasets	Original datasets were generated or collected directly by authors and publicly accessed datasets were obtained from third-party sources.
Funding status	Funded Not funded Not reported	
Journal-level		
Two-year impact factor in 2023	Continuous	Assessed based on the JCR database.
Journal requirements on DASs	Required Not required	Assessed based on the author guidelines published in the official journal websites.

DAS, data availability statement; JCR, journal citation reports; RCT, randomised controlled trial.

### Statistical analysis

Descriptive statistics were used to summarize the frequency and types of DASs and characteristics of the included publications. Mean and standard deviation (SD) were used for continuous variables, while frequencies and proportions were used for categorical variables. To investigate the association between the risk indicators (independent variables) and absence/presence of DASs (dependent variable), logistic regression analyses were performed. First, univariable regression analyses were performed to assess the association between each independent variable and presence of DASs, separately. The variables with a *P* value <.05 were included in the subsequent multivariable regression analysis with backward selection procedure (*P* > .05 for removal). Prior to multivariable regression analysis, multicollinearity among the independent variables was evaluated using the variance inflation factor (VIF). A VIF value >5 was considered indicative of problematic multicollinearity. In this case, the variable with the highest VIF was excluded from the multivariable analysis.^25^ All analyses were conducted using IBM SPSS Statistics (version 29.0).

## Result

The flowchart outlining the selection process is presented in Figure 1. A total of 998 publications were included in the final analysis. The list of the included studies is presented in Appendix Table 3. The distribution of DAS types of the included publications is presented in Table 3. Among the 998 included publications, 502 did not include any DASs while the remaining 496 publications included DASs. The most common type of DAS was that the datasets are available from the corresponding author upon reasonable request, reported in 40.4% of all publications. The second most common type was that the authors have indicated the repository and provided a weblink for the datasets, found in 3.0% of publications.

**Fig:**
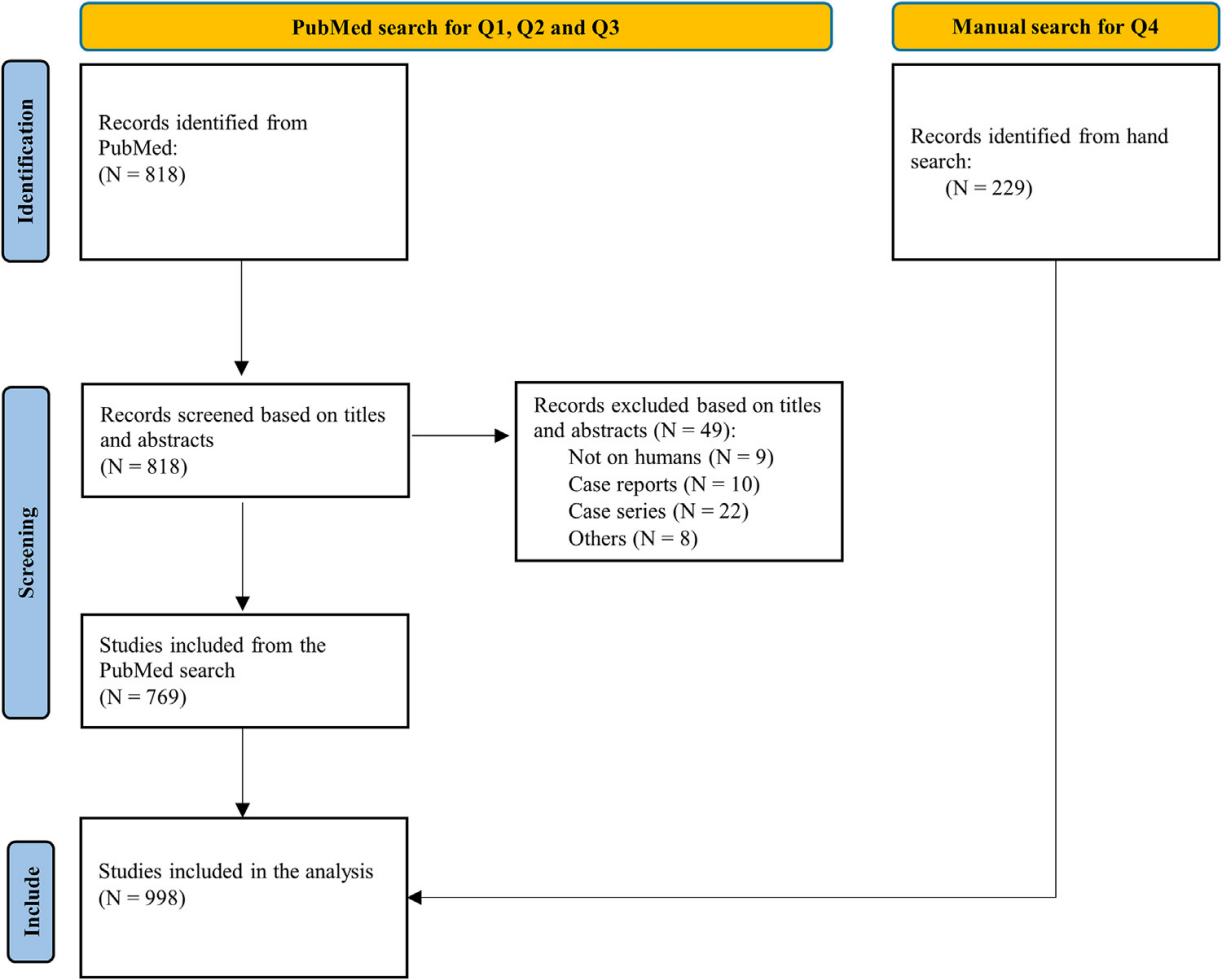
Flowchart of study selection for the meta-research study.

**Table 3:** Distribution of types of data availability statements (DASs) of the included studies.

Types of DASs	Total (N = 998)N (%; 95%CI)	Journal requirements on DASs	
		DASs required (N = 434)N (%; 95%CI)	DASs not required (N = 564)N (%; 95%CI)
No statement	502 (50.3%; 47.2%-53.4%)	11 (2.5%; 1.3%-4.4%)	491 (87.1%; 84.1%-89.6%)
The authors have indicated in which repository they deposited datasets, and they provide a weblink to the datasets	30 (3.0%; 2.1%-4.2%)	27 (6.2%; 4.2%-8.8%)	3 (0.5%; 0.1%-1.4%)
The datasets are available from the corresponding author on reasonable request	403 (40.4%; 37.4%-43.4%)	346 (79.7%; 75.7%-83.3%)	57 (10.1%; 7.8%-12.8%)
All data generated or analysed are included in this published study and its supplementary information files	21 (2.1%; 1.3%-3.1%)	16 (3.7%; 2.2%-5.8%)	5 (0.9%; 0.3%-2.0%)
Data sharing does not apply to this study because no datasets were generated or analysed during the study	7 (0.7%; 0.3%-1.4%)	6 (1.4%; 0.6%-2.9%)	1 (0.2%; 0.0%-0.9%)
The data are available from a third party, and restrictions apply regarding data sharing because data were used under license and therefore are not publicly available. Data are, however, available upon reasonable request and with the permission of the license holder.	11 (1.1%; 0.6%-1.9%)	8 (1.8%; 0.9%-3.5%)	3 (0.5%; 0.1%-1.4%)
Data is not possible to share due to some reasons	20 (2.0%; 1.3%-3.0%)	16 (3.7%; 2.2%-5.8%)	4 (0.7%; 0.2%-1.7%)
Other*	4 (0.4%; 0.1%-1.0%)	4 (0.9%; 0.3%-2.2%)	0 (0.0%; 0.0%-0.5%)

⁎ The DASs of 4 studies were categorised as “other”. One publication stated that the data can only be shared by authorised research institutions and cannot be shared with third parties. Two publications stated that only part of data can be shared upon request, while the other part of the data cannot be shared. The other one publication stated in the DAS section that a DAS was included within the manuscript, but we did not find it. CI, confidence interval.

Among the 998 publications, 434 were published in journals which required DASs, while the other 564 were published in journals which did not require DASs. Of the former group, the majority of publications (97.5%) included a DAS. In contrast, only 12.9% of the publications from journals without a DAS requirement included DASs (Table 3).

Table 4 presents the characteristics of the included publications. Most publications (82.7%) had international collaborations. The first or last authors from more than 60% of publications were from developed countries. The majority of the publications were observational studies (83.5%) while the remaining publications (16.5%) were RCTs. 96.9% of the publications used original datasets collected by the authors while only 3.1% used publicly accessed third-party datasets. The mean journal impact factor was 2.06 ± 1.46. Publications with DASs showed distinct patterns compared with those without DASs across several study and journal characteristics (Table 4). Most publications (97.5%) in journals with requirements on DASs reported DASs, whereas only a small proportion (12.9%) of publications in journals without requirements on DASs reported DASs. Most RCTs (77.6%) reported DASs while over half of the observational studies (55.8%) did not report DASs. Additionally, most publications (77.4%) using publicly accessed datasets reported DASs, whereas over half of the publications (51.2%) using original datasets did not report DASs. Further differences in study and journal characteristics between publications with and without DASs are presented in Table 4.

**Table 4:** Characteristics of the included studies at author, publication, and journal levels and univariable logistic regression analysis for the association between characteristics of the publications and presence of data availability statements (DASs).

	Number of studies (%)/mean (SD)			Univariable logistic regression analysis (absence of DASs as the reference category)		
Independent variables	Overall(N = 998)	Publications with DASs (N = 496)	Publications without DASs (N = 502)	B (SE)	OR (95%CI)	*P*
Number of authors	6.05 (3.20)	6.07 (2.79)	6.02 (3.57)	0.006 (0.020)	1.006 (0.967-1.046)	.773
International collaboration No Yes	825 (82.7%) 173 (17.3%)	373 (45.2%) 123 (71.1%)	452 (54.8%) 50 (28.9%)	Ref. 1.092 (0.182)	2.981 (2.088-4.256)	<.001⁎
Country of first authors Developed Developing or in transition	623 (62.4%) 375 (37.6%)	275 (44.1%) 221 (58.9%)	348 (55.9%) 154 (41.1%)	Ref. 0.597 (0.132)	1.816 (1.401-2.354)	<.001⁎
Country of last authors Developed Developing or in transition	634 (63.5%) 364 (36.5%)	286 (45.1%) 210 (57.7%)	348 (54.9%) 154 (42.3%)	Ref. 0.506 (0.133)	1.659 (1.279-2.152)	<.001⁎
Study type Observational RCT	833 (83.5%) 165 (16.5%)	368 (44.2%) 128 (77.6%)	465 (55.8%) 37 (22.4%)	Ref. 0.369 (0.050)	1.446 (1.311-1.594)	<.001⁎
Type of datasets Original datasets Publicly accessed datasets	967 (96.9%) 31 (3.1%)	472 (48.8%) 24 (77.4%)	495 (51.2%) 7 (22.6%)	Ref. 1.280 (0.434)	3.596 (1.535-8.424)	.003⁎
Funding status Not funded or not reported Funded	611 (61.2%) 387 (38.8%)	207 (33.9%) 289 (74.7%)	404 (66.1%) 98 (25.3%)	Ref. 1.750 (0.145)	5.755 (4.333-7.644)	<.001⁎
Journal impact factor	2.06(1.46)	2.90 (1.57)	1.22(0.61)	1.679 (0.115)	5.359 (4.276-6.717)	<.001⁎
Journal requirement on DASs DASs not required DASs required	564 (56.5%) 434 (43.5%)	73 (12.9%) 423 (97.5%)	491 (87.1%) 11 (2.5%)	Ref. 5.555 (0.330)	258.646 (135.416-494.018)	<.001⁎

B, regression coefficient; CI, confidence interval; OR, odds ratio; RCT, randomised controlled trial; SD, standard deviation; SE, standard error.

⁎ *P* <.05.

Results of the univariable logistic regression analyses are presented in Table 4. Results showed that the presence of DASs was significantly associated with international collaboration (OR: 2.981, 95%CI: 2.088-4.256, *P* < .001), country of first authors (OR: 1.816, 95%CI: 1.401-2.354, *P* < .001), country of last authors (OR: 1.659, 95%CI: 1.279-2.152, *P* < .001), study type (OR: 1.446, 95%CI: 1.311-1.594, *P* < .001), type of datasets (OR: 3.596, 95%CI: 1.535-8.424, *P* = .003), funding status (OR: 5.755, 95%CI: 4.333-7.644, *P* < .001), journal impact factor (OR: 5.359, 95%CI: 4.276-6.717, *P* < .001) and journal requirement on DASs (OR: 258.646, 95%CI: 135.416-494.018, *P* < .001). The VIF value for country of first authors was 7.697, exceeding the threshold of 5 and indicating collinearity. Therefore, this variable was excluded from the subsequent multivariable analysis.

Results of the multivariable logistic regression analysis with backward selection are presented in Table 5. Journal impact factor (OR: 1.653, 95%CI: 1.183-2.310, *P* = .003), funding status (OR: 2.064, 95%CI: 1.257-3.388, *P* = .004) and journal requirement on DASs (OR: 114.953, 95%CI: 56.852-232.431, *P* < .001) remained statistically significantly associated with the presence of DASs. Funded publications had 2.064 times higher odds to contain DASs than those that were unfunded or lacked funding information. Publications in journals with higher impact factors were significantly more likely to contain DASs. Publications in journals with a requirement on DASs had 114.953 times higher odds to contain DASs than those without a requirement on DASs.

**Table 5:** Multivariable logistic regression analysis for the association between characteristics of the publications and presence of data availability statements (DASs).

Independent variables	Full model			Model with backward selection		
	B (SE)	OR (95%CI)	*P*	B (SE)	OR (95%CI)	*P*
International collaboration No Yes	Ref. 0.454 (0.336)	1.575 (0.815-3.043)	.176			
Country of last authors Developed Developing or in transition	Ref. -0.140 (0.259)	0.869 (0.524-1.443)	.588			
Study type Observational RCT	Ref. 0.096 (0.099)	1.101 (0.907-1.336)	.332			
Type of datasets Original datasets Publicly accessed datasets	Ref. 0.246 (0.867)	1.278 (0.234-6.994)	.777			
Funding status Not funded or not reported Funded	Ref. 0.761 (0.258)	2.141 (1.290-3.553)	.003⁎	Ref. 0.724 (0.253)	2.064 (1.257-3.388)	.004⁎
Journal impact factor	0.475 (0.171)	1.608 (1.150-2.248)	.005⁎	0.503 (0.171)	1.653 (1.183-2.310)	.003⁎
Journal requirement on DASs DASs not required DASs required	Ref. 4.697 (0.362)	109.636 (53.950-222.799)	<.001⁎	Ref. 4.745 (0.359)	114.953 (56.852-232.431)	<.001⁎

B, regression coefficient; CI, confidence interval; OR, odds ratio; RCT, randomised controlled trial; SE, standard error.

⁎ *P* <.05.

## Discussion

In the present study, more than half of the publications published in the journals indexed in the DOM category of JCR database lacked a DAS. Among those that included a DAS, the most frequent type was that datasets are available upon reasonable request from corresponding authors. Funded studies published in journals which requires a DAS and journals with a higher impact factor were more likely to include a DAS than studies which were not funded or with unknown funding information and published in journals which do not require a DAS with a lower impact factor.

Several previous studies have assessed the frequency of DASs in scientific publications in general medicine or some special medical fields. For example, Danchev et al.^14^ assessed 487 clinical trials published in 3 leading medical journals, including JAMA, Lancet and New England Journal of Medicine after implementation of ICMJE. They found that 68.6% of the included publications (N = 334) included DASs and most of the publications with DASs (42.8%) reported that datasets were available upon reasonable request from authors. Only 2 publications made the datasets publicly available for others. Gabelica et al.^15^ assessed 3556 publications from 333 open-access journals published during January 2019 by BioMed Central. They reported that 96.1% of the publications (N = 3416) included DASs and the most frequent type (42%) was that datasets are available upon reasonable request. Only 10.8% of the publications provided a direct weblink to a data repository. Schueller et al.^16^ assessed 318 RCTs published in orthodontic journals from 2019 to 2023. They found that only 22.1% of the RCTs included DASs. Among these, the most frequent type is that authors had no intension to share, while the second most frequent type is that the datasets can be shared upon request. These findings suggest that the prevalence of DASs varies significantly across medical disciplines. This variation may be partly explained by differences in disciplinary norms and cultural attitudes toward data sharing across medical sub-fields. Archer et al.^26^ examined 2941 publications in cardiology, general medicine, emergency medicine and orthopaedic surgery journals and reported substantial heterogeneity in the prevalence of DASs across disciplines. The prevalence of DASs was highest in general medicine (96%), whereas it was lowest in emergency medicine (12%).^26^ However, the authors also emphasised that journal policy enforcement, rather than discipline-specific norms alone, plays a major role in driving the adoption of DASs.^26^ However, when DASs are reported, the most or second most common type consistently indicates that data are available upon reasonable request from the authors.

In the present study, over half of the publications did not include DASs, which may be explained by several reasons. First, researchers may lack awareness of the importance and benefits of data sharing, which may stem from insufficient training in research data management. For example, Milewska et al.^27^ assessed the status of knowledge about research data management and the attitudes towards data sharing among 603 Polish medical researchers based on a survey. They found that 55.7% of the researchers only shared data with their own research teams, while only 9.8% had shared data on an open-access basis. Most of them were unaware of the processes required for safe data preparation for sharing and the benefits for data sharing.^27^ The survey^27^ presented several barriers to data sharing: 68.8% of the researchers did not know how to protect themselves against unfair use of the data; 66.8% of them were uncertain about how legal and ethical standards; and 45.1% were afraid someone else would benefit from the results of their work. The findings highlight a critical need for targeted training and support to improve data sharing literacy among medical researchers. Second, some journals or publishers do not require authors to include DASs in the publications or lack clear policies on DASs. This may discourage researchers from proactively including a DAS in the publications. Third, almost all the current reporting guidelines do not explicitly recommend the inclusion of a DASs in research reporting, which may contribute to its underreporting. Fourth, data localisation and privacy laws vary significantly across countries. National regulations on the storage, transfer and sharing of personal data may impose legal and ethical constraints that limit researchers` ability to share data across borders.^28^

In the present study, funding status, journal impact factor and journal requirements on DASs were significantly associated with the presence of DASs. To date, an increasing number of funding agencies require or encourage researchers to share the data. For example, the US National Institute of Health (NIH) implemented a Data Management and Sharing Policy in January 2023, mandating that researchers plan for and share scientific data from the NIH-funded research.^29^ Similarly, the Dutch ZonMw (Dutch Organisation for Health Research and Development) requires that data from funded projects be as open as possible.^30^ This may explain why funded publications were significantly more likely to have DASs than the publications which were unfunded or with unknown funding information. Similar to our study, Schueller et al.^16^ reported that funded publications in orthodontics had 2.76 to 3.13 times higher odds to report on data sharing compared to nonfunded publications. Additionally, the present study found that publications in journals with higher impact factors were more likely to have DASs than those with lower impact factors. This may be because high-impact journals, often associated with major publishers such as Wiley, Elsevier and Springer, are more likely to adopt and enforce strict data sharing policies. For example, Wiley explicitly stated that all research- and synthesis-based articles must include a DAS, whether or not the data used in the article is shared.^31^ In contrast, the less well-known publishers, which are more commonly associated with lower-impact journals, are less likely to have explicit or enforced data sharing policies. Resnik et al.^32^ found that journals with higher impact factors were more likely to have formal data sharing policies; encourage or require deposit of data in public repositories (eg, for proteomic, genetic and genomic data); incorporate shared data into peer review process; and refer to reproducibility as a rationale for promoting data sharing, based on 447 journals across several scientific disciplines. Furthermore, the present study also found that journal requirements on DASs had a very strong influence on the reporting of DASs of the publications. The large odds ratio (ie, 114.953) observed for journal requirements on DASs should be interpreted with caution. This strong association is more likely to reflect structural enforcement of journal policies rather than author-level behaviour or voluntary engagement in data sharing. When journals mandate the inclusion of a DAS as part of the submission or publication process, authors` compliance becomes procedural, which may result in a substantially increased probability of DAS reporting by design. Therefore, the magnitude of this effect should not be directly interpreted as evidence of a corresponding change in authors` data-sharing practices, but rather as an indication of the effectiveness of journal policies in ensuring the presence of DASs.

It should be noted that only 1 electronic database (ie, PubMed) was used for article retrieval in the study, but this approach was unlikely to result in missing eligible publications. This is because the journals included in the study were preselected based on the JCR database prior to the article searching in PubMed. PubMed served only as a tool for retrieving articles from these preselected journals. All journals selected from Q1 to Q3 of the JCR database were indexed in PubMed. For Q4 journals not indexed in PubMed, a manual search of official journal websites was conducted to ensure the comprehensive coverage. Therefore, all eligible publications from the preselected journals were included.

The present study has several limitations. First, although the most common type of DASs was datasets being available from the corresponding author upon reasonable request, this type of DASs only represents a nominal level of transparency and does not necessarily ensure the meaningful data accessibility. We did not assess whether authors actually complied with their published DASs. The mere presence of a DAS in a publication does not guarantee that the underlying data are genuinely shared. For instance, Gabelica et al.^15^ found that only 123 out of 1,792 authors (6.8%) from 333 open-access journals in BioMed Central who indicated a willingness to share data in their DASs actually provided the requested data. This suggests that reliance on DAS frequency alone may not accurately reflect real-world data sharing practices in medical field. Consequently, the DAS may offer limited support for reproducibility and independent verification of research findings. The high prevalence of such type of DAS observed in the study may overestimate the true level of open data practices within DOM field. Nevertheless, the inclusion of DASs in publications represents an important initial step toward encouraging transparency and promoting data sharing among researchers. Second, although journals were randomly selected from each quartile of the JCR database, some subfields of DOM, such as dental public health, endodontics and oral and maxillofacial surgery, may be underrepresented because no journals from these areas were included in the sample. While we do not anticipate substantial differences in DAS frequency across subfields in DOM, this sampling limitation may affect the generalisability of our findings. Third, the present study was limited to journals indexed in the DOM category of the JCR database, and therefore may not represent the broader fields of DOM as a whole. Publications in non-JCR-indexed journals in the same field may differ in the reporting of DAS. Therefore, the findings of the study should be interpreted within the context of this specific journal category and generalised with caution beyond it.

Future research is recommended to move beyond simply documenting the presence of DASs and instead assess actual data sharing practices among researchers in DOM. In addition, qualitative studies are warranted to explore researchers’ attitudes, perceived barriers, and contextual factors influencing data sharing behaviours within dental fields. Publishers and journals are encouraged to adopt more explicit and stringent requirements for DASs and to follow up on compliance. Professional associations and funding agencies may further promote data transparency by developing field-specific standards and requiring DASs as part of publication and funding policies. Such coordinated efforts could substantially improve the adoption of DASs in dental and oral health research.

## Conclusion

DASs appear infrequent in publications in journals in DOM. Funded studies published in journals which require a DAS and journals with a higher impact factor were more likely to contain a DAS than studies which were not funded and published in journals which do not require a DAS with a lower impact factor. To enhance data transparency, journals, professional associations, and funding agencies are recommended to implement clear and enforceable DAS policies, provide guidance on data-sharing practices, and monitor compliance.

## Author contributions

*Concept and design:* All authors.

*Acquisition, analysis, or interpretation of data:* Romaissa Rakkani, Naichuan Su.

*Drafting of the manuscript:* Romaissa Rakkani, Naichuan Su.

*Critical revision of the manuscript for important intellectual content:* All authors.

*Statistical analysis:* Romaissa Rakkani, Naichuan Su.

*Administrative, technical, or material support:* Romaissa Rakkani, Naichuan Su.

*Supervision:* Niels van der Aa, Naichuan Su.

*Methodology:* Romaissa Rakkani, Niels van der Aa, Naichuan Su.

## Funding

This research did not receive any specific grant from funding agencies in the public, commercial, or not-for-profit sectors.

## Data availability

Data will be made available upon request.

## Declaration of competing interest

The authors declare that they have no known competing financial interests or personal relationships that could have appeared to influence the work reported in this paper.

## Appendix Supplementary materials

Image, application 1

## Footnotes

Supplementary material associated with this article can be found in the online version at doi:10.1016/j.identj.2026.109543.
