# Supplementary material for: Data Availability Statements of Publications in Journals Indexed in the Dentistry, Oral Surgery and Medicine Category of Journal Citation Reports: A Meta-Research Study
Source: Int Dent J. 2026 Apr 8;76(3):109543. doi: 10.1016/j.identj.2026.109543 (PMC13091431; doi:10.1016/j.identj.2026.109543)
Supplement: Supplementary file 1 [file mmc1.docx]

**Appendix Table 1 PRISMA checklist**

| Section/topic | Proposed item to be used in methodology research | Page |
| --- | --- | --- |
| Title |  |  |
| Title | Identify the report as a meta-epidemiologic study. | 1-2 |
| Abstract |  |  |
| Structured summary | Provide a structured summary that includes the background of the topic, goal of the study, data sources, method of data selection, appraisal and synthesis methods, results, limitations, conclusions and implications of key findings. | 2 |
| Introduction |  |  |
| Rationale | Describe the rationale for the meta-epidemiological study in the context of what is already known. | 4 |
| Objectives | Provide an explicit statement of the goal of the meta-epidemiological study and the hypothesis being empirically tested. | 4 |
| Methods |  |  |
| Protocol | Indicate if a protocol exists, if and where it can be accessed (eg, Web address). Registration of a protocol is not mandatory. | NA |
| Eligibility criteria | Specify study characteristics used as criteria for eligibility with a rationale. | 5 |
| Information sources | Describe all information sources (eg, databases with dates of coverage, contact with experts to identify additional studies, Internet searches) and search date. | 5 |
| Search | Present full electronic search strategy for at least one database, including any limits used, such that it could be repeated. Search is commonly not driven by a clinical question. | Table 1 |
| Study selection | Describe the process for selecting studies for inclusion (ie, how many reviewers selected studies, reviewing in duplicate or by single individuals). | 5 |
| Data collection process | Describe method of data extraction from reports (eg, piloted forms, independently, in duplicate) and any processes used for manipulating data or obtaining and confirming data from investigators. | 6 |
| Data items | List and define all variables for which data were sought and any assumptions and imputations made. | 6 |
| Risk of bias in individual studies | If risk of bias assessment of individual studies was relevant to the analysis, describe the items used and how this information is to be used during data synthesis. | NA |
| Summary measures | State the principal summary measures (eg, ratio of risk ratios, difference in means) and explain its meaning and direction to readers. | 6-7 |
| Synthesis of results | Describe the statistical or descriptive methods of synthesis including measures of consistency if relevant. If applicable, describe the development of statistical or simulation modelling based on theoretical background. Describe and justify assumptions and computational approximations. Describe methods of additional analyses (eg, sensitivity or subgroup analyses, meta-regression), if done, indicating which were prespecified. | 6-7 |
| Results |  |  |
| Study selection | Give numbers of studies assessed for eligibility and included in the study, with reasons for exclusions at each stage, ideally with a flow diagram. Present a measure of inter-reviewer agreement (eg, kappa statistic). | 7 and figure 1 |
| Study characteristics | For each study, present characteristics for which data were extracted and provide the citations. Clinical characteristics may not always be relevant. | 7 and Table 3 |
| Risk of bias within studies | If risk of bias assessment of individual studies was used in the meta-epidemiological analysis, report risk of bias indicators of each study to allow replication of findings. | NA |
| Results of individual studies | Present data elements used in the meta-epidemiological analysis from each study (results of clinical outcomes may not be relevant). | NA |
| Synthesis of results | Present results of statistical analysis done, including measures of precision and measures of consistency. Present validity of assumptions and fit of statistical or simulation modelling, if applicable. | 7-8 |
| Additional analysis | Give results of additional analyses, if done (eg, sensitivity or subgroup analyses, meta-regression). | NA |
| Discussion |  |  |
| Summary of evidence | Summarise the main findings and compare them with existing knowledge about the topic. The quality of evidence may not be relevant; however, investigators should describe their certainty in the results to readers. | 8 |
| Limitations | Discuss limitations at research methodology level (eg, likelihood of reporting or publication bias). | 9-10 |
| Conclusions | Provide general interpretation of the results and implications for future research. Provide any plausible impact on clinical practice. | 10 |
| Funding |  |  |
| Funding | Describe sources of funding for the methodology research and role of funders. | 10 |

**Appendix Table 2 List of the included journals**

| - Eur J Orthod - J Oral Biosci - J Clin Periodontol - Clin Implant Dent Relat Res - Int Orthod - Orthod Craniofac Res - Gerodontology - J Craniomaxillofac Surg - Clin Exp Dent Res - Pediatr Dent - Oral Radiol - Cleft Palate Craniofac J - Clin Adv Periodontics - Aust Dent J - J Periodontal Res - Oral Science International - Journal of International Oral Health - Asian Pacific Orthodontic Society (APOS) Trends in Orthodontics - Romanian Journal of Oral Rehabilitation - Journal of Dentistry for Children |
| --- |

**Appendix Table 3 List of the included studies**

| **ID** | **corresponding author name** | **study title** | **journal name** |
| --- | --- | --- | --- |
| #1 | Margarita Iniesta | Subgingival microbiome in periodontal health, gingivitis and different stages of periodontitis | Journal of clinical periodontology |
| #2 | Danfeng Shen | Visceral adipose tissue increases the risk of periodontal disease: Results from the 2011–2014 National Health and Nutrition Examination Survey and Mendelian randomization analysis | Journal of clinical periodontology |
| #3 | Eduardo Montero | Cross-sectional association between severe periodontitis and diabetes mellitus: A nation-wide cohort study | Journal of Clinical Periodontology |
| #4 | Carolina Modin | Periodontitis in young individuals: Important factors for disease progression | Journal of Clinical Periodontology |
| #5 | Ema Zubovic | Variability and Excess in Opioid Prescribing Patterns After Cleft and Craniosynostosis Repairs | The Cleft Palate Craniofacial Journal |
| #6 | Weijing Wang and Dongfeng Zhang | Associations between oral health and depression and anxiety: A cross-sectional and prospective cohort study from the UK Biobank | Journal of Clinical Periodontology |
| #7 | Luigi Nibali | Tooth loss during supportive periodontal care: A prospective study | Journal of Clinical Periodontology |
| #8 | Lixin Qiu | Immediately or delayed sinus augmentation after pseudocyst removal: A randomized trial | Clinical Implant Dentistry and Related Research |
| #9 | Lewis Winning | Subgingival microbial diversity and respiratory decline: A cross-sectional study | Journal of Clinical Periodontology |
| #10 | Amy E. Millen | Association between dietary patterns and periodontal disease: The OsteoPerio cohort study | Journal of Clinical Periodontology |
| #11 | Ying Shan & Hongyu Yang | Association between periodontal diseases and chronic obstructive pulmonary disease: Evidence from sequential cross-sectional and prospective cohort studies based on UK Biobank | Journal of Clinical Periodontology |
| #13 | Yasser Alsayed Tolibah | Prevalence and risk factors of peri‐implant diseases at patient‐level: A cross‐sectional study in Syria | Clinical and Experimental Dental Research |
| #14 | Brianne B. Roby | Prenatal Consultation Outcomes for Infants With Cleft Lip With and Without Cleft Palate | The Cleft Palate Craniofacial Journal |
| #15 | Paul L. Fidel, Jr. | Current oral hygiene and recreational behavioral trends in HIV disease | Clinical and Experimental Dental Research |
| #16 | Sarah Glass | Head and neck exam practices of dental professionals | Clinical and Experimental Dental Research |
| #17 | Silvia Amélia Scudeler Vedovello | Should midline diastema in mixed dentition be an aesthetic concern? | Orthodontics and Craniofacial Research |
| #18 | Alaa A. Alkhateeb | Assessment of Oral Hygiene and Gingivitis in Adolescents With and Without Cystic Fibrosis | Pediatric Dentistry |
| #19 | Roberto Carlos Castrejón-Pérez | Frailty index and ten oral conditions in the Coyoacan cohort study: A cross-sectional analysis | Gerodontology |
| #20 | Anthony S. de Buys Roessingh | Unilateral Cleft lip and Palate: Long-Term Results of the Malek Technique | The Cleft Palate Craniofacial Journal |
| #21 | Susan Thibeault | Are Children with Cleft Palate at Increased Risk for Laryngeal Pathology? | The Cleft Palate Craniofacial Journal |
| #22 | Laura A. Jacox | Evaluating video-based lectures on YouTube for dental education | Orthodontics and Craniofacial Research |
| #23 | Francesco Grande | Rehabilitation with implant-supported overdentures in preteens patients with ectodermal dysplasia: A cohort study | Clinical Implant Dentistry and Related Research |
| #24 | Demet Yolcu | Post-oncological full-thickness eyelid defect reconstruction: An aesthetical overview | Journal of cranio-maxillo-facial surgery |
| #25 | Darin Patmon | Racial Disparities in the Timing of Alveolar Bone Grafting | The Cleft Palate Craniofacial Journal |
| #26 | Zokaris Nikolaos | Instrumental Occlusal Analysis in Migraine Patients: A Quantitative Cross Sectional Study | Clinical and Experimental Dental Research |
| #27 | Jun Wang | Enlarged occipital spur and craniofacial morphology: a cephalometric analysis | Oral Radiology |
| #28 | Ausra Ramanauskaite | Progressive bone loss and bleeding on probing: A cohort study | Clinical Implant Dentistry and Related Research |
| #29 | Jeffrey P. Simons, | Prevalence and Management of Laryngomalacia in Patients With Pierre Robin Sequence | The Cleft Palate Craniofacial Journal |
| #30 | Jingtao Li | A retrospective cephalometric study on the craniofacial morphology of adult patients with unoperated submucous cleft palate | Journal of cranio-maxillo-facial surgery |
| #31 | Mahmoud M Bakr | Microbial Analysis of Obturators During Maxillofacial Prosthodontic Treatment Over an 8-Year Period | The Cleft Palate Craniofacial Journal |
| #32 | Charlotta Gustafsson | Long-term Follow-up of Bilateral Cleft Lip and Palate: Incidence of Speech-Correcting Surgeries and Fistula Formation | The Cleft Palate Craniofacial Journal |
| #33 | Nicolas S. Poupore | Cleft Lip and/or Palate Repair in Children With Hypopituitarism: Analysis of the Kids’ Inpatient Database | The Cleft Palate Craniofacial Journal |
| #34 | Jessye Melgarejo do Amaral Giordani | Social capital and dental service use in older Brazilians | Gerodontology |
| #35 | Golnoush Farzinnia | Three-Dimensional Assessment of Eustachian Tube in Patients With Cleft Palate Versus Controls: A CBCT Study | The Cleft Palate Craniofacial Journal |
| #37 | Jin Magara | Evaluating the effect of management on patients with oral hypofunction: A longitudinal study | Gerodontology |
| #38 | Bing Shi | Cephalometric Soft Tissue Morphology of Adults With Unoperated Submucous Cleft Palate | The Cleft Palate Craniofacial Journal |
| #39 | Antonio Barone | Effect of alveolar ridge preservation on peri-implant mucositis and peri-implantitis prevalence: A multicenter, cross-sectional study | Clinical Implant Dentistry and Related Research |
| #40 | Tiffanie-Marie Borg | Submucous Cleft Palate (SMCP): Indications and Outcomes of Radical Muscle Dissection Palatoplasty in Children Under 4 Years of Age | The Cleft Palate Craniofacial Journal |
| #41 | Elsa M. Ronde | Evaluating International Diagnostic, Screening, and Monitoring Practices for Craniofacial Microsomia and Microtia: A Survey Study | The Cleft Palate Craniofacial Journal |
| #43 | Flavio de Mendonca Copello | 3D analysis of maxillomandibular morphology in hyperdivergent and hypodivergent individuals: A cross-sectional study | Orthodontics and Craniofacial Research |
| #44 | Simone Soares | Arch Symmetry in Patients Without and With Cleft Lip and Palate After Orthodontic/Rehabilitative Treatment—A Stereophotogrammetry Study | The Cleft Palate Craniofacial Journal |
| #45 | Andreas Naros | Autologous Tooth Transplantation in Craniofacial Malformations | The Cleft Palate Craniofacial Journal |
| #46 | Yaping Pan, | Associations of dietary antioxidant intake with periodontal health among US adults: An exploratory mediation analysis via mitochondrial function | Journal of clinical periodontology |
| #47 | Samuel Reeves | Gender representation amongst orthodontic editorial boards: trends over time | European Journal of Orthodontics |
| #49 | Dr. Jesse A. Goldstein, | 3D Photography to Quantify the Severity of Metopic Craniosynostosis | The Cleft Palate Craniofacial Journal |
| #50 | Mohamad Talal Radwan | Artificial intelligence-based algorithm for cervical vertebrae maturation stage assessment | Orthodontics and Craniofacial Research |
| #51 | Tae-Suk Oh, | Comparison of Cleft Lip Nasal Deformities Between Lesser-Form and Incomplete Cleft Lips: Implication for Primary Rhinoplasty | The Cleft Palate Craniofacial Journal |
| #52 | Anne Nordrehaug Åstrøm | The dentist-patient relationship and oral health–related quality of life among older adults: A cohort study | Gerodontology |
| #53 | Michael Bezuhly, | Comparison of two Specialized Cleft Palate Feeders | The Cleft Palate Craniofacial Journal |
| #54 | Noriko Takeuchi, | Effect of oral health status and oral function on malnutrition in community-dwelling older adult dental patients: A two-year prospective cohort study | Gerodontology |
| #55 | Hakan Turkkahraman | Can we predict orthodontic extraction patterns by using machine learning? | Orthodontics and Craniofacial Research |
| #57 | Jesse A. Goldstein | Review of Diet Protocols Following Orthognathic Surgery and Analysis of Postoperative Weight Loss | The Cleft Palate Craniofacial Journal |
| #58 | Ho-Jui Tung | Incident edentulism and number of comorbidities among middle-aged and older Americans | Gerodontology |
| #59 | Silvia R. A. Reis, | Root Curvature in Non-Syndromic Oral Clefts: A Case-Control Study in a Brazilian Population | The Cleft Palate Craniofacial Journal |
| #60 | Marco M. Herz | Periodontal pockets: Predictors for site-related worsening after non-surgical therapy—A long-term retrospective cohort study | Journal of clinical periodontology |
| #61 | Malou S. Willems | Care-resistant behaviour during oral examination in Dutch nursing home residents with dementia | Gerodontology |
| #62 | Roberto L. Flores | Implementation of an Ambulatory Cleft Lip Repair Protocol: Surgical Outcomes | The Cleft Palate Craniofacial Journal |
| #63 | Jasmina Primozic | Skeletal and dental effects of serial extractions performed with or without maxillary expansion—A retrospective controlled study | Orthodontics and Craniofacial Research |
| #64 | Barbara Janssens | Frailty as a determinant of dental attendance among community-dwelling older adults | Gerodontology |
| #65 | Xianping Tang | Investigating oral health-related quality of life in patients with ischaemic stroke in China | Gerodontology |
| #66 | A.J. Agopian, | Epidemiology of Nonsyndromic, Orofacial Clefts in Texas: Differences by Cleft Type and Presence of Additional Defects | The Cleft Palate Craniofacial Journal |
| #67 | Mohammed Farid | The Incidence and Outcomes for Children with Cleft Palate and/or Lip and CHARGE Syndrome | The Cleft Palate Craniofacial Journal |
| #68 | Jarno Hakkers | Do systemic amoxicillin and metronidazole during the non-surgical peri-implantitis treatment phase prevent the need for future surgical treatment? A retrospective long-term cohort study | Journal of clinical periodontology |
| #69 | Leandro Machado Oliveira | Binge drinking and oral health-related quality of life in older adults: Socioeconomic position matters | Gerodontology |
| #70 | Anniina M. Launonen | A longitudinal study of facial asymmetry in a normal birth cohort up to 6 years of age and the predisposing factors | European Journal of Orthodontics |
| #71 | Amirtaher Mirmortazavi & Reza Shakiba | Mallampati score in patients with temporomandibular joint disorders: A pilot case‐control study | Clinical and Experimental Dental Research |
| #72 | Letícia Pereira Martins | Early Introduction of Total Sugar Foods and Early Childhood Caries | Pediatric Dentistry |
| #73 | Melih Motro | Effects of counterclockwise bimaxillary advancement surgery and relapse on upper airway | Orthodontics and Craniofacial Research |
| #74 | Antonio Vicente | Quantitative assessment of cleft volume and evaluation of cleft’s impact on adjacent anatomical structures using CBCT imaging | Oral Radiology |
| #75 | Xudong Wang | Timing of oral and maxillofacial surgery in infected COVID-19 subjects: A retrospective cohort study | Journal of cranio-maxillo-facial surgery |
| #76 | Brian Kinard | Current Orthognathic Surgery Practice Patterns Among Academic OMS | The Cleft Palate Craniofacial Journal |
| #77 | Jorge Gamonal | Functional dentition and well-being among Chilean 80-year-olds | Gerodontology |
| #78 | Stephen L-K. Yen | Impact of protraction or orthognathic surgery for class III malocclusion on longitudinal quality of life in patients with cleft lip and palate | Orthodontics and Craniofacial Research |
| #79 | Taner Ozturk | Mandibular Radiomorphometric Characteristics of Individuals with Bilateral or Unilateral Cleft Lip and Palate | The Cleft Palate Craniofacial Journal |
| #80 | Yen‐Chin Chen | The relationship between oral frailty and oral dysbiosis among hospitalized patients aged older than 50 years | Clinical and Experimental Dental Research |
| #81 | Alexandre Rezende Vieira | Molar-incisor hypomineralization in a cohort of individuals born with cleft lip and palate | Orthodontics and Craniofacial Research |
| #82 | Katherine Hare | Immediately loaded full arch implant rehabilitation and oral health-related quality of life: A retrospective cohort study from primary dental care | Clinical Implant Dentistry and Related Research |
| #84 | Lili Ács | Maternal factors in the origin of cleft lip/cleft palate: A population-based case–control study | Orthodontics and Craniofacial Research |
| #85 | Mi Kyung Kim | Association between inflammatory potential of diet and periodontitis disease risks: Results from a Korean population-based cohort study | Journal of clinical periodontology |
| #86 | Rami S. Kantar | The First Hybrid International Educational Comprehensive Cleft Care Workshop | The Cleft Palate Craniofacial Journal |
| #87 | Rami R. Hallac | Three-dimensional Anthropometric Analysis of Racial and Ethnic Differences in Unilateral and Bilateral Cleft Nasal Deformity | The Cleft Palate Craniofacial Journal |
| #88 | Pekka Niemi | Costs and duration of orthodontic-surgical treatment with mandibular advancement surgery | European Journal of Orthodontics |
| #89 | Chris Wyatt | Tooth loss and dementia amongst older adults residing in long-term care facilities in Vancouver: A case-control study | Gerodontology |
| #91 | Jesse A Taylor | Narcotic Utilization After Cleft Lip Repair: Does Local Anesthetic Choice Matter? | The Cleft Palate Craniofacial Journal |
| #92 | Katherine France | Oral healthcare providers play a vital role in vaccination efforts: Patient perspectives | Clinical and Experimental Dental Research |
| #93 | Elin Hadler‐Olsen | Are dental visiting patterns and oral pain associated with dental disease among Norwegian adults? A cross‐sectional study based on the Tromsø study | Clinical and Experimental Dental Research |
| #94 | Geetika Sobti | Co-Occurrence of Taurodontism in Nonsyndromic Cleft Lip and Palate Patients in Subset of Indian Population: A Case–Control Study Using CBCT | The Cleft Palate Craniofacial Journal |
| #95 | Hanyao Huang | Posttraumatic Growth and its Correlates Among Parents of Children With Cleft Lip and/or Palate | The Cleft Palate Craniofacial Journal |
| #96 | Thais Maria Freire Fernandes | Dental arch changes comparison between Expander with Differential Opening, Hyrax-type and Haas-type expanders: A prospective clinical study | Orthodontics and Craniofacial Research |
| #97 | Jan Oliver Voss | Diagnostic pitfalls in pediatric orbital entrapment fractures | Journal of cranio-maxillo-facial surgery |
| #98 | Osama Mohammed Raouf Askar | Rehabilitation of distal extension maxillary ridges with fixed and removable implant-supported prostheses: Preliminary 12-month randomized clinical trial | Clinical Implant Dentistry and Related Research |
| #99 | Aitziber Fernández-Jiménez | Presence and implication of Candida spp. in patients with peri-implantitis enrolled in a supportive peri-implant therapy program of the Basque Country (Spain). A case–control study | Clinical Implant Dentistry and Related Research |
| #100 | Namiaki Takahara | Comparison of magnetic resonance imaging findings in patients with intermittent closed lock and acute closed lock of the temporomandibular joint: a cross‑sectional retrospective study | Oral Radiology |
| #101 | Kug Jin Jeon | CT and MR imaging findings of head and neck chondrosarcoma | Oral Radiology |
| #102 | Ratchawan Tansalarak | Three-dimensional Changes of Maxillary Alveolar Morphology After Using Modified Nasoalveolar Molding in Patients with Complete Unilateral Cleft lip and Palate | The Cleft Palate Craniofacial Journal |
| #103 | Patrick M. Briley | Impact of Insurance Status on Initiation of Breast Milk Feeding Among Infants With CL± P | The Cleft Palate Craniofacial Journal |
| #104 | Zohreh Dalirsani | Knowledge and attitude of pregnant women in Urmia, Iran, about oral health care during pregnancy | Clinical and Experimental Dental Research |
| #105 | María Clara González-Carrera | Parents’ Perception of Barriers to the Comprehensive Management of Children With Cleft Lip and Palate in Bogota, Colombia | The Cleft Palate Craniofacial Journal |
| #106 | Paolo Scolozzi | Does the type of craniomaxillofacial fracture (CMF) differ between patients with intracranial hemorrhage (ICH) and those with blunt cerebrovascular injury (BCVI)? A retrospective study | Journal of cranio-maxillo-facial surgery |
| #107 | Ying Wang & Guoli Yang | Survival analysis of implants placed simultaneously with lateral sinus floor elevation in severely atrophic maxilla: A 3- to 12-year retrospective cohort study | Clinical Implant Dentistry and Related Research |
| #108 | Hamoun Sabri | Periodontal status following orthodontic mini‐screw insertion: A prospective clinical split‐mouth study | Clinical and Experimental Dental Research |
| #109 | Chad A. Purnell | Differences in Practice in Alveolar Bone Grafting Among American Cleft Palate- Craniofacial Association Members | The Cleft Palate Craniofacial Journal |
| #110 | Ritu Duggal | Comparative evaluation of nasolabial appearance of unilateral cleft lip and palate patients by professional, patient and layperson using 2 aesthetic scoring systems: A cross sectional study | Orthodontics and Craniofacial Research |
| #111 | Fabian Fenske | Evaluating the efficacy of single-shot versus prolonged antibiotic prophylaxis in alveolar cleft osteoplasty – A retrospective cohort study | Journal of cranio-maxillo-facial surgery |
| #112 | Dr. Adekunle Adegbayi | Wound Healing Following Palatoplasty Using Either Honey or Warm Saline Mouth Bath for Postoperative Wound Care: A Randomized-Controlled Study | The Cleft Palate Craniofacial Journal |
| #113 | H´elios Bertin | Stability of Le Fort 1 osteotomy in patients with bilateral cleft lip and palate: A retrospective study of 71 patients | Journal of cranio-maxillo-facial surgery |
| #114 | SvenW. C. Jensen | Presurgical Cleft Management of Infants: A Survey of ACPA Approved and International Cleft Palate and Craniofacial Teams | The Cleft Palate Craniofacial Journal |
| #115 | Elvire Le Norcy | Orthodontic treatment in children and adolescent patients with X-linked hypophosphatemia: A case–control study | Orthodontics and Craniofacial Research |
| #116 | Zhi-yong Zhang | Condylar resorption post mandibular distraction osteogenesis in craniofacial microsomia: A retrospective study | Journal of cranio-maxillo-facial surgery |
| #117 | Taygun Sezer | COVID-19 as a factor associated with early dental implant failures: A retrospective analysis | Clinical Implant Dentistry and Related Research |
| #118 | Hideo Shigeishi | Development and evaluation of the “ToothbrushingTimer with Information on Toothbrushes” application:Aprospective cohort pilot study | Clinical Implant Dentistry and Related Research |
| #119 | Arezoo Fallahi | Dental cleaning behavior and related factors among hemodialysis patients in theWest of Iran: A cross‐sectional study | Clinical and Experimental Dental Research |
| #120 | Kai-Fang Hu, Ying-Chu Lin | Prevalence of peri-implantitis after alveolar ridge preservation at periodontitis and nonperiodontitis extraction sites: A retrospective cohort study | Clinical Implant Dentistry and Related Research |
| #121 | Dani Stanbouly | What are the Soft Tissue Risk Factors for Obstructive Sleep Apnea in Patients with Down’s Syndrome? | The Cleft Palate Craniofacial Journal |
| #122 | Rami R. Hallac | Three-dimensional Analysis of Facial Asymmetry in Unilateral Lambdoid Craniosynostosis | The Cleft Palate Craniofacial Journal |
| #123 | Jeong-Yol Lee | Mini-implant mandibular overdentures under a two-step immediate loading protocol: A 4–6- year retrospective study | Gerodontology |
| #124 | Lydia DeJonge | Parental Perception of Oral Health and Oral Health Status of Pediatric Patients Attending an Urban Craniofacial Center | The Cleft Palate Craniofacial Journal |
| #125 | Momen A. Atieh | Changes in oral home care and smoking habits during COVID‐19 pandemic: A cross‐sectional study | Clinical and Experimental Dental Research |
| #126 | Alf Nastri | E-Scooter facial fractures: A comparative cohort study | Journal of cranio-maxillo-facial surgery |
| #127 | Elizabeth Fitriana Sari, Nicola Cirillo | General dentists' knowledge, perceptions, and practices regarding oral potentially malignant disorders and oral cancer in Indonesia | Clinical and Experimental Dental Research |
| #128 | Anthony Chu Seng Ngu | Medical profile of patients referred to an Australian postgraduate oral surgery clinic | Clinical and Experimental Dental Research |
| #129 | Jordan W. Swanson | Multimodal Treatment of Robin Sequence Utilizing Mandibular Distraction Osteogenesis and Continuous Positive Airway Pressure | The Cleft Palate Craniofacial Journal |
| #130 | Hanyao Huang | Early Cleft Palate Repair by a Modified Technique Without Relaxing Incisions | The Cleft Palate Craniofacial Journal |
| #131 | Amy L. Conrad | Screening for Academic Risk Among Students With Cleft Lip and/or Palate: Patterns of Risk and Qualities of Effective Tools | The Cleft Palate Craniofacial Journal |
| #132 | Peter A. Mossey | Core outcomes for orofacial clefts: reconciling traditional and ICHOM minimum datasets | European Journal of Orthodontics |
| #133 | Jong Woo Choi | Differences in the stability of the lesser and greater maxillary segments after cleft orthognathic surgery: A retrospective study using 3D analysis | Journal of cranio-maxillo-facial surgery |
| #134 | Damir Matic | Ten-Year Cephalomatetric Comparison of Patients With Cleft Palate who Received Treatment with Active or Passive Pre-Surgical Orthopedic Devices | The Cleft Palate Craniofacial Journal |
| #135 | Mohammed H. Elnagar | Effectiveness of AI-driven remote monitoring technology in improving oral hygiene during orthodontic treatment | Orthodontics and Craniofacial Research |
| #136 | Kittisak Sawanyawisuth | Salivary trefoil factor family peptide 3 (TFF3) and flow rate in persons with and without obstructive sleep apnea: A preliminary study | Clinical and Experimental Dental Research |
| #137 | Amrita Tembhe | Ten-year cross-sectional and longitudinal assessment and factors associated with unfavorable self-rated oral health in older adults in the United States | Gerodontology |
| #138 | Linhong Wang and Fan Yang | Evaluation of a platform-switched Morse taper connection for all-on-four or six treatment in edentulous or terminal dentition treatment: A retrospective study with 1–8 years of follow-up | Clinical Implant Dentistry and Related Research |
| #139 | Nicha Ungvijanpunya | Nasolabial morphological changes in patients with unilateral cleft lip and palate using a Korat-modified nasoalveolar moulding appliance with primary correction | Orthodontics and Craniofacial Research |
| #140 | Mohammad Younis Hajeer | The diagnostic accuracy of cone-beam computed tomography and two-dimensional imaging methods in the 3D localization and assessment of maxillary impacted canines compared to the gold standard in-vivo readings: A cross-sectional study | International Orthodontics |
| #141 | Ejvis Lamani | Genetic and other factors contributing to external apical root resorption in orthodontic patients | Orthodontics and Craniofacial Research |
| #142 | Alessandro Piccirilli | Graves-Basedow ophthalmopathy surgical approaches: Open vs Endoscopic | Journal of cranio-maxillo-facial surgery |
| #143 | Muhammad Aman | Alveolar bone loss and root resorption in mesialized second molars in mandibular first molar extraction cases as compared to contralateral non-extraction side in young adults: A retrospective cross-sectional study | International Orthodontics |
| #144 | Fabíola Bof de Andrade | Prevalence and associations of temporomandibular disorders in older Brazilian adults | Gerodontology |
| #145 | Massimo Del Fabbro | Immediate placement and loading of implants with laser-microgrooved collar in combination with an anorganic porcine bone mineral matrix in the esthetic zone. Twelvemonth results of a prospective multicenter cohort study | Clinical Implant Dentistry and Related Research |
| #146 | Fatma Karacaoglu | The relationship of peri-implant soft tissue wound healing with implant cover screw design: Cross-sectional study | Clinical Implant Dentistry and Related Research |
| #147 | Maurice J. Meade | A Cross‐Sectional Survey of the Use of Clear Aligners by General Dentists in Australia | Clinical and Experimental Dental Research |
| #148 | Yuta Nakajima | Longitudinal Morphological Changes in the Mucosal Free Margin After Unilateral Cleft Lip Repair | The Cleft Palate Craniofacial Journal |
| #149 | Matilde da Cunha Gonçalves Nojima | Influence of the maxillary dimensions and anterior teeth anatomy on the buccal impaction of upper permanent canines: A three-dimensional case-control study | International Orthodontics |
| #150 | Dalal Hammoudi Halat & Ahmed Malki | Artificial Intelligence Readiness, Perceptions, and Educational Needs Among Dental Students: A Cross‐Sectional Study | Clinical and Experimental Dental Research |
| #151 | Ritsuo Takagi | Occlusal Evaluation Using Modified Huddart and Bodenham Scoring System Following 2-Stage Palatoplasty With Hotz Plate: A Comparison Among 3 Different Surgical Protocols | The Cleft Palate Craniofacial Journal |
| #152 | Luc Van Doorne | Five years clinical outcome of maxillary mini dental implant overdenture treatment: A prospective multicenter clinical cohort study | Clinical Implant Dentistry and Related Research |
| #153 | Rafaela Scariot | Polymorphisms in hormonal-related genes might be associated with variations in permanent tooth crown size | Orthodontics and Craniofacial Research |
| #154 | Karen Gomi | Evaluation of tongue pressure and lip closing force in bimaxillary orthognathic surgery: A retrospective study | Journal of cranio-maxillo-facial surgery |
| #155 | Johannes Adrianus Smit | Decrease in Prevalence of Cleft lip, Alveolus and Palate After Nationwide Introduction of the Second-Trimester Anomaly Scan in the Netherlands | The Cleft Palate Craniofacial Journal |
| #156 | Hristina Maynalovska | Occurrence and distribution of lost molars and furcation defects in a Bulgarian populationA retrospective three‐dimensional study | Clinical and Experimental Dental Research |
| #157 | Elie Khoury | Factors influencing the perception of profile beauty in Class III dental compensationA comparative cross-sectional study according to three categories of assessors | International Orthodontics |
| #158 | Rashna Hoshang Sukhia | Comparison of mesiodistal root angulation of teeth by conventional panoramic and cone beam computed tomography images | International Orthodontics |
| #159 | Afaf Aboalrejal | Quantitative and qualitative 3D analysis of mandibular lingual concavitiesImplications for dental implant planning in the posterior mandible | Clinical and Experimental Dental Research |
| #160 | Brian E. Kinard | Does the Rigid External Distraction Device Alter Maxillary Pitch in Cleft Maxillary Distraction? | The Cleft Palate Craniofacial Journal |
| #161 | Ivna Albano Lopes | Evaluation of the Nasopalatine Canal of Patients With and Without Cleft Lip and Palate in CBCT Exams | The Cleft Palate Craniofacial Journal |
| #162 | Sotiria Gizani | Awareness and practice of dentomaxillofacial imaging among paediatric dentistsa questionnaire survey of members of the European Academy of Paediatric Dentistry | Oral Radiology |
| #163 | Busra Dilara Altun | Evaluation of Maxillary Sinus Volumes and Pathologies in Children With And Without Cleft Lip and Palate using cone beam computed tomography | Orthodontics & Craniofacial Research |
| #164 | Shin Nakamura | Risk factors for posttreatment mastoid cellulitis in patients with oral malignancy | Oral Radiology |
| #165 | Saori Yoshida | Influence of mandibular incisor agenesis and growth pattern on symphysis characteristics | Orthodontics & Craniofacial Research |
| #166 | Mengfei Yu & Huiming Wang | Endoscope-assisted maxillary sinus floor augmentation with a mini-lateral window | Clinical Implant Dentistry and Related Research |
| #167 | Natalia Vasconcellos | Association between frailty and oral health services use in Brazilian older adults | Gerodontology |
| #168 | Bing Shi & Hanyao Huang | Application of a Novel Nasal Clip for Nostril Retention After Primary Unilateral Cleft Rhinoplasty | The Cleft Palate Craniofacial Journal |
| #169 | Leonardo Paolo Demonte | Prospective study of nasal growth in bilateral cleft lip and palate patients after nasoalveolar molding and primary columella lengthening | Orthodontics & Craniofacial Research |
| #170 | Rafael Denadai | Modified Medial Incision Small Double-Opposing Z-Plasty for Treating Veau Type I Cleft Palate: Is the Early Result Reproducible? | The Cleft Palate Craniofacial Journal |
| #171 | Christian Kirschneck | Oral Cleft Related-Genes may be Involved in Root Curvature of Maxillary Lateral Incisors | The Cleft Palate Craniofacial Journal |
| #172 | Louise Crowe | The Impact of COVID-19 on the Wellbeing of Victorian Children Born with Cleft Lip and/or Palate | Orthodontics & Craniofacial Research |
| #173 | Shogo Hasegawa | Factors affecting progressive facial swelling immediately after orthognathic surgery | Journal of Cranio-Maxillo-Facial Surgery |
| #174 | Alex A. Kane | Open Versus Minimally Invasive Approach for Craniosynostosis: Analysis of the National Surgical Quality Improvement Program-Pediatric | The Cleft Palate Craniofacial Journal |
| #175 | Yonatan Reuven | Intelligence among ear deformities and cleft lip and/or palate patients during 50 years in Israel | Orthodontics & Craniofacial Research |
| #176 | Andreas Stavropoulos | Radiographic bone level and soft tissue dimensional changes following explantation of implants affected by peri-implantitis | Clinical and Experimental Dental Research |
| #177 | Xu Gong and Xuemei Gao | Anatomical phenotype of obstructive sleep apnea patients based on cluster analysis | Orthodontics & Craniofacial Research |
| #178 | Rosa María López-Pintor | Factors influencing xerostomia and oral health-related quality of life in polymedicated patients | Gerodontology |
| #179 | Ernesto B. Benalcázar-Jalkh | Prefabricated shouldered abutments enable successful restoration of molar crowns on implants | Clinical Implant Dentistry and Related Research |
| #180 | Kamlesh B. Patel | Impact of a Palatal Fistula After Cleft Palate Repair on Velopharyngeal Closure | The Cleft Palate Craniofacial Journal |
| #181 | Rana A. Al Homoud | Behavior and anxiety levels in pediatric patients: The behavioral changes and anxiety of pediatric patients in dental clinics | Clinical and Experimental Dental Research |
| #182 | Zanab Malik | Unique dietary and oral hygiene behaviors in a cohort with clinically severe obesity: A cross-sectional study | Clinical and Experimental Dental Research |
| #183 | Brenda Astrid Paz-Michel and Myriam Angelica De La Garza‐Ramos | Oral Hygiene With Neutral Electrolyzed Water and Systemic Therapy Increases Gastric Helicobacter pylori Eradication and Reduces Recurrence | Clinical and Experimental Dental Research |
| #184 | Ejvis Lamani | External apical root resorption in African American orthodontic patients | European Journal of Orthodontics |
| #185 | Parisa Soltani | Genotoxicity and Micronucleus Formation as a Result of Panoramic Radiography in Epithelial Cells of the Buccal Mucosa: A Cross‐sectional Study in Adults | Clinical and Experimental Dental Research |
| #186 | Fuming He and Xiaojun Li, | Clinical Effect of Angulated Screw Channel Crown Compared with Cement Crown | Clinical Implant Dentistry and Related Research |
| #187 | Ashita Uppoor | Cognitive Implications Among Individuals with Periodontitis and Type 2 Diabetes Mellitus | Journal of Oral Biosciences |
| #188 | Aylar Afshari | Serum Hormone Levels in Patients with Trigeminal Neuralgia Compared to Healthy Population | Clinical and Experimental Dental Research |
| #189 | Naif Ghanem | Custom-Milled Polyetherketoneketone (PEKK) Posts and Cores: A 12‐month follow‐up randomized controlled pilot study | Clinical and Experimental Dental Research |
| #190 | Lavanya Ajay Sharma | A retrospective study on the prognostic factors and success, survival, and failure outcomes of treated endodontic‐periodontal lesions | Clinical and Experimental Dental Research |
| #191 | Neda Babanouri | Prevalence and Features of Maxillary Sinus Septa in Cleft Lip and Palate Patients | The Cleft Palate Craniofacial Journal |
| #192 | Ingrid M Ganske | Dental Anomalies in Parry-Romberg Syndrome: A 40-Year Retrospective Review | The Cleft Palate Craniofacial Journal |
| #193 | Jun Wang | Effect of orthodontic treatment with premolar extractions on occlusal planes: A retrospective study | Orthodontics & Craniofacial Research |
| #194 | Amin Golshah | Relationship Between Mesial Movement of Mandibular First Molar and Angular Changes of Third Molar A retrospective comparative study in growing patients with and without first premolar extraction | International Orthodontics |
| #195 | So Young Lim | Evaluation of Long-Term Outcomes of Transverse Facial Cleft Repair | The Cleft Palate Craniofacial Journal |
| #196 | Tulika Tripathi | Association, diagnostic accuracy and optimal threshold of salivary IGF-1 and vitamin DBP levels for estimation of pubertal growth spurt | International Orthodontics |
| #197 | Anne Nordrehaug Åstrøm | Oral health inequalities in Swedish older adults over 25 years of follow-up | Gerodontology |
| #198 | Jungwon Lee and Yong-Moo Lee | Risk factors impacting the survival of implants replaced following failure | Clinical Implant Dentistry and Related Research |
| #199 | Tom Kobe | Prehydrated collagenated cortico-cancellous heterologous bone gel and papillae tunneling for isolated intrabony defects: 12‐month noninferiority trial | Clinical and Experimental Dental Research |
| #200 | Aylin Baysan | The impact of sociodemographic determinants and diabetes type-2 on oral health outcomes | Clinical and Experimental Dental Research |
| #201 | Emma Wigsten | Cost-effectiveness of root canal treatment compared with tooth extraction in a Swedish Public Dental Service | Clinical and Experimental Dental Research |
| #202 | Nelly Villacorta-Siegal | Integration of a Dental hygienist into the Interprofessional Long-Term Care Team | Gerodontology |
| #203 | Luigi Canullo | Sinus floor augmentation using crestal approach in conjunction with hydroxyapatite/cross-linked collagen sponge | Clinical Implant Dentistry and Related Research |
| #204 | Matilde da Cunha Gonçalves Nojima | Influence of the maxillary dimensions and lateral incisor anatomy on the palatal impaction of maxillary permanent canines | International Orthodontics |
| #205 | Carole Charavet | What are the prevalence and risk factors associated with wire syndrome in dental students? A cross-sectional study | International Orthodontics |
| #206 | Josip Tomic | Heparin Regimes in Head and Neck Cancer Flap Surgery: A Retrospective Cohort Study of free flap complications graded by Clavien-Dindo | Journal of Cranio-Maxillo-Facial Surgery |
| #207 | Hari Petsos | Long-Term Stability of Infrabony Defects Treated with Enamel Matrix Derivate Alone: A retrospective two-centre cohort study | Journal of Clinical Periodontology |
| #208 | Katelyn J. Kotlarek | Impact of Prenatal Care on Newborn Complications for Infants with Cleft Lip with or without Cleft Palate | Cleft Palate Craniofacial Journal |
| #209 | Ruben P. Houkes | Unraveling a Major Burden of Orofacial Clefts Analyses: Classification of Cleft Palate Fistulas by Cleft Surgeons | Cleft Palate Craniofacial Journal |
| #210 | Yaniv Mayer | The influence of radio frequency‐based toothbrush on the accumulation of calculus and periodontal health: A randomized double‐blind controlled prospective study | Clinical and Experimental Dental Research |
| #211 | Robert J Schroth | Prevalence of Treatment of Early Childhood Caries among Children with Cleft Lip and/or Cleft Palate in Manitoba | Cleft Palate Craniofacial Journal |
| #212 | Petros Papaefthymiou | Correlation of Dental Anomalies with Cleft Type and Gender in Non-Syndromic Oral Cleft Patients: A Cross-Sectional Study | Cleft Palate Craniofacial Journal |
| #213 | Yu Wang and Mengfei Yu | Adjacent tooth migration after maxillary first molar loss in patients with sinus augmentation: A retrospective research | Clinical Implant Dentistry and Related Research |
| #214 | Rami R. Hallac | Alar Asymmetry in Patients with Unilateral Cleft Lip: Implications for Secondary Rhinoplasty | Cleft Palate Craniofacial Journal |
| #215 | Susan L. Thibeault | Relationship Between Auditory-Perceptual and Objective Measures of Resonance in Children with Cleft Palate: Effects of Intelligibility and Dysphonia | The Cleft Palate Craniofacial Journal |
| #216 | Sophia A.J. Kronig | UCSQ Method Applied on 3D Photogrammetry: Non-Invasive Objective Differentiation Between Synostotic and Positional Plagiocephaly | The Cleft Palate Craniofacial Journal |
| #217 | Radwan Algabri | Behaviors, hygiene habits, and sources of care among removable complete and partial dentures wearers: A multicenter cross‐sectional study | Clinical and Experimental Dental Research |
| #218 | Meryem Kaygısız Yiğit | Dental radiographic changes in individuals with COVID‑19: a controlled retrospective study | Oral Radiology |
| #219 | Claudia Trindade Mattos | Malocclusion or Laterorhinia: Which One Impacts Aesthetic Facial Perception the Most? | Orthodontics & Craniofacial Research |
| #220 | Laura Kaprio | Non-Progressive Mandibular Changes in Children with Type I and II Craniofacial Microsomia | Orthodontics & Craniofacial Research |
| #221 | Masahiro Takahashi | Comparison of Maxillofacial Morphology Between Modified Furlow’s and Modified two-Flap Palatoplasty in Orofacial Clefts During the Primary Dentition Period | The Cleft Palate Craniofacial Journal |
| #223 | Christina L. Nicholas | Comparing the Relative Influence of Obesity and Ancestry on Timing of Dental Development | Orthodontics & Craniofacial Research |
| #224 | Claudine Le Vaillant | Can a Predetermined Grid and Multidisciplinary Consultation Improve the Description of Cleft Lip with or Without Cleft Palate? | The Cleft Palate Craniofacial Journal |
| #225 | Célia Regina Maio Pinzan-Vercelino | Relationship Between Dentofacial Features and Bullying in Schoolchildren | Pediatric Dentistry |
| #226 | Spyridon N. Papageorgiou | Genetic and environmental impact on mandibular growth in mono- and dizygotic twins during adolescence | International Orthodontics |
| #227 | Sema Kaya | Radiologic evaluation of associated symptoms and fractal analysis of unilateral dens invaginatus cases | Oral Radiology |
| #228 | Minkook Son | Chronic periodontitis and risk of cerebro-cardiovascular diseases among older Koreans | Gerodontology |
| #229 | Shivam Metha | Positional, morphologic, and volumetric differences in TMJ in unilateral posterior crossbites and controls | International Orthodontics |
| #230 | Maria Saadeh | Dentoalveolar bone height in Class I adults with different vertical patterns | International Orthodontics |
| #231 | Andrew May | Measuring the Change in Soft Palate Length and Shape Following Maxillary Advancement: A Cohort Study in Patients with Orofacial Clefts | The Cleft Palate Craniofacial Journal |
| #232 | Jordan W Swanson | Geospatial and Socioeconomic Factors Interact to Predict Management and Outcomes in Cleft Lip and Palate Surgery: A Single Institution Study of 740 Patients | The Cleft Palate Craniofacial Journal |
| #233 | Sergio Henrique Kiemle Trinidade | Assessment of Internal Nasal Dimensions of Individuals With Cleft Lip and Palate and Obstructive Sleep Apnea Syndrome by Computed Tomography | The Cleft Palate Craniofacial Journal |
| #234 | Robin Yang | Pediatric Orthognathic Surgery: A NSQIP-P Comparison of Peri-Operative Factors and Outcome Differences Between Cleft and Noncleft Patients | The Cleft Palate Craniofacial Journal |
| #235 | Jayakumar Jayaraman | A Survey of Attitudes and Training of Pediatric Dentistry Residents and Faculty in the US Regarding LGBTQ Patient Population | Pediatric Dentistry |
| #236 | Johanna A. Gaß | Ceramic Anterior Veneer Restorations in Periodontally Compromised Patients | Clinical Advances in Periodontics |
| #237 | Pedro Henrique Sette-de-Souza | Access to Dental Services in an Elder Population of African Descent in Brazil | Gerodontology |
| #238 | Walaa Kadry | A Novel Technique Predicting Velopharyngeal Insufficiency Risk in Newborns Following Primary Cleft Repair A randomized clinical trial comparing buccinator flap and Bardach two-flap palatoplasty | Journal of Cranio-Maxillo-Facial Surgery |
| #239 | Quentin Hennocq | AI-based Diagnosis and Phenotype-Genotype Correlations in Syndromic Craniosynostoses | Journal of Cranio-Maxillo-Facial Surgery |
| #240 | Silvia Amélia Scudeler Vedovello | Three-Dimensional Analysis of Posterior Tooth Inclinations in Transverse and Sagittal Relationships | International Orthodontics |
| #241 | Marta Cajozzo | 25-year Follow-up of Primary Tibial Periosteal Graft for Hard Palate Repair in Cleft Lip and Palate: Outcomes, Concerns and Controversies | The Cleft Palate Craniofacial Journal |
| #242 | Gavriel Chaushu | The Impact of Antiplatelet and Anticoagulant Medications on Early Implant Failure Following Sinus Floor Augmentation | Clinical Implant Dentistry and Related Research |
| #243 | Ricardo Guimaraes Fischer | Histomorphometric and microtomographic analysis of maxillary sinus augmentation surgeries with and without collagen membrane on the lateral window: A randomized clinical trial | Clinical Implant Dentistry and Related Research |
| #244 | Ana Carolina Del-Sarto Azevedo Maia | Association Between Sex of the Individual With Untreated Cleft Lip and Social Judgment Made by Lay Persons | Orthodontics & Craniofacial Research |
| #245 | Nooshin Mohtasham | Downregulation of salivary miR‐3928 as a potential biomarker in patients with oral squamous cell carcinoma and oral lichen planus | Clinical and Experimental Dental Research |
| #246 | Geoff Frawley | Prediction of Early Postoperative Pain in Infants Undergoing Primary Cleft Palate Repair | The Cleft Palate Craniofacial Journal |
| #247 | Ioannis Polyzois | A longitudinal analysis of the impact of nonsurgical and surgical treatment of peri‐implantitis upon clinical parameters and implant stability quotient values. A 2–3‐year follow‐up | Clinical and Experimental Dental Research |
| #248 | Bruno César de Vasconcelos Gurgel | Retrospective assessment of patients' risk for peri‐implant diseases using the implant disease risk assessment (IDRA) tool: A cohort study | Clinical Implant Dentistry and Related Research |
| #249 | Sara Kinter | Does Distraction Lower Risk of VPI Compared to Conventional Maxillary Advancement? A Retrospective Cohort Study of Adolescents with Cleft Palate | The Cleft Palate Craniofacial Journal |
| #250 | Tamaki Nakano | Multivariate analysis of the effect of keratinized mucosa on peri‐implant tissues with platform switching: A retrospective study | Clinical Implant Dentistry and Related Research |
| #251 | Giuseppe Troiano | Can radiomic features extracted from intra‐oral radiographs predict physiological bone remodelling around dental implants? A hypothesis‐generating study | Journal of Clinical Periodontology |
| #252 | Sotiria Gizani | Exposure and protection protocols of dentomaxillofacial imaging applied by paediatric dentists in Europe | Oral Radiology |
| #253 | Wenhui Wang | Epidemiology of plaque‐induced gingivitis among 12–15‐year‐old Chinese schoolchildren: A study based on the 2018 case definition | Journal of Clinical Periodontology |
| #254 | Elif Inonu | Assessment of palatal mucosal thickness and location of the greater palatine foramen using cone-beam computed tomography: a retrospective study | Oral Radiology |
| #255 | Jong Woo Choi | Unipolar myomectomy for congenital muscular torticollis: A retrospective study | Journal of Cranio-Maxillo-Facial Surgery |
| #256 | Tamás Würsching | Alveolar cleft reconstruction utilizing a particulate autogenous tooth graft and a novel split-thickness papilla curtain flap — A retrospective study | Journal of Cranio-Maxillo-Facial Surgery |
| #257 | Arun K. Gosain | Predictors of Adverse Outcomes Following Cleft Palate Repair: An Analysis of Over 2500 Patients Using International Smile Train Data | The Cleft Palate Craniofacial Journal |
| #258 | Mai Fujimoto | Three-dimensional Analysis of Factors Related to the Effective Alveolar Molding in Presurgical Infant Orthopedics: Findings From a Pilot Study | The Cleft Palate Craniofacial Journal |
| #259 | Xi Jiang | The facial–coronal ridge crest alterations after single immediate implant placement and provisionalization with thin buccal plate phenotype in anterior maxilla: A radiographic case‐series study | Clinical Implant Dentistry and Related Research |
| #260 | Charline Mourgues | Comparison of the microvascular anastomotic Coupler™ system with hand-sewn suture for end to end veno-venous anastomosis for head and neck reconstruction with free flap transfer: Medico-economic retrospective case-control study | Journal of Cranio-Maxillo-Facial Surgery |
| #261 | Roberto L. Flores | Anatomical Study of Domain Rescue of Palatal Length in Patients With a Wide Cleft Palate: Buccal Flap Reconstruction in Primary Palatoplasty | The Cleft Palate Craniofacial Journal |
| #262 | Klaus M. Friedrich | Radiological follow-up of cemento-osseous dysplasia on cone-beam computed tomography | Journal of Cranio-Maxillo-Facial Surgery |
| #263 | Shariel Sayardoust | Clinical and radiographic periodontal status in hypertensive patients with or without obstructive sleep apnea 10 years after diagnosis and CPAP initiation | Clinical and Experimental Dental Research |
| #264 | Ilgin Ari | Incidence, treatment method and recurrence rate in giant cell granulomas: Retrospective study | Journal of Cranio-Maxillo-Facial Surgery |
| #265 | Sara Eslami | Evaluation of thickness of 3D printed versus thermoformed aligners: A prospective in vivo ageing experiment | Orthodontics & Craniofacial Research |
| #266 | Mark A. Green | Does Differential Maxillary Expansion Prior to Alveolar Cleft Bone Grafting Affect Nasal Width? | The Cleft Palate Craniofacial Journal |
| #267 | Alyssa Valenti | A Retrospective Analysis of the Impact of Health Disparities on Treatment for Single Suture Craniosynostosis Before and During the Pandemic | The Cleft Palate-Craniofacial Journal |
| #268 | Anggun Rafisa | Age at primary surgery among orofacial cleft individuals in Indonesia | Orthodontics & Craniofacial Research |
| #269 | Antonio Magan-Fernandez | Detection and comparison of neutrophil extracellular traps in tissue samples of peri-implantitis, periodontitis, and healthy patients: A pilot study | Clinical Implant Dentistry and Related Research |
| #270 | Yang Liu and Xin Xiong | Association of temporomandibular joint osteoarthrosis with dentoskeletal morphology in males: A cone-beam computed tomography and cephalometric analysis | Orthodontics & Craniofacial Research |
| #272 | Eran Gabay | Tissue changes around dental implants installed in alveolar ridge preservation sites: A 1-year follow-up randomized controlled clinical trial | Clinical Implant Dentistry and Related Research |
| #273 | Michael Schwaiger | Surgical site infections in maxillofacial trauma surgery – Single-dose versus prolonged antibiotic prophylaxis | Journal of Cranio-Maxillo-Facial Surgery |
| #274 | R. A. Vernucci | Study of the maxillary yaw on cone beam computed tomography: A preliminary report and comparison between two different dento-skeletal malocclusions | Orthodontics & Craniofacial Research |
| #275 | Maximilian Riekert | Prophylactic removal of titanium osteosynthesis miniplates in patients after midface fractures - A retrospective cohort study | Journal of Cranio-Maxillo-Facial Surgery |
| #276 | Maria Cadenas de Llano-Pérula | Three-dimensional evaluation of secondary alveolar bone grafting in patients with unilateral cleft lip and palate: A 2–3 year post-operative follow-up | Orthodontics & Craniofacial Research |
| #277 | Anna Seidel | Three-dimensional imaging analysis of CAD/CAM custom-milled versus prefabricated allogeneic block remodelling at 6 months and long-term follow-up of dental implants: A retrospective cohort study | Journal of Clinical Periodontology |
| #278 | C. M. Rivers | Use of Hyaluronic Acid Filler in Patients with Secondary Cleft Lip Deformity | The Cleft Palate Craniofacial Journal |
| #279 | Megan Pencek | Simultaneous Furlow Palatoplasty and Tonsillectomy for the Treatment of Velopharyngeal Insufficiency and Tonsillar Hypertrophy | The Cleft Palate Craniofacial Journal |
| #280 | Tae Suk Oh | Primary Cleft Rhinoplasty with Custom Suture Needle for Alar Cartilage Repositioning in Unilateral Cleft Lip Patients: 3D Nasal Analysis | The Cleft Palate Craniofacial Journal |
| #281 | X. M. Xu | Risk factors for gingival invagination: A retrospective study | Journal of Clinical Periodontology |
| #282 | Jordan W Swanson | Characterizing Interventions and Family Assistance of a Nurse Navigation Program in Orofacial Cleft Care | The Cleft Palate Craniofacial Journal |
| #283 | Maria Grazia Piancino | Functional treatment of bilateral posterior crossbite improves mandibular kinematics during chewing | Orthodontics & Craniofacial Research |
| #284 | Cameron L. Randall | Pediatric Dentists’ Use of Nonpharmacological Behavior Guidance Techniques and Experiences with Caregiver Acceptance: A National Survey | Pediatric Dentistry |
| #285 | Kamlesh B. Patel | Porous Polyethylene Versus Autologous Costochondral Reconstruction for Microtia: Incidence and Analysis of Secondary Procedures | The Cleft Palate Craniofacial Journal |
| #286 | Usama S. Hamdan | Single-Stage Posterior Vomerine Ostectomy and Primary Cheiloplasty in Patients with Bilateral Cleft Lip & Palate and Protuberant Premaxilla | The Cleft Palate Craniofacial Journal |
| #287 | Alessandra Lucchese | How well does Invisalign ClinCheck predict actual results: A prospective study | Orthodontics & Craniofacial Research |
| #289 | Marco Farronato | Divergence between CBCT and Optical Scans for Soft Tissue Analysis and Cephalometry in Facial Imaging: A cross-sectional study on healthy adults | International Orthodontics |
| #290 | Mirjana Bošković | The Influence of Removable Complete Denture on Pro‐Oxidant Antioxidant Balance and Redox‐Sensitive Inflammation Biomarker NF‐ĸB in the Oral Cavity: An Interventional Follow‐Up Study | Clinical and Experimental Dental Research |
| #291 | Szofia Szentpeteri | Examination of certain single-nucleotide polymorphisms of interleukins 1A and 1B in medication-related osteonecrosis of the jaw — An ambirectional cohort study | Journal of Cranio-Maxillo-Facial Surgery |
| #292 | Lenka Kožejová Jaklová | Morphometric Assessment of Facial Morphology in Infants with Orofacial Clefts up to two Years of Age: A Three-Dimensional Cross-Sectional Study | The Cleft Palate Craniofacial Journal |
| #293 | S. Alex Rottgers | An Algorithmic Approach for Deploying Buccal Fat Pad Flaps and Buccal Myomucosal Flaps Strategically in Primary and Secondary Palatoplasty | The Cleft Palate Craniofacial Journal |
| #294 | Caroline Carrico | Effect of guided implant placement learning experiences on freehand skills: A pilot study | Clinical and Experimental Dental Research |
| #295 | Gaia Pellegrini | Maxillary sinus lift augmentation: A randomized clinical trial with histological data comparing deproteinized bovine bone grafting vs graftless procedure with a 5–12-year follow-up | Clinical Implant Dentistry and Related Research |
| #296 | Michael Alperovich | Orthotic Helmet Therapy for Deformational Plagiocephaly: Stratifying Outcomes by Insurance | The Cleft Palate Craniofacial Journal |
| #297 | Hani ElNahass | Evaluation of buccal bone resorption in immediate implant placement in thin versus thick buccal bone plates: An 18-month follow-up prospective cohort study | Clinical Implant Dentistry and Related Research |
| #298 | Michelle Briner | Cranio-Cervical Abnormalities in Moderate to Severe Osteogenesis Imperfecta – Genotypic and Phenotypic Determinants | Orthodontics and Craniofacial Research |
| #299 | M.S. Muthu | Genetic variations in exon 10 of ENAM and their association with early childhood caries | Journal of Oral Biosciences |
| #300 | Ricardo Grillo | Frontal facial analysis of female celebrity attractiveness standards through artificial intelligence | Journal of Cranio-Maxillo-Facial Surgery |
| #301 | Ana Carrillo de Albornoz | Treatment of peri‐implantitis with a flapless surgical access combined with implant surface decontamination and adjunctive systemic antibiotics: A retrospective case series study | Journal of Clinical Periodontology |
| #302 | Claudius Steffen | Patient-specific 3D-printed mini-versus reconstruction plates for free flap fixation at the mandible: Retrospective study of clinical outcomes and complication rates | Journal of Cranio-Maxillo-Facial Surgery |
| #303 | Zhongqi Liu and Minghui Cao | Modified intraoperative temperature management prevents prolonged length of stay after head and neck surgery with free flap reconstruction | Journal of Cranio-Maxillo-Facial Surgery |
| #304 | Masaru Konishi | Retrospective analysis of tooth extraction and osteoradionecrosis after low-dose rate brachytherapy for patients with tongue cancer | Oral Radiology |
| #305 | Jingping Zhou | Long-term morphometric changes in the anterior alveolar bone in adolescents and adults after space closure: A retrospective study | Orthodontics and Craniofacial Research |
| #306 | Eric Johansson | Quality of Life After Orthognathic Surgery in Swedish Patients: A Register‐Based Cohort | Clinical and Experimental Dental Research |
| #307 | Stratos Vassis | Effects of orthopedic treatment (distraction splint) on dentofacial deformities in patients with juvenile idiopathic arthritis | European Journal of Orthodontics |
| #308 | Arezoo Aghakouchakzadeh | Evaluation of the relationship between the expression of AgNOR and Ki67 with the recurrence rate in central granulomatous giant cell lesions: A case‐control | Clinical and Experimental Dental Research |
| #309 | Xiao Yang and Min Zhu | Abnormal tooth maturation associated with port wine stains | Orthodontics and Craniofacial Research |
| #310 | Seema Gupta | Comparison of gingival thickness by CBCT versus transgingival probing and estimation of cut-off values for gingival phenotype – A cross-sectional study in adults | International Orthodontics |
| #311 | Nikos Mattheos | Association of Peri‐Implant Mucosa Dimensions With Emergence Profile Angles of the Implant Prosthesis | Clinical and Experimental Dental Research |
| #312 | Koichiro Matsuo | Changes in oral health status with dental intervention during the acute to subacute stages of stroke | Gerodontology |
| #313 | Aysooda Afshari | Conventional maxillary denture versus maxillary implant‐supported overdenture opposing mandibular implant‐supported overdenture: Patient's satisfaction | Clinical and Experimental Dental Research |
| #314 | Junya Nakamura | Analysis of post‐extraction bleeding in patients taking antithrombotic therapy using data from the longevity improvement and fair evidence study | Gerodontology |
| #315 | Ivy Kiemle Trindade-Suedam | Bite Force, Masticatory Performance, and Nutritional Status of Adult Individuals With Treacher Collins Syndrome | The Cleft Palate Craniofacial Journal |
| #316 | Aikaterini Bini | Midface microvascular reconstruction after maxillary complex tumor resection: A retrospective study | Journal of Cranio-Maxillo-Facial Surgery |
| #317 | Julian Woolley | Usage of temporary anchorage devices: A cross-cultural and cross-sectional survey of orthodontists in Australia and the UK | International Orthodontics |
| #318 | Diana Heimes | Comparison of morbidity‐related parameters between autologous and allogeneic bone grafts for alveolar ridge augmentation from patients' perspective—A questionnaire‐based cohort study | Clinical Implant Dentistry and Related Research |
| #319 | Maurice J. Meade | Planned and achieved overjet and overbite changes following an initial series of Invisalign aligners: A retrospective study of adolescent patients | International Orthodontics |
| #320 | Jianping Zhou | Multivariate analysis of alveolar bone dehiscence and fenestration in anterior teeth after orthodontic treatment: A retrospective study | Orthodontics and Craniofacial Research |
| #321 | Zhiyong Zhang | Three-dimensional Analysis of the Temporal Bone Morphology in Patients with Craniofacial Microsomia | The Cleft Palate Craniofacial Journal |
| #322 | Ana Paula Fukushiro | Effect of Orthognathic Surgery on Breathing in Patients with Cleft Lip and Palate: 20-Year Experience at a Tertiary Hospital in Brazil | The Cleft Palate Craniofacial Journal |
| #323 | Zeynep Betül Arslan | Evaluation of the Thickness and Internal Structure of the Masseter Muscle with Ultrasonography in Female Bruxism Patients | Oral Radiology |
| #324 | Clarissa Schneider | Dental Injuries in Swiss Soccer Supporters: A Comparative Study of Regular Fans, Ultras, and Hooligans for Public Health Strategies | Clinical and Experimental Dental Research |
| #326 | Nirdhum Shikha | Simplified Practice for Ophthalmologist Consultation in Malar Fractures: A Retrospective Study | Journal of Cranio-Maxillo-Facial Surgery |
| #328 | Lutgart De Ridder | Initial Development of an ‘Orthodontic Care Index’ Involving Treatment Need, Complexity, and Priority | European Journal of Orthodontics |
| #329 | Shailaja Raghavan | Unboxing the Reality: Knowledge, Attitude, and Perception of Orthodontists, Dental Practitioners, and Orthodontic Patients Toward Mouthguards' Wear During Contact Sports | Clinical and Experimental Dental Research |
| #330 | Sarah Abu Arqub | Assessment of the Occlusal Outcomes in Patients Treated with Orthognathic Surgery and Clear Aligners | Orthodontics and Craniofacial Research |
| #332 | Ahmet Emin Demirbas | Which Factors Affect the Lingual Fracture Pattern in Sagittal Split Ramus Osteotomy? | Journal of Cranio-Maxillo-Facial Surgery |
| #334 | Serpil Çokakoğlu | Stability and Failure Rate During 3 Years of Fixed Retention: A Follow-up of a Randomized Clinical Trial on Adolescents with Four Different Lingual Retainers | Orthodontics and Craniofacial Research |
| #335 | Carlos Flores-Mir | The effect of the dental follicle volume of palatally impacted canines on the relative position of the adjacent teeth | European Journal of Orthodontics |
| #336 | Christian Mertens | Full block or split block?—Comparison of two different autogenous block grafting techniques for alveolar ridge reconstruction | Clinical Implant Dentistry and Related Research |
| #337 | Nikolaos Gkantidis | Third molar agenesis in individuals with supernumerary teeth | Orthodontics & Craniofacial Research |
| #338 | Y. Lei | Three-dimensional analysis of mandibular morphology asymmetry and temporomandibular joint position in patients with unilateral Brodie bite | Orthodontics & Craniofacial Research |
| #339 | Aravind Kumar Subramanian | Comparison of treatment effects during en‐masse retraction of upper anterior teeth placed using mini‐implants at infrazygomatic crest and interradicular sites: A randomized controlled trial | Orthodontics & Craniofacial Research |
| #340 | Daniella Torres Tagawa | Temporomandibular joint disc position and shape in patients submitted to two protocols of rapid maxillary expansion and face mask therapy: A randomized clinical trial | Orthodontics & Craniofacial Research |
| #341 | Colin Fuller | Outcomes After Pharyngeal Flap Surgery in Children: A Comparison of Lined Versus Unlined Flaps | The Cleft Palate Craniofacial Journal |
| #342 | Qian Liu | Incidence, severity, and risk factors for white spot lesions in adolescent patients treated with clear aligners | Orthodontics & Craniofacial Research |
| #343 | Koichi Otsuki | Maxillary Development and Dental Arch Relationships Following Early Two-Stage Palatoplasty: A Comparative Study | The Cleft Palate Craniofacial Journal |
| #344 | Esra Bolat Gümüş | Evaluation of mandibular trabecular bone structure in growing children with Class I, II, and III malocclusions using fractal analysis: A retrospective study | International Orthodontics |
| #345 | Akif Demirel | A Retrospective Investigation of Patient- and Procedure-Related Factors Associated with Cardiorespiratory Complications in Pediatric Dental Patients Undergoing Deep Sedation | Pediatric Dentistry |
| #346 | Orlando Luiz do Amaral Júnior | Preventive Dental Service Use and Oral Health-Related Quality of Life in Brazilian Older Adults | Gerodontology |
| #347 | Daniel Troeltzsch | Clinical and Social Success in Epithetic Ear Rehabilitation – Retrospectively Evaluating the Long-Term Survival of Auricular Implants | Journal of Cranio-Maxillo-Facial Surgery |
| #348 | Yunpeng Li and Junrui Zhang | Comprehensive Repair of the Alveolar Cleft Using Cortical and Cancellous Bone Layers: A Retrospective Study | Journal of Cranio-Maxillo-Facial Surgery |
| #349 | Tong Wah Lim | Evaluating the Patient Sociodemographic Factors Affecting Dental Students' Clinical Communication Skills Using a Three-Perspective Approach | Clinical and Experimental Dental Research |
| #350 | Jennifer Luca | Estimated Carbon Emissions Associated With Dental Treatment for Early Childhood Caries | Pediatric Dentistry |
| #351 | Lucete Fernandes Færøvig | Mandibular Second Molar Extraction: A Retrospective Cohort Study of Spontaneous Occlusal Changes in Adolescent Patients | Orthodontics & Craniofacial Research |
| #352 | Dekel Shilo | Comparison of Patient-Specific Implant Reconstruction vs Conventional Titanium Mesh Reconstruction of Orbital Fractures Using a Novel Method | Journal of Cranio-Maxillo-Facial Surgery |
| #353 | Minna Merta | Skeletal Stability After Mandible Bilateral Sagittal Split Osteotomy – Comparison of Patient-Specific Implant and Mini-Plate Fixation: A Retrospective Study | Journal of Cranio-Maxillo-Facial Surgery |
| #354 | S. Alex Rottgers | A Survey of Bone Grafting Practice Patterns in North American Cleft Surgeons | The Cleft Palate Craniofacial Journal |
| #355 | José Guimarães-Ferreira | Tranexamic acid decreases transfusion requirements during the modified pi-plasty procedure for sagittal craniosynostosis | Journal of Cranio-Maxillo-Facial Surgery |
| #356 | Snehlata Oberoi | Comparison of Alveolar Bone Grafting Outcomes using CBCT in Individuals with UCLP Based on the Presurgical Orthodontic Treatment Methods | The Cleft Palate Craniofacial Journal |
| #357 | Subhendu Khan | Outcome of Nasal Layer Reinforcement With Autologous Dermis in Cleft Palate Repair on Postoperative Fistula Formation | The Cleft Palate Craniofacial Journal |
| #358 | Spyridon N. Papageorgiou | Comparative assessment of two-phase class II treatment with Activator or Bionator followed by fixed appliances: A retrospective controlled before-and-after study | International Orthodontics |
| #359 | Yan Yan | Associations between activities of daily living, leisure activities and subjective well-being in Chinese older adults | Gerodontology |
| #360 | Yasmein Maher El-beblawy | Accuracy of formula-based volume and image segmentation-based volume in calculation of preoperative cystic jaw lesions’ volume | Oral Radiology |
| #361 | Cecilia Hedmo | Evaluation of the aesthetics and clinical findings in patients with missing maxillary lateral incisors treated with a 10-year interval | European Journal of Orthodontics |
| #362 | Oksana A, Jackson | Speech Outcomes Following Operative Management of Velopharyngeal Dysfunction (VPD) in Non-Syndromic Post-Palatoplasty Cleft Palate Patients | Journal of Cranio-Maxillo-Facial Surgery |
| #363 | Janice Cheah Ping Chuang | Singapore dentists' attitudes toward dental care provision for older adults with disabilities | Gerodontology |
| #364 | Peer W. Kämmerer | A novel alloplastic grid reconstruction plate for the mandible – Retrospective comparative clinical analysis of failure rates and specific complications | journal of cranio-Maxillo-Facial Surgery |
| #365 | Shoushan Bu | Streamlining complex mandibular fracture treatment: Integration of virtual surgical planning and short-segment drilling guides | Journal of Cranio-Maxillo-Facial Surgery |
| #366 | Jamal Giri | Genetic and environmental contributions to the development of soft tissue facial profile: a twin study | European Journal of Orthodontics |
| #367 | Susanna Botticelli | Apical incisor root resorption due to ectopic maxillary canine eruption: Association with time of diagnosis and type of intervention | Orthodontics & Craniofacial Research |
| #368 | Nancy L. Ford | Measuring the outcomes of lateral ridge augmentation using cone-beam computed tomography | Clinical Implant Dentistry and Related Research |
| #369 | Yan Wu | The short- and long-term effects of congenital occlusion loss of the unilateral first permanent molar on the temporomandibular joint morphology and position | Orthodontics & Craniofacial Research |
| #370 | Kasper Stokbro | Accuracy and stability of the condyle position after orthognathic surgery: A retrospective study | Journal of Cranio-Maxillo-Facial Surgery |
| #371 | Bin Yan | Effect of socioeconomic and malocclusion-related factors on duration of orthodontic treatment by fixed appliance | Orthodontics & Craniofacial Research |
| #372 | Abdolreza Jamilian | Three-dimensional evaluation of mandibular lingula: Comparisons of skeletal angle classifications and growth patterns | Orthodontics & Craniofacial Research |
| #374 | Pierre Tawa | Safety and reliability of the internal jugular vein for venous anastomoses in head and neck oncological reconstruction: A retrospective study | Journal of Cranio-Maxillo-Facial Surgery |
| #375 | Jamie L. Funamura | Utility of Screening for Obstructive Sleep Apnea with the Pediatric Sleep Questionnaire (PSQ) in Children with Craniofacial Anomalies | The Cleft Palate Craniofacial Journal |
| #376 | Reem Alansari | Why do patients transition between orthodontic appliances? A qualitative analysis of patient decision-making | Orthodontics & Craniofacial Research |
| #377 | Simona Barone | Reliability of magnetic resonance for temporomandibular joint disc perforation: A 12 years retrospective study | Journal of Cranio-Maxillo-Facial Surgery |
| #379 | Lateefa Alkharafi | Seasonal, Geographic, and Ethnic Influence on the Prevalence of Orofacial Clefts in Kuwait: A Nationwide Study | The Cleft Palate Craniofacial Journal |
| #380 | Yong-Il Kim | Comparison of the skeletodental effects of miniscrew‐anchored and tooth‐anchored facemask treatment in growing patients with skeletal class III malocclusions | Orthodontics & Craniofacial Research |
| #381 | Christine Chiao | Accuracy of Parent-Reported Allergies and Medications in Pediatric Dentistry | Pediatric Dentistry |
| #382 | Andrea Roccuzzo | Long‐term treatment outcomes of single maxillary buccal peri‐implant soft tissue dehiscences: A 10‐year prospective study | Clinical Implant Dentistry and Related Research |
| #383 | Nobuhiro Yamakawa | Volume-based 18F-fluorodeoxyglucose positron emission tomography/computed tomography parameters correlate with delayed neck metastasis in clinical early-stage oral squamous cell carcinoma | Oral Radiology |
| #384 | Andrea Rombolà | Evaluation of effects of brackets and orthodontic wires on intraoral scans: A prospective in‐vivo study | Orthodontics & Craniofacial Research |
| #386 | Kai Yang | Loss of attachments in patients during orthodontic therapy with clear aligners: A prospective clinical study | Orthodontics & Craniofacial Research |
| #387 | Gregoire Longchamp | Predictors of length of stay following maxillofacial abscess surgery: A 10-year retrospective cohort | Journal of Cranio-Maxillo-Facial Surgery |
| #388 | Dashnor Bukleta | Comparison of the impact of two types of removable partial dentures on the periodontal health of the remaining teeth: A prospective clinical study | Clinical and Experimental Dental Research |
| #389 | Tae Suk Oh | Effect of Acellular Dermal Matrix on Long-Term Speech Outcomes in Primary Palatoplasty with Radical Intravelar Veloplasty | The Cleft Palate Craniofacial Journal |
| #391 | Pradip R. Shetye | Defining the Treatment Gap in Nasoalveolar Molding: Factors Affecting the Utilization of NAM in an Urban Cleft Center | The Cleft Palate Craniofacial Journal |
| #392 | Luis Ernesto Arriola-Guillén | Alveolar morphometry in young adults with and without agenesis of the upper lateral incisor: A retrospective study | International Orthodontics |
| #393 | Adrian Philipp Emanuel Balbi | Comparing the characteristics of recurrent versus primary odontogenic keratocysts – A single center retrospective cohort study | Journal of Cranio-Maxillo-Facial Surgery |
| #394 | P. Sexton | The necessity of removal of third molars involved in mandibular fractures: A retrospective study | Journal of Cranio-Maxillo-Facial Surgery |
| #395 | Rosa Esposito | Skeletal and dental effects of function-generating bite appliance compared to rapid palatal expander and untreated controls | Orthodontics & Craniofacial Research |
| #396 | Stacey H. Francis | Risk Factors for Delayed Diagnosis of Positional Plagiocephaly: A Review of 25,322 Patients | The Cleft Palate Craniofacial Journal |
| #397 | Zhiwei Jiang, Guoli Yang | In vitro and in vivo accuracy of autonomous robotic vs. fully guided static computer‐assisted implant surgery | Clinical Implant Dentistry and Related Research |
| #398 | Maryam Paknahad | The effect of tooth loss on the temporomandibular joint space: A CBCT study | Clinical and Experimental Dental Research |
| #399 | João Nunes Pombo | Metopic craniosynostosis: Dynamic cranioplasty for trigonocephaly versus fronto-orbital remodeling and advancement — A retrospective study | Journal of Cranio-Maxillo-Facial Surgery |
| #400 | Luis Ernesto Arriola-Guillén | Association of mandibular canine impaction and anatomical factors: A multicentre retrospective panoramic study in Latin America | International Orthodontics |
| #402 | Edoardo Mannelli | Is a non-radiological-assisted method valid for establishing crown-root relationships in an orthodontic set-up incorporating the roots? A retrospective study | International Orthodontics |
| #403 | Hongfei Liao, Yaohua Wang | Initial implementation of surgical guide design utilizing digital medicine for lateral orbital decompression surgery | Journal of Cranio-Maxillo-Facial Surgery |
| #404 | Kiichiro Yaguchi | Addition of a Buccinator Musculomucosal Flap Improves Surgical Outcomes of Conventional two-Flap Palatoplasty: A Comparative Study | The Cleft Palate-Craniofacial Journal |
| #405 | Phayvanh P Pecha | Feeding Performance and Outcomes in Infants With Robin Sequence Undergoing Mandibular Distraction Osteogenesis | The Cleft Palate-Craniofacial Journal |
| #406 | Daiki Mor | Automatic orbital segmentation using deep learning-based 2D U-net and accuracy evaluation: A retrospective study | Journal of Cranio-Maxillo-Facial Surgery |
| #407 | Jennifer M. Kalish, Jesse A. Taylor | Associations between the timing of tongue reduction surgery, (Epi) genotype, and dentoskeletal development in patients with Beckwith-Wiedemann syndrome | Journal of Cranio-Maxillo-Facial Surgery |
| #408 | Rosemary Sadami Arai Shinkai | Long-term clinical performance of short 6-mm implants supporting single crowns in the posterior region: A 10-year cohort study | Clinical Implant Dentistry and Related Research |
| #409 | Miodrag Šćepanović | Immediate versus early loading of immediately placed bone-level tapered dental implants with hydrophilic surface in full arch maxillary rehabilitation: A pilot randomized clinical trial with 2-year follow-up | Clinical Implant Dentistry and Related Research |
| #410 | Jihua Li | The accuracy of virtual surgical planning assisted management for L-shaped reduction malarplasty: A retrospective study | Journal of Cranio-Maxillo-Facial Surgery |
| #411 | Hatem Saifeldin | Assessing the immediate impact of Twin-block appliance insertion on adolescents' sleep using a wearable device | Orthodontics & Craniofacial Research |
| #412 | Liwen Zhang, Weiran Li | Alveolar bone remodelling and stability of mandibular incisors in adult patients after orthodontic treatment with premolar extractions: A prospective follow-up study | Orthodontics & Craniofacial Research |
| #414 | Sophie Kubon | Early detection of aseptic bone necrosis post-cranioplasty: A retrospective CT analysis using Hounsfield units | Journal of Cranio-Maxillo-Facial Surgery |
| #415 | Seung-Ha Park | Poly Lactic-co-Glycolic Acid Absorbable Plate Graft for Secondary Rhinoplasty in Asian Patients with Unilateral Cleft Lip Nose Deformity | The Cleft Palate Craniofacial Journal |
| #416 | Chunmiao Jiang | Efficacy of maxillary molar intrusion and quantification of related external apical root resorption - A comparison of two approaches | Orthodontics & Craniofacial Research |
| #417 | Sila Cagri Isler | Impact of peri-implant keratinized mucosa width on the longterm reconstructive outcomes of peri-implantitis: A retrospective analysis with a follow-up up to 10 years | Clinical Implant Dentistry and Related Research |
| #418 | Rubén Davó, Shengchi Fan | Long-term survival and complications of Quad Zygoma Protocol with Anatomy-Guided Approach in severely atrophic maxilla: A retrospective follow-up analysis of up to 17 years | Clinical Implant Dentistry and Related Research |
| #419 | Nikoo Bazsefidpay | Did temporomandibular gap arthroplasty with temporalis interpositional flap improve function and pain in patients with end-stage joint disease? A 5-year retrospective follow-up | Journal of Cranio-Maxillo-Facial Surgery |
| #420 | Behzad Salari | Investigating the effect of depression on clinical symptoms of temporomandibular disorder in young stressful men | Clinical and Experimental Dental Research |
| #421 | Kotaro Ito | Texture analysis using short‑tau inversion recovery magnetic resonance images to differentiate squamous cell carcinoma of the gingiva from medication‑related osteonecrosis of the jaw | Oral Radiology |
| #422 | Andrea Roccuzzo | Clinical, radiographic, and aesthetic outcomes at two narrowdiameter implants to replace congenital missing maxillary lateral incisors: A 3-year prospective, clinical study | Clinical Implant Dentistry and Related Research |
| #423 | Dong-Soon Choi | A comparison of maxillary posterior changes following facemask therapy: Skeletal anchorage versus tooth-borne anchorage | Orthodontics & Craniofacial Research |
| #424 | Hosein Eslami | Comparing the activity level of salivary matrix metalloproteinase‐8 in patients with diabetes and moderate to severe chronic generalized periodontitis | Clinical and Experimental Dental Research |
| #425 | Ville Myllymäki | Association of Periodontal Condition With Impaired Glucose Tolerance: Results of a 15‐Year Follow‐Up Study | Clinical and Experimental Dental Research |
| #426 | Katarzyna Kresse-Walczak | Evaluation of a protocol to assess a novel artificial biofilm equivalent for dentures—A prospective clinical pilot study | Gerodontology |
| #427 | Umberto Committeri | Epidemiological analysis of patients with isolated blowout fractures of orbital floor: Correlation between demographic characteristics and fracture area | Journal of Cranio-Maxillo-Facial Surgery |
| #430 | Christina Plikaitis | Efficacy of Demineralized Bone Matrix for Revision Alveolar Bone Grafting in Patients Previously Treated with Bone Morphogenetic Protein 2 (BMP-2) | The Cleft Palate Craniofacial Journal |
| #432 | Oleh Andrukhov | Changes in the alveolar bone morphology among different patterns of incisor inclination during the alignment phase in orthodontic treatment without premolar extraction | Orthodontics & Craniofacial Research |
| #433 | Ye Lin | Peri-zygomatic complications on zygomatic implants with or without penetrating the external surface of zygoma: A 2-year retrospective study | Clinical Implant Dentistry and Related Research |
| #434 | Chih-Hua Yeh | Temporomandibular joint degenerative changes following mandibular fracture: a computed tomography‑based study on the role of condylar involvement | Oral Radiology |
| #435 | Julia Naoumova | Salivary levels of Osteoprotegerin and receptor activator of nuclear factor-kappa ligand during orthodontic tooth movement—A prospective pilot study | Orthodontics & Craniofacial Research |
| #436 | Dan Grünfeld | Increased safety in periodontal surgery: Doppler ultrasound for detection of relevant palatal blood vessels—A proof-of-concept and cross-sectional study | Journal of Clinical Periodontology |
| #437 | Armin Khaleghi | Prevalence of Histopathologic Types of Gingival Lesions in the Iranian Population: A 22‐Year Retrospective Study | Clinical and Experimental Dental Research |
| #438 | Bing Han, Wei Liang, Si Chen | Alveolar bone morphology in patients with palatally-displaced maxillary lateral incisors before and after orthodontic treatment: A cone-beam computed tomography study | Orthodontics & Craniofacial Research |
| #440 | Ivy Trindade-Suedam | Screening for Obstructive Sleep Apnea and Associated Risk Factors in Adolescents and Adults With Isolated Robin Sequence | The Cleft Palate Craniofacial Journal |
| #441 | So Young Lim, Yong Oock Kim | Association of cranial base suture/synchondrosis fusion with severity of increased intracranial pressure in Crouzon syndrome | Journal of Cranio-Maxillo-Facial Surgery |
| #443 | Davide Bartolomeo Gissi | Direct healthcare costs of oral cancer: A retrospective study from a tertiary care center | Journal of Cranio-Maxillo-Facial Surgery |
| #444 | Robert M. Conville | Social, intellectual, psychological, and attractiveness judgements of lay people about patients before and after combined orthodontic-orthognathic surgical treatment | European Journal of Orthodontics |
| #445 | Jong Won Hong | A Retrospective study of anatomical differences in levator aponeurosis angle and length in East-Asian blepharoplasty | Journal of Cranio-Maxillo-Facial Surgery |
| #447 | Kenichi Kurita | Residual enamel removal to improve outcomes of mandibular third molar coronectomy: A single-center retrospective cohort study | Journal of Cranio-Maxillo-Facial Surgery |
| #448 | Wei Liu | Bone Density of the Condyle of Children with Craniofacial Microsomia and its Correlation with Condylar Resorption After Mandible Distraction Osteogenesis | The Cleft Palate Craniofacial Journal |
| #449 | Cunhui Fan, Chunmiao Jiang | Comparison of mandibular buccal shelf morphology between adolescents and adults with different vertical patterns using CBCT | Oral Radiology |
| #450 | Laura Anne Jacox | Influence of BMI percentile on craniofacial morphology and development in adolescents,Part II: elevated BMI is associated with larger final facial dimensions | European Journal of Orthodontics |
| #452 | Guoli Yang, Tingben Huang | Radiographic outcomes of lateral sinus floor elevation at sites without perforations and sites with perforations managed with a resorbable membrane: A retrospective study | Journal of Clinical Periodontology |
| #453 | Beau Meyer | Triazolam for Pediatric Dental Sedation: A Retrospective Evaluation of Safety and Changes in Visit Behavior | Pediatric Dentistry |
| #454 | Roberto L. Flores | Clinical Outcomes in Orthognathic Surgery for Craniofacial Microsomia Following Mandibular Distraction Using CBCT Analysis: A Retrospective Study | The Cleft Palate Craniofacial Journal |
| #455 | Jung-Seok Lee | Dimensional changes after horizontal and vertical guided bone regeneration without membrane fixation using the retentive flap technique: A 1-year retrospective study | Clinical Implant Dentistry and Related Research |
| #456 | Aljaž Golež | Effect of orthodontic space closure on dental pulp sensitivity. Prospective clinical trial | Orthodontics & Craniofacial Research |
| #457 | Jun Liu | The coordination of bimaxillary alveolar arch widths in subjects with normal occlusion or posterior crossbite: A CBCT retrospective study | Orthodontics & Craniofacial Research |
| #458 | Luis Ernesto Arriola-Guillén | Comparison of mesiodistal angulations of premolars and molars in anterior open bite subjects with different sagittal malocclusions: A retrospective study | International Orthodontics |
| #459 | Yoshinobu Yanagi | Imaging characteristics of incidentally detected cosmetic surgery‑derived foreign bodies on CT images in the maxillofacial region | Oral Radiology |
| #460 | Madhur Upadhyay | Mandibular molar protraction: A comparison between fixed functional appliances and temporary anchorage devices | Orthodontics & Craniofacial Research |
| #461 | Byung Jun Kim | Spontaneous recovery of underlying bony erosion following surgical removal of craniofacial dermoid cysts in periatric patients: A prospective study | Journal of Cranio-Maxillo-Facial Surgery |
| #463 | Valerie G. A. Suter | Incidental findings in cone beam computed tomography (CBCT) scans for implant treatment planning: a retrospective study of 404 CBCT scans | Oral Radiology |
| #465 | Yan Gu | Feasibility of spheno‐occipital synchondrosis fusion stages as an indicator for the assessment of maxillomandibular growth: A mixed longitudinal study | Orthodontics & Craniofacial Research |
| #467 | Damien Y. Brézulier | Bioglass 45S5, a relevant alternative to autogenous harvesting for secondary alveolar bone grafts in clefts? Retrospective study of one hundred surgeries | Journal of Cranio-Maxillo-Facial Surgery |
| #468 | Joon Soo Park | Exploring the usability of simulated patient methodology in dental clinics in Western Australia: A pilot survey | Clinical and Experimental Dental Research |
| #469 | Shubhangi Jain | Association of genetic polymorphism of interleukin 1-alpha and interleukin 1-beta with external apical root resorption in orthodontic patients | International Orthodontics |
| #470 | T. Xi | Does facial asymmetry vary between subjects of different age groups? A 3D stereophotogrammetry analysis | Journal of Cranio-Maxillo-Facial Surgery |
| #471 | Zhiyuan Zhang, Chi Yang, Guangzhou Xu | Long-term investigation of minimally invasive alcohol-based therapy as the treatment of odontogenic keratocyst: A retrospective cohort study | Journal of Cranio-Maxillo-Facial Surgery |
| #472 | Ellen Wen-Ching Ko | Three-dimensional facial soft-tissue changes after surgical orthodontics in different vertical facial types of skeletal Class III malocclusion: A retrospective study | Journal of Cranio-Maxillo-Facial Surgery |
| #474 | Emma Wigsten | General dental practitioners’ fees for root canal treatment, coronal restoration and follow‐on treatment in the adult population in Sweden: A 10‐year follow‐up of data from the Swedish Dental Register | Clinical and Experimental Dental Research |
| #476 | Lorenzo Franchi | Long‐term effects produced by early treatment of Class III malocclusion with rapid maxillary expansion and facemask followed by fixed appliances: A multicentre retro‐prospective controlled study | Orthodontics & Craniofacial Research |
| #477 | Alf Eliasson | Frequency of apical periodontitis in root‐filled teeth restored with post and core: a 5-year retrospective study | Clinical and Experimental Dental Research |
| #479 | Mert Calis | Comparison of closed percutaneous screw reduction versus open reduction and internal fixation in the treatment of frontal sinus fractures | Journal of Cranio-Maxillo-Facial Surgery |
| #480 | Tiago Fialho | Comparison of smile attractiveness in cases with gummy smile treated with botulinum toxin and maxillary impaction surgery | Journal of Cranio-Maxillo-Facial Surgery |
| #481 | Ching-Chih Lee | Incorporation of stromal tumor-infiltrating lymphocytes into an early death prediction model significantly improves net reclassification for outcome estimation in advanced buccal cancer | Journal of Cranio-Maxillo-Facial Surgery |
| #482 | Victor Yu | Comorbidity and Operative Time are Stronger Predictors than Age for Palatoplasty Adverse Airway Events, A NSQIP-P Study of 6668 Cases | The Cleft Palate Craniofacial Journal |
| #483 | Zuolin Jin, Qian Liu | A retrospective study of alveolar bone remodelling after anterior retraction in orthodontic tooth extraction cases with clear aligners and fixed appliances | Orthodontics & Craniofacial Research |
| #484 | Spyridon N. Papageorgiou | Long-term stability of curve of Spee depth among orthodontically treated patients | Orthodontics & Craniofacial Research |
| #486 | Shaobing Li, Jingyi Wu | The transalveolar approach with the small segmentation method for inclined maxillary sinus floor elevation | Clinical Implant Dentistry and Related Research |
| #487 | Tsun Man Choi | Automated three-dimensional analysis of facial asymmetry in patients with syndromic coronal synostosis | Journal of Cranio-Maxillo-Facial Surgery |
| #488 | Jihua Li | Does adjunctive fixation in conjunction with miniplate affect condylar position and morphology after mandibular advancement through bilateral sagittal split ramus osteotomy? A retrospective 3-dimensional CT comparative study | Journal of Cranio-Maxillo-Facial Surgery |
| #489 | Mahmoud F. Abu‑Ta’a | Maxillary sinuses, multiple aspects to consider prior to bone grafting procedure: a retrospective study in a sample of a Palestinian population | Oral Radiology |
| #490 | Frauke Beyling | Quality of occlusal outcome in adult Class II patients treated with completely customized lingual appliances and Class II elastics compared to adult Class I patients | European Journal of Orthodontics |
| #491 | Ahmet Turan Kaya | Relationship between maxillary sinus mucosal cyst and sinus ostium 2D area in three‑dimensional volumetric paranasal CT ımages | Oral Radiology |
| #492 | Sanaz Mihandoust | Prevalence of Middle Mesial Canal in Mandibular First Permanent Molars in a Persian Population: An In Vivo Cone‐Beam Computed Tomography Study | Clinical and Experimental Dental Research |
| #493 | Zhien Feng, Chong Wang | The impact of comorbidity on the diagnosis delay, treatment options and prognosis for advanced oral cancer: A retrospective result of the POROMS database | Journal of Cranio-Maxillo-Facial Surgery |
| #494 | John C. Schlotz | Longitudinal Follow-up Comparing Future Care of Primary Teeth Treated with Silver Diammine Fluoride: A Private Dental Claims Review | Pediatric Dentistry |
| #495 | Oyku Dalci | Dentoskeletal effects of mini-screw assisted, non-surgical palatal expansion in adults using a modified forcecontrolled polycyclic protocol: a single-centre retrospective study | European Journal of Orthodontics |
| #496 | Jadbinder Seehra | Impacted maxillary canine: Assessment of prevalence, severity and location of root resorption on maxillary incisors: A retrospective CBCT study | International Orthodontics |
| #497 | Shintaro Sukegawa | Risk factors for postoperative facial nerve injury in retromandibular-approach surgery: A retrospective study including CT measurements of maxillofacial bone structure | Journal of Cranio-Maxillo-Facial Surgery |
| #498 | Courtney Carpenter | Endoscopic Strip Craniectomy and Helmet Therapy for Sagittal Craniosynostosis: An Analysis of Cranial Growth Changes in the Early Postoperative Period | The Cleft Palate Craniofacial Journal |
| #501 | Miren de las Fuentes Monreal | Skeletal surgical approach in Down Syndrome with Obstructive Sleep Apnea | Journal of Cranio-Maxillo-Facial Surgery |
| #502 | Serhat Şibar | Evaluation of Orbitomalar Region Projection in Patients With Operated Cleft Lip and Palate (Cephalometric Study) | The Cleft Palate Craniofacial Journal |
| #503 | Gesa M. Richter | Oral microbiota of patients with phenylketonuria: A nation-based cross-sectional study | Journal of Clinical Periodontology |
| #504 | Stephane P. Kerner | Reduced bone dimension in patients affected by oligodontia: A retrospective study on maxillary and mandibular CBCT | Journal of Clinical Periodontology |
| #505 | María Baus-Domínguez, María-Ángeles Serrera-Figallo | Prospective observational cohort study of the change of the marginal bone crest in relation to the prosthetic abutment height and the peri-implant vertical mucosal thickness at implants positioned subcrestally | Clinical Implant Dentistry and Related Research |
| #506 | Eglė Zasčiurinskienė | Malocclusions, pathologic tooth migration, and the need for orthodontic treatment in subjects with stage III–IV periodontitis. A cross-sectional study | European Journal of Orthodontics |
| #507 | Anwar B. Bataineh | The incidence and patterns of maxillofacial fractures and associated head and neck injuries | Journal of Cranio-Maxillo-Facial Surgery |
| #509 | Ki Beom Kim | Relapse and failure rates between CAD/CAM and conventional fixed retainers: a 2-year follow-up of a randomized controlled clinical trial | European Journal of Orthodontics |
| #510 | Diandra S. Natsir Kalla | Postoperative Daycare as a Safe and Cost-Effective Option for Secondary Alveolar Bone Graft (SABG) Surgery: A Retrospective Comparative Cohort Study | The Cleft Palate Craniofacial Journal |
| #511 | Thomas J. Sitzman | Protocol for a Prospective Observational Study of Revision Palatoplasty Versus Pharyngoplasty for Treatment of Velopharyngeal Insufficiency Following Cleft Palate Repair | The Cleft Palate Craniofacial Journal |
| #512 | José Nart | Influence of keratinized mucosa width on the resolution of peri‐implant mucositis: A prospective cohort study | Clinical Implant Dentistry and Related Research |
| #513 | Alec Scott Griffin | Dental implant failures in Utah and US veteran cohorts | Clinical Implant Dentistry and Related Research |
| #514 | Hojin Park | Vascular variation of temporoparietal fascia in microtia associated with hemifacial microsomia | Journal of Cranio-Maxillo-Facial Surgery |
| #515 | Chi Yang, Minjie Chen, Hui Li | Feasibility of simultaneous TMJ arthroscopy in ADDwoR patients undergoing orthognathic surgery | Journal of Cranio-Maxillo-Facial Surgery |
| #516 | Y. Gugliotta | A retrospective analysis of the management and surgical treatment of orbital lesions: outcomes and rationale | Journal of Cranio-Maxillo-Facial Surgery |
| #518 | Massimo Del Fabbro | Mucosal cyst aspiration in conjunction with maxillary sinus elevation: A clinical cohort study | Clinical Implant Dentistry and Related Research |
| #519 | Ezgi Sunal Akturk | Evaluation of Anxiety in Turkish Parents of Newborns with Cleft Palate with or Without Cleft Lip | The Cleft Palate Craniofacial Journal |
| #520 | Elizabeth West Ellis | An Enhanced Audiologic Protocol for Early Identification of Conductive Hearing Loss in Patients with Cleft Palate | The Cleft Palate Craniofacial Journal |
| #521 | Wei Liu | 3D-CT measurements of facial symmetry in severe CFM patients: A comparative study between mandibular ascending ramus distraction osteogenesis and bone grafting | Journal of Cranio-Maxillo-Facial Surgery |
| #522 | Elbe Peter | Does orthodontic treatment improve the Oral Health- Related Quality of Life when assessed using the Malocclusion Impact Questionnaire—a 3-year prospective longitudinal cohort study | European Journal of Orthodontics |
| #523 | Ambika Chadha | The Implications of Laterality in Unilateral Cleft Lip Reconstruction: A Global Survey of Cleft Surgeons | The Cleft Palate Craniofacial Journal |
| #524 | Basel El-Sabbagh | 3D evaluation of sagittal inclination of the maxillary dentition in relation to facial landmarks: A cohort study | Clinical Implant Dentistry and Related Research |
| #525 | Justine C. Lee | Association of Patient-Reported Anxiety and Pain After Alveolar Bone Grafting | The Cleft Palate Craniofacial Journal |
| #526 | Teagan Fink | The Early Operative Burden for Children Born with Cleft Lip and Palate | The Cleft Palate Craniofacial Journal |
| #527 | Phayvanh P. Pecha | Impact of Syndromes on Sleep-Disordered Breathing in Children After Cleft Palate Repair | The Cleft Palate Craniofacial Journal |
| #528 | Lakshmi Kollara | Assessment of the Velopharyngeal Mechanism at Rest and During Speech in Children With 22q11.2DS: A Cross-Sectional Study | The Cleft Palate Craniofacial Journal |
| #529 | Sebastian Espinosa | Single photon emission computed tomography (SPECT) diagnostic accuracy in active unilateral condylar hyperplasia: Retrospective study | Journal of Cranio-Maxillo-Facial Surgery |
| #530 | Ni Zeng | Proposed clinical model for predicting speech outcomes in patients undergoing Furlow palatoplasty for velopharyngeal insufficiency after primary palatoplasty | Journal of Cranio-Maxillo-Facial Surgery |
| #531 | Caroline M. Sawicki | Perioperative Information Needs of Parents of Patients With Special Health Care Needs: A Cross-Sectional Study | Pediatric Dentistry |
| #532 | Alessandro Pozzi | Accuracy of navigation guided implant surgery for immediate loading complete arch restorations: Prospective clinical trial | Clinical Implant Dentistry and Related Research |
| #533 | Sarah Pak | A 3-Dimensional Evaluation of the Effects of Unilateral Vertical Mandibular Distraction Osteogenesis on Airway Volume Among Patients With Hemifacial Microsomia | The Cleft Palate Craniofacial Journal |
| #534 | Chihiro Tanikawa | Prognostic Factors for Orthognathic Surgery in Children With Cleft Lip and/or Palate: Dentition and Palatal Morphology | The Cleft Palate-Craniofacial Journal |
| #535 | Giuseppe Consorti | Customized orbital implant versus 3D preformed titanium mesh for orbital fracture repair: A retrospective comparative analysis of orbital reconstruction accuracy | Journal of Cranio-Maxillo-Facial Surgery |
| #536 | Kamil Nelke | A proposed protocol for correlation between bone density in hemimandibular hyperplasia radiography and histopathological findings — A retrospective study | Journal of Cranio-Maxillo-Facial Surgery |
| #538 | Feng Wang, Yiqun Wu | Longitudinal reactions of maxillary sinus in patients treated with multiple zygomatic implants: A modified radiographic evaluation with clinical follow-up | Clinical Implant Dentistry and Related Research |
| #539 | Tiffanie-Marie Borg | Surgical Management of Submucous Cleft Palate by Radical Muscle Dissection Veloplasty: Speech Outcomes in Patients with 22q11.2 Deletion Syndrome | The Cleft Palate Craniofacial Journal |
| #540 | Sanna Syrjäläinen | Dietary inflammatory index in relation to salivary cytokine concentrations and periodontitis: A cross-sectional analysis | Journal of Clinical Periodontology |
| #541 | Bettina Dannewitz | Decision-making on systemic antibiotics in the management of periodontitis: A retrospective comparison of two concepts | Journal of Clinical Periodontology |
| #542 | Nobuyuki Mitsukawa | Changes in mandibular position during midface distraction in patients with syndromic craniosynostosis | Journal of Cranio-Maxillo-Facial Surgery |
| #543 | Yi Man | Effectiveness and complications of transcrestal sinus floor elevation using the cushioned grind-out technique: A retrospective cohort study with up to 7 years of follow-up | Journal of Clinical Periodontology |
| #544 | Erik M. Wolfswinkel | American Indian and Alaska Native Accessibility to Comprehensive Cleft Lip and Palate Treatment | The Cleft Palate Craniofacial Journal |
| #545 | Franz J. Strauss | Impact of keratinized mucosa on implant‐health related parameters: A 10‐year prospective re‐analysis study | Clinical Implant Dentistry and Related Research |
| #546 | Natalia I. Chalmers | Trends of Pediatric Dental Rehabilitation Using General Anesthesia by Service Location During the COVID-19 Public Health Emergency, 2019 to 2021 | Pediatric Dentistry |
| #547 | S. R. Baker | Development and psychometric validation of the gum health experience questionnaire | Journal of Clinical Periodontology |
| #549 | Michael Blumer | Surgical treatment of nasal fractures may benefit from intraoperative 3D imaging | Journal of Cranio-Maxillo-Facial Surgery |
| #550 | Li Huang | Modified patient-specific surgical-guide-assisted precise treatment of unilateral comminuted zygomaticomaxillary complex fractures: A 5-year retrospective study | Journal of Cranio-Maxillo-Facial Surgery |
| #551 | Adrian Franke | A single-centre retrospective 10-year experience of the rhombic 3D condylar fracture plate for open reduction and internal fixation of condylar neck and base fractures | Journal of Cranio-Maxillo-Facial Surgery |
| #552 | Zhenmin Zhao | Objective and subjective evaluation of the efficacy of trans-sutural distraction osteogenesis: A correlation analysis between computed tomography measurements and FACE-Q scores | Journal of Cranio-Maxillo-Facial Surgery |
| #553 | Evie Stergiakouli | Prevalence and Factors Associated with Behavioral Problems in 5-Year-Old Children Born with Cleft Lip and/or Palate from the Cleft Collective | The Cleft Palate Craniofacial Journal |
| #554 | Marie A. Cornelis | Stability, survival, patient satisfaction, and costminimization of CAD/CAM versus conventional multistranded fixed retainers in orthodontic patients: a 2-year follow-up of a two-centre randomized controlled trial | European Journal of Orthodontics |
| #555 | Koichiro Ueki | Maxillary bone healing and CT value after Le Fort I osteotomy using absorbable plate system: A retrospective study | Journal of Cranio-Maxillo-Facial Surgery |
| #556 | Brianne B. Roby | The Incidence of Velopharyngeal Insufficiency in Stickler Syndrome | The Cleft Palate Craniofacial Journal |
| #557 | Theodore Eliades, Sara Eslami | Effects of intraoral aging on mechanical properties of directly printed aligners vs. thermoformed aligners: an in vivo prospective investigation | European Journal of Orthodontics |
| #558 | Yan Yu Li | Prenatal Diagnosis and Pregnancy Outcomes of Fetuses With Orofacial Cleft: A Retrospective Cohort Study in Two Centres in Hong Kong | The Cleft Palate Craniofacial Journal |
| #559 | Matti Hannula | Effects of headgear timing on dental arch changes from 7 to 18 years of age: a follow-up study | European Journal of Orthodontics |
| #560 | Jorge Gamonal Aravena, Franz Josef Strauss | Epidemiology of mid-buccal gingival recessions according to the 2018 Classification System in South America: Results from two population-based studies | Journal of Clinical Periodontology |
| #561 | Jesse A. Taylor | Mandibular condyle volumes are associated with facial asymmetry in patients with cleft lip and palate: A retrospective cohort study | Journal of Cranio-Maxillo-Facial Surgery |
| #563 | *Huanxing Meng, Zhenyu Zhang* | Long-term exposure to ambient fine particulate matter and periodontitis: An observational study using nationally representative survey data | Journal of Clinical Periodontology |
| #564 | Aysegul Sari | Periodontal conditions and association of periodontitis with oral-health-related quality of life in patients experiencing different episodes of bipolar disorder compared with healthy controls | Journal of Clinical Periodontology |
| #565 | Fuming He, Antian Xu | Clinical outcomes and risk factor analysis of dental implants inserted with lateral maxillary sinus floor augmentation: A 3- to 8-year retrospective study | Journal of Clinical Periodontology |
| #566 | Ya-Jun Chen | Contribution of individual and cumulative social determinants of health underlying gender disparities in periodontitis in a representative US population: A cross-sectional NHANES study | Journal of Clinical Periodontology |
| #567 | Niko Christian Bock | Adult Herbst-multibracket appliance treatment—how stable are the results very long term? | European Journal of Orthodontics |
| #569 | Mark A. Green | Prognosis of Maxillary Central Incisors in Patients with Bilateral Cleft Lip/Palate | The Cleft Palate Craniofacial Journal |
| #570 | Mathias Lemberger | Validation and comparison of 2D grading scales and 3D volumetric measurements for outcome assessment of bone-grafted alveolar clefts in children | European Journal of Orthodontics |
| #571 | Miriam Shalish | Orthodontic Treatment of Patients With Clefts: Satisfaction and Psychological Aspects | The Cleft Palate Craniofacial Journal |
| #572 | Masanori Iwasaki | Serum levels of vitamin D and periodontal inflammation in community‐dwelling older Japanese adults: The Otassha Study | Journal of Clinical Periodontology |
| #573 | Hiroshi Kamioka | Comprehensive clinical evaluation of indirect and direct bonding techniques in orthodontic treatment: a singlecentre, open-label, quasi-randomized controlled clinical trial | European Journal of Orthodontics |
| #574 | Farhan Bazargani | Three-dimensional comparison of tooth-borne and toothbone- borne RME appliances: a randomized controlled trial with 5-year follow-up | European Journal of Orthodontics |
| #575 | David P Rice | The effect of early childhood non-nutritive sucking behavior including pacifiers on malocclusion: a randomized controlled trial | European Journal of Orthodontics |
| #576 | Hongchang Lai, Junyu Shi | Association between masticatory function and depression in older adults: Results from NHANES 2009 to 2018 | Journal of Clinical Periodontology |
| #578 | Jean Calvo | Dental desensitization to increase comfort with preventive dental visits for children with autism spectrum disorder | Pediatric Dentistry |
| #579 | Demetrios Halazonetis | Cervical headgear effectiveness in distalizing molars in relation to patient compliance | European Journal of Orthodontics |
| #580 | Beatriz Quevedo | Mandibular second premolar distoangulation: a longitudinal follow-up from the mixed to the permanent dentition | European Journal of Orthodontics |
| #581 | Georgios Kanavakis | Palatal canine impaction is associated with craniofacial shape in humans | European Journal of Orthodontics |
| #582 | Ama Johal | The skeletal and dental effects of Hanks Herbst versus twin block appliances for class II correction in growing patients: a randomized clinical trial | European Journal of Orthodontics |
| #583 | Masanori Iwasaki | The association between trypsin‐like protease activity in the oral cavity and kidney function in Japanese workers | Journal of Clinical Periodontology |
| #584 | Jian Li | Comprehensive three-dimensional cone beam computed tomography assessment unilateral alveolar cleft reconstruction using autologous iliac cancellous bone combined with deproteinized bovine bone: A clinical retrospective evaluation | Journal of Cranio-Maxillo-Facial Surgery |
| #585 | Jie Long | Assessment of the relationship between the three-dimensional precise location of the mandibular third molar and the volume ratio of the impacted mandibular third molar to the mandibular angle, and the patterns of mandibular angle fracture: A retrospective study | Journal of Cranio-Maxillo-Facial Surgery |
| #586 | M S Muthu | Variations in Primary Molar Contact and Approximal Caries in Children: A Three-Year Prospective Cohort Study | Pediatric Dentistry |
| #587 | Renato Corrêa Viana Casarin | Parents with periodontitis drive the early acquisition of dysbiotic microbiomes in their offspring | Journal of Clinical Periodontology |
| #588 | M.S. Muthu | Marginal Gingival Thickness Assessment in Three- To Six-Year-Old Preschool Children | Pediatric Dentistry |
| #589 | Celia E. Heppner | Multisite Study Investigating Child and Parent Proxy Reported Quality of Life in Children With Cleft Lip and/or Palate | The Cleft Palate Craniofacial Journal |
| #590 | Carrie L. Heike | Observations by Caregivers Using the Infant with Clefts Observation Outcomes Instrument (iCOO): A Comparison of Three Versus Seven-day Daily Diaries | The Cleft Palate Craniofacial Journal |
| #591 | Guy Willems | Early prevention of maxillary canine impaction: A Randomaized clinical trial | European Journal of Orthodontics |
| #592 | Cory M. Resnick | The Family Impact of a Robin Sequence Prenatal Diagnosis | The Cleft Palate Craniofacial Journal |
| #593 | Chihiro Tanikawa | Relationship Between Stigma Experience and Self-Perception Related to Facial Appearance in Young Japanese Patients with Cleft lip and/or Palate | The Cleft Palate Craniofacial Journal |
| #594 | Hamit Tunç | Comparison of the Paranasal Sinus Features of Paediatric Patients with and Without Cleft Palate: A CBCT study | The Cleft Palate Craniofacial Journal |
| #595 | Małgorzata Peruga | Correlation of sex hormone levels with orthodontic tooth movement in the maxilla: a prospective cohort study | European Journal of Orthodontics |
| #596 | Azza Tagelsir Ahmed | Early Lead Exposure Associated with Molar Hypomineralization | Pediatric Dentistry |
| #597 | Rod W. Hunt | Intellectual Functioning of Children With Isolated PRS, PRS-Plus, and Syndromic PRS | The Cleft Palate Craniofacial Journal |
| #598 | Branislav V. Bajkin | Risk of dentoalveolar surgery postoperative bleeding in patients taking direct oral anticoagulants and vitamin K antagonists: A prospective observational study | Journal of Cranio-Maxillo-Facial Surgery |
| #599 | Nina Ellefsen Lindberg | Early Follow-up of Parents by a Specialized Cleft Nurse After the Birth of an Infant with Cleft lip and/or Palate | The Cleft Palate Craniofacial Journal |
| #600 | Julia Naoumova | Longitudinal study of root resorption on incisors caused by impacted maxillary canines—a clinical and cone beam CT assessment | European Journal of Orthodontics |
| #601 | Fábio Renato Manzolli Leite | Sugar-sweetened beverage consumption and periodontitis among adults: A population-based cross-sectional study | Journal of Clinical Periodontology |
| #602 | Alessandra Areas e Souza | Occurrence of periodontal diseases according to the ACES 2018 Classification Framework and the CDC/AAP definition:A cross-sectional study in a major Brazilian city | Journal of Clinical Periodontology |
| #603 | *Kamlesh B Patel* | Effect of Veau Class on Levator Veli Palatini Muscle Composition | The Cleft Palate-Craniofacial Journal |
| #604 | *Zhong-Lin Jia, Bing Shi* | Integrated Analysis of the Association Between Variants at PAX7 and NSCL/P in the Han Population | The Cleft Palate Craniofacial Journal |
| #605 | Peter Stoustrup | Will supplemental cone beam computed tomography change the treatment plan of impacted maxillary canines based on 2D radiography? A prospective clinical study | European Journal of Orthodontics |
| #606 | Mary Hardin-Jones | Stop Consonant Production in Children with Cleft Palate After Palatoplasty | The Cleft Palate Craniofacial Journal |
| #607 | Lang Liang | Association of the Life's Essential 8 cardiovascular health score with periodontitis among US adults | Journal of Clinical Periodontology |
| #608 | Xing Wang | Extraction of high inverted mesiodentes via the labial, palatal and subperiostal intranasal approach: A clinical prospective study | Journal of Cranio-Maxillo-Facial Surgery |
| #609 | Prabhat Kumar Chaudhari | Effects of RME on Hearing in UCLP Patients: A Pilot Study | The Cleft Palate Craniofacial Journal |
| #610 | Tim Bressmann | The Impact of Fan-Type Rapid Palatal Expanders on Speech in Patients With Unilateral Cleft Lip and Palate | The Cleft Palate Craniofacial Journal |
| #611 | Katelyn J. Kotlarek | Current Practice Patterns and Training Pathways for Feeding Infants with Cleft Palate | The Cleft Palate Craniofacial Journal |
| #612 | Elizabeth B. Card | Impact of Illustrated Postoperative Instructions on Knowledge and Retention During a Cleft Lip and Palate Surgical Mission | The Cleft Palate Craniofacial Journal |
| #613 | Tymon Skadorwa | The validation of morphometric outcomes and stratification system for nonsyndromic sagittal craniosynostosis following total calvarial remodeling | Journal of Cranio-Maxillo-Facial Surgery |
| #614 | Adaia Valls-Ontañón | Effectiveness of virtual reality in relieving anxiety and controlling hemodynamics during oral surgery under local anesthesia: A prospective randomized comparative study | Journal of Cranio-Maxillo-Facial Surgery |
| #615 | Claudia Resende Leal | Evaluation of Radiographic Outcomes of Alveolar Graft Associated with Premaxillary Osteotomy Performed with rhBMP-2 | The Cleft Palate Craniofacial Journal |
| #616 | William P. Magee III | Premaxillary Setback in Bilateral Cleft Lip and Palate Repair | The Cleft Palate Craniofacial Journal |
| #617 | Maurizio S. Tonetti, Changzheng Yuan | The potential causal path between periodontitis stage diagnosis and vegetable consumption is mediated by loss of posterior functional tooth units and masticatory function | Journal of Clinical Periodontology |
| #618 | Yasuaki Saijo | Null Association Between Isolated Orofacial Clefts and Sleep Duration: A Cohort Study From the Japan Environment and Children's Study | The Cleft Palate Craniofacial Journal |
| #619 | Mathias Lemberger | Long-term radiographic and periodontal evaluations of the bone-grafted alveolar cleft region in young adults born with a UCLP | European Journal of Orthodontics |
| #620 | Hidetaka Miyashita | Factors affecting the quality of life of patients with medication-related osteonecrosis of the jaw during treatment: A quality-of-life survey and causal analysis | Journal of Cranio-Maxillo-Facial Surgery |
| #621 | Renato Assis Machado | Ethnic Differences in the Brazilian Population Influence the Impact of BMP4 Genetic Variants on Susceptibility of Nonsyndromic Orofacial Clefts | The Cleft Palate Craniofacial Journal |
| #622 | Mahvash Hasani | Comparison of Superior Semicircular Canal Bone Thickness and Patterns in Unilateral and Bilateral Cleft Patients and Normal Controls: A CBCT Study | The Cleft Palate Craniofacial Journal |
| #623 | Sila Cagri Isler | Soft‐Tissue Phenotype as a Risk Indicator of Peri‐Implantitis and Peri‐Implant Soft‐Tissue Dehiscence—A Cross‐Sectional Study | Journal of Clinical Periodontology |
| #624 | Olivier Huck | Influence of systemic sclerosis on periodontal health: A case–control study | Journal of Clinical Periodontology |
| #625 | *Kaitlyn M. Fladeboe, Joyce Yi-Frazier,* | Feasibility and Acceptability of the Promoting Resilience in Stress Management-Parent (PRISM-P) Intervention for Caregivers of Children with Craniofacial Conditions | The Cleft Palate Craniofacial Journal |
| #626 | Koji Mizutani | Improvement of periodontal parameters following intensive diabetes care and supragingival dental prophylaxis in patients with type 2 diabetes: A prospective cohort study | Journal of Clinical Periodontology |
| #627 | Chon T. Ho Nguyen | Three-Dimensional Evaluation of Nasolabial Morphology After Applying Presurgical Nasoalveolar Molding in Primary Unilateral Cheiloplasty Using the Modified Millard Technique | The Cleft Palate Craniofacial Journal |
| #628 | Anton Straub | Investigation of clindamycin concentrations in human plasma and jawbone tissue in patients with osteonecrosis of the jaw: A prospective trial | Journal of Cranio-Maxillo-Facial Surgery |
| #629 | CM Rivers | Philtral Height Discrepancy in Patients with Complete and Incomplete Cleft Lips + /-Palate – Results from a Single Nation Consecutive Cohort | The Cleft Palate Craniofacial Journal |
| #630 | A. Valls-Ontañón | Le fort I osteotomy with or without concomitant removal of upper third molars: A prospective cohort study of intraoperative findings, related complications, and level of pterygomaxillary separation after down-fracture | Journal of Cranio-Maxillo-Facial Surgery |
| #631 | Pascale Gavelle | Parental and Child Diagnosis Storytelling and Self-Image in French Children With Cleft lip With or Without Cleft Palate | The Cleft Palate Craniofacial Journal |
| #632 | Aya A. Mitani | Modelling time‐varying risk factors of tooth loss: Results from joint model compared with extended Cox regression model | Journal of Clinical Periodontology |
| #633 | Max-Philipp Lentzen | Oral health-related quality of life (OHRQoL) and implant therapy: A prospective multicenter study of preoperative, intermediate, and posttreatment assessment | Journal of Cranio-Maxillo-Facial Surgery |
| #634 | Reza Tabrizi | Which factors affect the risk of membrane perforation in lateral window maxillary sinus elevation? A prospective cohort study | Journal of Cranio-Maxillo-Facial Surgery |
| #635 | Koichiro Ueki | Bone healing and stability after advancement genioplasty using a pre-bent absorbable plate and screws | Journal of Cranio-Maxillo-Facial Surgery |
| #636 | Balamanikandasrinivasan Chandrasekaran | Comparison of accuracy of immediate implant placement between planned and actual position in pre-extractive versus conventional method - A prospective study | Journal of Cranio-Maxillo-Facial Surgery |
| #637 | Izumi Yoshioka | Horseshoe osteotomy maintains the nasal cavity and function after superior repositioning | Journal of Cranio-Maxillo-Facial Surgery |
| #638 | Midia Najar Chalien | Long-term Outcome for Two-Stage Palatal Closure With Different Timings for Hard Palate Surgery: Craniofacial Growth and Dental Arch Relation | The Cleft Palate Craniofacial Journal |
| #639 | Azeez Butali | Damaging Mutations in AFDN Contribute to Risk of Nonsyndromic Cleft Lip With or Without Cleft Palate | The Cleft Palate Craniofacial Journal |
| #640 | David Faustino Ângelo | Effectiveness of double-puncture temporomandibular joint arthrocentesis with viscosupplementation in different categories of severity – a prospective study | Journal of Cranio-Maxillo-Facial Surgery |
| #641 | Adriana Agell Sogbe | Comparative Study of Maxillary Growth in Patients With Unilateral Cleft Treated With and Without Pre-Surgical Orthopedics | The Cleft Palate Craniofacial Journal |
| #642 | Harriet Larvin | All-cause and cause-specific mortality in US adults with periodontal diseases: A prospective cohort study | Journal of Clinical Periodontology |
| #643 | Wei Hui Chen | Comparison of two preserved cartilage iliac crest cortical-cancellous bone blocks graft harvesting techniques in children: A prospective, double-blind, randomized clinical trial | Journal of Cranio-Maxillo-Facial Surgery |
| #644 | *Birte Holtfreter* | Serum lipoprotein subfractions are associated with the periodontal status: Results from the population-based cohort SHIP-TREND | Journal of Cranio-Maxillo-Facial Surgery |
| #645 | Crystal Marruganti | Healthy lifestyles are associated with a better response to periodontal therapy: A prospective cohort study | Journal of Clinical Periodontology |
| #646 | *Bing Shi, Zhonglin Jia* | MMP16 as NSCL ± P Susceptible Gene in Western Han Chinese | The Cleft Palate-Craniofacial Journal |
| #647 | Thomas Starch-Jensen | Patient’s perception of recovery following surgical removal of mandibular third molars: A prospective European multi-center study | Journal of Cranio-Maxillo-Facial Surgery |
| #648 | Niamh Coffey | Periodontal disease prevalence and oral hygiene status of adults with cystic fibrosis: A case–control study | Journal of Clinical Periodontology |
| #649 | Kitti Torrungruang | Periodontitis and hypertension are linked through systemic inflammation: A 5‐year longitudinal study | Journal of Clinical Periodontology |
| #651 | Sandra L. Dimenäs | Adolescents' self-reported experiences following a person‐centred, theory‐based educational intervention versus conventional education for improved oral hygiene: Analysis of secondary outcomes of a randomized field study | Journal of Clinical Periodontology |
| #653 | Cheng-I Yen | A prospective study of psychological adjustment during and after forehead flap nasal reconstruction | Journal of Cranio-Maxillo-Facial Surgery |
| #654 | Daniel S. Thoma | Randomized controlled multi‐centre study comparing shorter dental implants (6 mm) to longer dental implants (11–15 mm) in combination with sinus floor elevation procedures: 10‐year data | Journal of Clinical Periodontology |
| #655 | Alejandro Piza | Long-term mandibular growth in patients with airway obstruction treated with mandibular distraction | Journal of Cranio-Maxillo-Facial Surgery |
| #656 | Hao Xu | Periodontitis and the risk of all‐cause and cause‐specific mortality among US adults with diabetes: A population‐based cohort study | Journal of Clinical Periodontology |
| #657 | Elise G. Zuiderveld | Single immediate implant placement in the maxillary aesthetic zone with and without connective tissue grafting: Results of a 5‐year randomized controlled trial | Journal of Clinical Periodontology |
| #658 | Sašo Ivanovski, Pingping Han | Effect of non‐surgical periodontal therapy on salivary histone deacetylases expression: A prospective clinical study | Journal of Clinical Periodontology |
| #659 | Minkook Son | Interrupted time series analysis of chronic periodontitis‐related procedures before and after the scaling reimbursement policy in Korea | Journal of Clinical Periodontology |
| #660 | Maria Clotilde Carra | Prevalence of self‐reported severe periodontitis: Data from the population‐based CONSTANCES cohort | Journal of Clinical Periodontology |
| #661 | Yanmei Chen, Jianping Bin | Association of Oral Microbiome Diversity and All‐Cause Mortality in the General US Population and in Individuals With Chronic Diseases: A Prospective Cohort Study | Journal of Clinical Periodontology |
| #662 | Michiko Furuta | Longitudinal association between periodontal condition and glycaemic status in middle‐aged adults: A cross‐lagged panel analysis | Journal of Clinical Periodontology |
| #663 | Michael Glogauer | Utilizing Oral Neutrophil Counts as an Indicator of Oral Inflammation Associated With Periodontal Disease: A Blinded Multicentre Study | Journal of Clinical Periodontology |
| #664 | Andrea Kjellström Wagner | Treated periodontitis and recurrent events after first‐time myocardial infarction: A Danish nationwide cohort study | Journal of Clinical Periodontology |
| #665 | Birte Holtfreter | Interdental cleaning aids are beneficial for oral health at 7‐year follow‐up: Results from the Study of Health in Pomerania (SHIP‐TREND) | Journal of Clinical Periodontology |
| #666 | Giuseppe Alexandre Romito | Clinical outcomes following atrophic alveolar ridge reconstruction using collagenated xenogeneic bone block or autogenous bone block: One-year follow-up of a randomized controlled clinical | Journal of Clinical Periodontology |
| #667 | Luigi Nibali | Minimally invasive non‐surgical periodontal therapy of intrabony defects: A prospective multi‐centre cohort study | Journal of Clinical Periodontology |
| #668 | Min Zhou | Elevated neutrophil extracellular trap levels in periodontitis: Implications for keratinization and barrier function in gingival epithelium | Journal of Clinical Periodontology |
| #669 | Yago Leira, Tomás Sobrino | Association of periodontitis with cognitive decline and its progression: Contribution of blood‐based biomarkers of Alzheimer's disease to this relationship | Journal of Clinical Periodontology |
| #670 | Peppi Koivunen, Pekka Ylöstalo | Higher haemoglobin levels are associated with impaired periodontal status | Journal of Clinical Periodontology |
| #671 | Anthony de Buys Roessingh | Bullying in Swiss Youth Born with a Unilateral Cleft lip and Palate by Self- and Parent-Report | The Cleft Palate Craniofacial Journal |
| #672 | Yoshihiro Shimazaki | Periodontitis and the incidence of metabolic syndrome: An 8‐year longitudinal study of an adult Japanese cohort | Journal of Clinical Periodontology |
| #673 | Jan Cosyn | A Randomized Controlled Trial on the Timing of Soft‐Tissue Augmentation in Immediate Implant Placement: Hard‐Tissue Changes and Clinical Outcome | Journal of Clinical Periodontology |
| #674 | Caroline Riben Grundström | Systemic antibiotics in the surgical treatment of peri‐implantitis: A randomized placebo‐controlled trial | Journal of Clinical Periodontology |
| E001 | Shinji Matsuda | Predictive factors of periodontal regeneration outcomes using rhFGF‐2: A case–control study | Journal of Periodontal Research |
| E002 | Canan Önder | Does smoking influence tryptophan metabolism in periodontal inflammation? A cross‐sectional study | Journal of Periodontal Research |
| E003 | Fa Chen, Jing Wang | Association of individual and combined exposures of 10 metals with periodontitis: Results from a large population‐based study | Journal of Periodontal Research |
| E004 | MS Hopcraft | Mental health, psychological distress and burnout in Australian dental practitioners | Australian Dental Journal |
| E005 | Emilija D. Jensen | The role of hyperbaric oxygen in osteoradionecrosis—a prophylactic insight | Australian Dental Journal |
| E006 | Angelika Silbereisen | Value of gingival crevicular fluid TREM‐1, PGLYRP1, and IL‐1β levels during menopause | Journal of Periodontal Research |
| E007 | Feifei Niu | Hormone replacement therapy and periodontitis progression in postmenopausal women: A prospective cohort study | Journal of Periodontal Research |
| E008 | Peter Parashos | Prevalence and morphology of different root canal systems in mandibular premolars: a cross-sectional observational study | Australian Dental Journal |
| E009 | H. Yemenoglu | The effect of interleukin‐20 on periodontal tissue destruction in individuals with periodontitis | Journal of Periodontal Research |
| E010 | Eryi Lu, Min Jin, Yuhua Gong | Differential expression of FSTL1 and its correlation with the pathological process of periodontitis | Journal of Periodontal Research |
| E011 | H. Tepe | Clinical performance of non-carious cervical lesions restored with using self-etch mode of adhesives according to the FDI criteria | Australian Dental Journal |
| E012 | K Davies | Dental referrals for patients with diabetes: survey of barriers and enablers for medical and health professionals | Australian Dental Journal |
| E013 | Sema Nur Sevinç Gül | Serum asprosin levels are increased in patients with periodontitis and ST‐segment elevation myocardial infarction and correlated with periodontal parameters: A case–control study | Journal of Periodontal Research |
| E014 | Danijela Gasevic | Self‐reported oral health status, edentulism and all‐cause mortality risk in 12 809 Australian older adults: a prospective cohort study | Australian Dental Journal |
| E015 | Luigi Nibali | Association between defect morphology and healing of intrabony defects treated with minimally invasive non‐surgical therapy: A pilot exploratory analysis of two cohorts | Journal of Periodontal Research |
| E016 | Alberto Monje | Disease recurrence during supportive therapy following peri‐implantitis treatment: A retrospective study | Journal of Periodontal Research |
| E017 | Jilen Patel | Survival of pre‐formed zirconia crowns in primary teeth: a prospective practice‐based cohort study | Australian Dental Journal |
| E018 | Sandra March | Positive impacts of oral health services provision by a student‐led primary care clinic to an Australian rural indigenous community | Australian Dental Journal |
| E019 | Wai Keung Leung | Assessing oral health and the minimally important differences in oral health‐related quality of life of non‐diabetic and diabetic patients: a cross‐sectional study | Australian Dental Journal |
| E020 | Ju Sun Heo, Ki Hoon Ahn | Differences in maternal subgingival microbiome between preterm and term births: The MOHEPI study | Journal of Periodontal Research |
| E022 | Ruifang Lu, Huanxin Meng | The long‐term effect of periodontitis treatment on changes in blood inflammatory markers in patients with generalized aggressive periodontitis | Journal of Periodontal Research |
| E023 | Gaetano Isola | Effect of quadrantwise versus full‐mouth subgingival instrumentation on clinical and microbiological parameters in periodontitis patients: A randomized clinical trial | Journal of Periodontal Research |
| E024 | Gizem Torumtay Cin | The effects of severe periodontitis on arterial stiffness using cardio‐ankle vascular index in patients with type 2 diabetes | Journal of Periodontal Research |
| R001 | Lorenzo Tavelli | Coronally advanced flap versus tunnel technique for the treatment of peri‐implant soft tissue dehiscences with the connective tissue graft: A randomized, controlled clinical trial | Journal of Clinical Periodontology |
| R002 | Bin Cai | Platelet-rich plasma therapy for temporomandibular joint osteoarthritis: A randomized controlled trial | Journal of Cranio-Maxillo-Facial Surgery |
| R003 | Yuki Ichioka | Surface decontamination of explanted peri‐implantitis‐affected implants | Journal of Clinical Periodontology |
| R004 | Chun-Teh Lee | Clinical efficacy of soft‐tissue augmentation on tissue preservation at immediate implant sites: A randomized controlled trial | Journal of Clinical Periodontology |
| R008 | Hong-Chang Lai, Maurizio S. Tonetti | Improved positional accuracy of dental implant placement using a haptic and machine‐vision‐controlled collaborative surgery robot: A pilot randomized controlled trial | Journal of Clinical Periodontology |
| R009 | Hanna Enerbäck | Effect of high-fluoride toothpaste and mouth rinse on the prevention of demineralized lesions during orthodontic treatment: a randomized controlled trial | European Journal of Orthodontics |
| R011 | Baoxin Huang, Zhuofan Chen | Clinical and radiographic results of crestal vs. subcrestal placement of implants in posterior areas: A split‐mouth randomized controlled clinical trial | Clinical Implant Dentistry and Related Research |
| R012 | Daniel Hagenfeld | Long-term changes in the subgingival microbiota in patients with stage III–IV periodontitis treated by mechanical therapy and adjunctive systemic antibiotics: A secondary analysis of a randomized controlled trial | Journal of Clinical Periodontology |
| R013 | Peter Eickholz | Clinical benefits of systemic amoxicillin/metronidazole may depend on periodontitis stage and grade: An exploratory sub-analysis of the ABPARO trial | Journal of Clinical Periodontology |
| R014 | Jan Vacek | Phytocannabinoids and gingival inflammation: Preclinical findings and a placebo‐controlled double‐blind randomized clinical trial with cannabidiol | Journal of Periodontal Research |
| R015 | Mohammad Y Hajeer | Treatment effectiveness of young adults using clear aligners versus buccal fixed appliances in class I malocclusion with first premolar extraction using the ABO-Objective Grading System: A randomized controlled clinical trial | International Orthodontics |
| R019 | Ali Sadiq | A placebo‐controlled randomized clinical trial of antibiotics versus probiotics as an adjuvant to nonsurgical periodontal treatment among smokers with Stage III, Grade C generalized periodontitis | Clinical Advances in Periodontics |
| R020 | He Lu | Effects of amoxicillin and metronidazole as an adjunct to scaling and root planing on glycemic control in patients with periodontitis and type 2 diabetes: A short‐term randomized controlled trial | Journal of Periodontal Research |
| R021 | Miguel Padial-Molina | Periostin in the relation between periodontal disease and atherosclerotic coronary artery disease: A pilot randomized clinical study | Journal of Periodontal Research |
| R024 | Vincent J. J. Donker | Immediate implant placement with immediate or delayed provisionalization in the maxillary aesthetic zone: A 10‐year randomized trial | Journal of Clinical Periodontology |
| R026 | Franz Josef Strauss, Justin Pijpe | Alveolar ridge changes 1‐year after early implant placement, with or without alveolar ridge preservation at single‐implant sites in the aesthetic region: A secondary analysis of radiographic and profilometric outcomes from a randomized controlled trial | Clinical Implant Dentistry and Related Research |
| R027 | Stefan P. Bienz | Clinical and histological wound healing patterns of collagen‐based substitutes: An experimental randomized controlled trial in standardized palatal defects in humans | Journal of Clinical Periodontology |
| R028 | Vinay Kumar Chugh | Rate and anchorage loss during en‐masse retraction between friction and frictionless mechanics: A randomized clinical trial | Orthodontics & Craniofacial Research |
| R030 | Doaa Adel-Khattab | Injectable platelet rich fibrin versus hyaluronic acid with bovine derived xenograft for alveolar ridge preservation: A randomized controlled clinical trial with histomorphometric analysis | Clinical Implant Dentistry and Related Research |
| R031 | Yassir A. Yassir | Effectiveness of tubular coaxial nickel-titanium and copper nickel-titanium orthodontic aligning archwires: A randomized clinical trial | International Orthodontics |
| R032 | Mawia Karkoutly | Effectiveness of Ultrasound Therapy, TheraBite Device, Masticatory Muscle Exercises, and Stabilization Splint for the Treatment of Masticatory Myofascial Pain: A Randomized Controlled Trial | Clinical and Experimental Dental Research |
| R034 | Stina Hansson | Posterior crossbite corrections in the early mixed dentition with quad helix or rapid maxillary expander: a cost-effectiveness analysis of a randomized controlled trial | European Journal of Orthodontics |
| R036 | Giacomo Baima, Federica Romano | The flapless approach with and without enamel matrix derivatives for the treatment of intrabony defects: A randomized controlled clinical trial | Journal of Clinical Periodontology |
| R037 | Pieter Onclin | Two or four implants for maxillary overdentures in edentulous patients: 1-year results of a randomized controlled trial | Clinical Implant Dentistry and Related Research |
| R038 | Margherita Fontana | A Randomized Clinical Trial to Assess Caries Arrest by Using Silver Diamine Fluoride in U.S. Children: Interim Findings | Pediatric Dentistry |
| R039 | Anna Trullenque-Eriksson | Factors influencing outcomes of surgical therapy of peri‐implantitis: A secondary analysis of 1-year results from a randomized clinical study | Journal of Clinical Periodontology |
| R040 | João Gaspar | Osseodensification versus lateral window technique for sinus floor elevation with simultaneous implant placement: A randomized clinical trial on patient-reported outcome measures | Clinical Implant Dentistry and Related Research |
| R041 | Takeshi Ioroi | Efficacy and safety of ibuprofen gargle for postoperative pain after mandibular third molar extraction: A phase II, placebo‐controlled, double‐blind, randomized crossover trial | Clinical and Experimental Dental Research |
| R042 | Noha A. El-Ashmawi | Evaluation of Facial Esthetics Following NAM Versus CAD/NAM in Infants With Bilateral Cleft Lip and Palate: A Randomized Clinical Trial | The Cleft Palate-Craniofacial Journal |
| R043 | Iosif Sifakakis | The effect of mastic mouthwash on halitosis and oral hygiene in orthodontic patients: a randomized clinical trial | European Journal of Orthodontics |
| R044 | Hiba A. Fawakhiri | A 3‐year controlled clinical trial comparing high‐translucency zirconia (cubic zirconia) with lithium disilicate glass ceramic (e.max) | Clinical and Experimental Dental Research |
| R045 | Mawia Karkoutly | The efficacy of the dental Water Jet, orthodontic, and conventional toothbrushes in plaque removal around orthodontic braces in adolescents: A randomized controlled trial | Clinical and Experimental Dental Research |
| R046 | Thomas Starch-Jensen | Maxillary sinus membrane elevation and coagulum compared with maxillary sinus floor augmentation and a composite graft: A 1‐year single‐blinded randomized controlled trial | Clinical Implant Dentistry and Related Research |
| R047 | Julia Naoumova | Failure frequency of fixed mandibular retainers after pre-treatment of the enamel surface with pumice versus sandblasting—a randomized controlled trial | European Journal of Orthodontics |
| R048 | Yoshio Yamashita | Effectiveness of perioperative oral hygiene management using a cetylpyridinium chloride‐, dipotassium glycyrrhizinate, and tranexamic acid‐based mouthwash as an adjunct to mechanical oral hygiene in patients with maxillomandibular fixation: A randomized controlled clinical trial | Clinical and Experimental Dental Research |
| R050 | Evelina Kratunova | Articaine Infiltration Versus Lidocaine Inferior Alveolar Nerve Block for Primary Mandibular Molars: A Double-blind Randomized Controlled Clinical Trial | Pediatric Dentistry |
| R051 | Alberto Monje | Defect angle as prognostic indicator in the reconstructive therapy of peri‐implantitis | Clinical Implant Dentistry and Related Research |
| R053 | Perihan Dalgalı | Evaluating the Impact of Different Education Methods on Cleft Lip and Palate Anatomy Training | The Cleft Palate Craniofacial Journal |
| R054 | Mawia Karkoutly | Comparative efficacy of topical povidone‐iodine and chlorhexidine gel on dental plaque regrowth in toddlers: A randomized controlled trial | Clinical and Experimental Dental Research |
| R055 | Ai Fujimoto | Changes in oral microflora following 0.3% cetylpyridinium chloride‐containing mouth spray intervention in adult volunteers after professional oral care: Randomized clinical study | Clinical and Experimental Dental Research |
| R057 | Betül Şen Yavuz | A Randomized Clinical Trial of Short Fiber Reinforced Composite and Glass Hybrid Restoration for Molars Affected by Molar Hypomineralization | Pediatric Dentistry |
| R058 | S. C. Gomes | Supportive periodontal care with or without subgingival instrumentation: Microbiological results of a 2‐year randomized clinical trial | Journal of Clinical Periodontology |
| R059 | Mohammad Y. Hajeer | Adult periodontal comparison after treatment of palatally impacted canines aligned by conventional or accelerated minimally-invasive corticotomy-assisted orthodontic traction: A randomized controlled trial | International Orthodontics |
| R060 | Thikriat Al-Jewair | Comparison of GUMMETAL and stainless steel alloy during canine retraction: A pilot split-mouth randomized controlled trial | International Orthodontics |
| R061 | Linhong Wang | Alveolar ridge preservation in sockets with severe periodontal destruction using autogenous partially demineralized dentin matrix: A randomized controlled clinical trial | Clinical Implant Dentistry and Related Research |
| R062 | Sara Waldenström | Comparison of chairside rectangular chain retainers and multi-stranded conventional retainers: a randomized controlled trial | European Journal of Orthodontics |
| R063 | Kathrin Becker | Eligibility and efficacy of a CPC‐ and CHX‐based antiviral mouthwash for the elimination of SARS‐CoV‐2 from the saliva: A randomized, double‐blind, controlled clinical trial | Journal of Clinical Periodontology |
| R065 | Yasser Alsayed Tolibah | Evaluation of the modified 3Mix‐Simvastatin combination in non‐instrumental endodontic therapy of necrotic primary molars: A two‐arm randomized controlled trial | Clinical and Experimental Dental Research |
| R066 | Tuqa Rashad Raghis | Skeletal and dentoalveolar changes after total maxillary arch distalization using the casted palatal plate vs. buccal miniscrews: A randomized clinical trial | International Orthodontics |
| R067 | Cho-Ying Lin | Half‐ and full‐grafting alveolar ridge preservation with different sealing materials: A three‐arm randomized clinical trial | Clinical Implant Dentistry and Related Research |
| R068 | Giacomo Baima | Influence of locally delivered doxycycline on the clinical and molecular inflammatory status of intrabony defects prior to periodontal regeneration: A double‐blind randomized controlled trial | Journal of Periodontal Research |
| R070 | Yasser Alsayed Tolibah | Liquid platelet‐rich fibrin in root surface biomodification during gingival recession treatment: Randomized, controlled, split‐mouth, clinical trial | Clinical and Experimental Dental Research |
| R071 | Asli Baysal | The effect of micro-osteoperforation on the rate of tooth movement during the alignment stage in patients with mandibular crowding: a randomised controlled trial | European Journal of Orthodontics |
| R072 | Atiphan Pimkhaokham | Effect of chemical and electrochemical decontamination protocols on single and multiple‐used healing abutments: A comparative analysis of contact surface area, micro‐gap, micro‐leakage, and surface topography | Clinical Implant Dentistry and Related Research |
| R073 | Ammar Salim Kadhum | Poly-Ether-Ether-Ketone versus dead-soft coaxial bonded retainers: a randomized clinical trial. Part 2: periodontal health and microbial biofilm assessment | European Journal of Orthodontics |
| R074 | Juan Carlos Bernabeu-Mira | Autologous bone harvested during implant bed preparation: A randomized clinical trial comparing high‐speed drilling with irrigation versus low‐speed drilling without irrigation | Clinical Implant Dentistry and Related Research |
| R075 | Lorenzo Franchi | Comparison between digital and conventional impression techniques in children on preference, time and comfort: A crossover randomized controlled trial | Orthodontics & Craniofacial Research |
| R077 | Doaa Adel-Khattab | Volumetric assessment of volume stable collagen matrix in maxillary single implant site development: A randomized controlled clinical trial | Clinical Implant Dentistry and Related Research |
| R078 | Gilan Y. Altonbary | Accuracy of digital and conventional implant-level impression techniques for maxillary full-arch screw-retained prosthesis: A crossover randomized trial | Clinical Implant Dentistry and Related Research |
| R079 | B. Bharghavi Patnaik | Peri-implant mucosal enhancement using leukocyte platelet rich fibrin under Sohn’s poncho technique: A randomized controlled clinical trial | Clinical Advances in Periodontics |
| R080 | Leila Nazari | The effect of different concentrations and temperatures of sodium hypochlorite irrigation on pain intensity following endodontic treatment of mandibular molars with irreversible pulpitis: A randomized, double‐blind clinical trial | Clinical and Experimental Dental Research |
| R081 | Luigi Barbato | Non‐surgical retreatment versus papillary preservation flap surgery for residual pockets: A randomized controlled trial with clinical and patient‐reported outcomes | Journal of Clinical Periodontology |
| R082 | Yassir A. Yassir | Evaluation of failure rate of molar tubes with a modified bonding technique: a randomized clinical trial | European Journal of Orthodontics |
| R083 | Xing Wang, Xiuyun Ren, Bin Zhao | Effect of impacted mandibular third molar extraction on periodontal microbiota and clinical parameters of adjacent teeth: A randomized clinical trial | Journal of Cranio-Maxillo-Facial Surgery |
| R084 | *Atiphan Pimkhaokham* | Gene expression, micro‐CT and histomorphometrical analysis of sinus floor augmentation with biphasic calcium phosphate and deproteinized bovine bone mineral: A randomized controlled clinical trial | Clinical Implant Dentistry and Related Research |
| R086 | *E. S. Radwan* | Effect of functional appliances on sleep-disordered breathing in Class II division 1 malocclusion children: Randomized controlled trial | Orthodontics & Craniofacial Research |
| R087 | Ama Johal | Long-term comparison of the efficacy of manual versus powered tooth brushing in adolescent orthodontic patients: a single-centre, parallel design randomized clinical trial | European Journal of Orthodontics |
| R089 | Luis Huanca Ghislanzoni | Headgear therapy in children with Class II malocclusion and the role of compliance on treatment outcome: A nine‐month randomized controlled trial | Orthodontics & Craniofacial Research |
| R091 | Inas Adel Mahmoud | Assessment of crestal bone loss and periodontal parameters of polymer infiltrated ceramic network versus lithium disilicate implant hybrid abutment crowns in the esthetic zone (A randomized clinical trial) | Clinical Implant Dentistry and Related Research |
| R092 | Xuedong Yuan, Hu Long | Artificial intelligence‐assisted determination of available sites for palatal orthodontic mini implants based on palatal thickness through CBCT | Orthodontics & Craniofacial Research |
| R093 | Sarah Al-Rihaymee | Platelet-rich fibrin as an adjunct to scaling and root planing in treatment of shallow periodontal pockets: A randomized clinical trial | Journal of Oral Biosciences |
| R094 | Mohammad Younis Hajeer | Evaluation of the effectiveness of the platelet-rich plasma compared to the injectable platelet-rich fibrin on the rate of maxillary canine retraction: a three-arm randomized controlled trial | European Journal of Orthodontics |
| R095 | Emad Eddin Alzoubi | The effect of tooth borne versus skeletally anchored Alt-RAMEC protocol in early treatment of Class III malocclusion: a single-centre randomized clinical trial | European Journal of Orthodontics |
| R096 | Takeshi Kikuchi, Akio Mitani | Effectiveness of novel oral hygiene instruction avoiding inattentional blindness using an application for unique plaque control record calculation: A randomized clinical trial | Journal of Periodontal Research |
| R100 | Abdelsalam Elaskary | The bone shielding versus dual‐zone concept in treating thin‐walled fresh extraction sockets with immediate implant placement: Soft and hard tissue changes. A randomized clinical trial | Clinical Implant Dentistry and Related Research |
| R101 | Max Hogrebe | Covering skin defects with a xenogeneic collagen matrix in comparison with a skin graft – A multicenter randomized controlled trial | Journal of Cranio-Maxillo-Facial Surgery |
| R102 | Subash Chandra Raj | Comparative evaluation of the clinical and radiographic efficacy of 0.05% zoledronate gel as local drug delivery system in treating intrabony defects in stage III grade B periodontitis patients with and without type‐2 diabetes mellitus—A randomized split‐mouth clinical trial | Clinical Advances in Periodontics |
| R103 | Prasanth D Shunmuga | Clinical evaluation of the combined efficacy of injectable platelet‐rich fibrin along with scaling and root planing in the non‐surgical periodontal therapy of stage III and grade C periodontitis patients having type 2 diabetes mellitus: A randomized controlled trial | Clinical Advances in Periodontics |
| R104 | Shrutha Havale | Use of Three Pre-Injection Procedures to Reduce Pain Perception of Intraoral Injections in Eight- to 12-Year-Old Children: Randomized Controlled Trial | Pediatric Dentistry |
| R106 | Luigi Canullo | Effect of argon plasma abutment activation on soft tissue healing: RCT with histological assessment | Clinical Implant Dentistry and Related Research |
| R107 | Wenjie Hu | Assessment of soft and hard tissue changes following micro crestal flap—Alveolar ridge preservation and augmentation at molar extraction sites in patients with stage III/IV periodontitis: A randomized controlled trial | Journal of Clinical Periodontology |
| R109 | Naif Ghanem | Clinical performance of custom‐milled polyetherketoneketone (PEKK) posts and cores: A 12‐month follow‐up randomized controlled pilot study | Clinical and Experimental Dental Research |
| R110 | Fabio Camacho-Alonso | Randomized split‐mouth clinical trial comparing osteoblastic activity and osteogenic potential of autogenous particle harvesting during implant surgery without irrigation and with irrigation | Clinical Implant Dentistry and Related Research |
| R112 | Ammar Ibrahim | Alveolar Ridge Preservation With Fibro‐Gide or Connective Tissue Graft: A Randomized Controlled Trial of Soft and Hard Tissue Changes | Clinical and Experimental Dental Research |
| R113 | Ammar Salim Kadhum | Poly-Ether-Ether-Ketone versus dead-soft coaxial bonded retainers: a randomized clinical trial. Part 2: periodontal health and microbial biofilm assessment | European Journal of Orthodontics |
| R114 | *Fernando Suárez-L opez del Amo* | Volumetric changes and graft stability after lateral window sinus floor augmentation: A randomized clinical trial | Clinical Implant Dentistry and Related Research |
| R116 | Caroline M. Sawicki | Preoperative Multisensory Room Use in Pediatric Patients With Autism: A Randomized Clinical Trial | Pediatric Dentistry |
| R117 | Mohammad Mohammadi | Horizontal ridge augmentation with particulate cortico‐cancellous freeze‐dried bone allograft alone or combined with injectable‐platelet rich fibrin in a randomized clinical trial | Clinical Implant Dentistry and Related Research |
| R118 | Roberto Farina | Efficacy of communicating periodontal risk on psychological outcomes and supragingival plaque control in patients undergoing first periodontal consultation: A parallel‐arm, randomized trial | Journal of Clinical Periodontology |
| R120 | Tippanart Vichayanrat | Effect of dentures and dietary advice on protein intake in older Thai adults with missing posterior occluding teeth | Gerodontology |
| R121 | Valeria Viarani | Cephalometric changes of pushing splints 3 compared to rapid maxillary expansion and facemask therapy on the airway space in class III growing patients: A randomized clinical trial | Orthodontics & Craniofacial Research |
| R122 | Rodrigo Lorenzi Poluha | The benefits of performing temporomandibular joint arthrocentesis with catheters and a vacuum pump: A randomized control trial | Journal of Cranio-Maxillo-Facial Surgery |
| R123 | Essam Ahmed Al-Moraissi | Radiographic evaluation of alveolar ridge preservation using a chitosan/polyvinyl alcohol nanofibrous matrix: A randomized clinical study | Journal of Cranio-Maxillo-Facial Surgery |
| R124 | *Liselotte Paulsson* | Self‐reported pain during different phases of orthodontic treatment with fixed appliance: A multi‐centre randomized controlled trial in adolescents with crowding | Orthodontics & Craniofacial Research |
| R126 | Mikael Sonesson | Stability of maxillary anterior teeth during retention and 1 year after removal of retention—an RCT on adolescents retained with two different bonded retainers and a vacuum-formed retainer | European Journal of Orthodontics |
| R127 | Annie Hendry | Using a theoretically informed process evaluation alongside a trial to improve oral health for care home residents | Gerodontology |
| R128 | Reza A. Mokhtari | A randomized, multicenter, double‐blinded parallel study to evaluate the safety and performance of zoledronate‐coated versus uncoated dental implants in partially edentulous patients | Clinical Implant Dentistry and Related Research |
| R129 | Stefan Renvert | The efficacy of reconstructive therapy in the surgical management of peri‐implantitis: A 3‐year follow‐up of a randomized clinical trial | Journal of Clinical Periodontology |
| R130 | Dan Ericson | Effect of a single application of silver diamine fluoride on root caries after 12 months in institutionalised older adults—A randomised clinical trial | Gerodontology |
| R131 | Ignacio Sanz-Sánchez | Effect of one‐time placement of the definitive abutment versus multiple healing abutment disconnections and reconnections during the prosthetic phase on radiographic and clinical outcomes: A 12‐month randomized clinical trial | Clinical Implant Dentistry and Related Research |
| R135 | Discepoli Nicola | Treatment of peri‐implant mucositis: Adjunctive effect of glycine powder air polishing to professional mechanical biofilm removal. 12 months randomized clinical study | Clinical Implant Dentistry and Related Research |
| R136 | Marwa Ahmed Aboelez | Vertical versus angled immediately loaded implants for assisting maxillary overdentures with locator attachments: A preliminary results of one-year randomized clinical trial | Clinical Implant Dentistry and Related Research |
| R137 | *T. R. Prasanna Arvind* | Three-dimensional volumetric evaluation of root resorption in maxillary anteriors following en-masse retraction with varying force vectors –a randomized control trial | Orthodontics & Craniofacial Research |
| R138 | Tiago Fialho | Comparison of the efficiency of initial dental alignment with Invisalign® aligners changed every 7 or 14 days in mature adults: Randomized clinical trial | Orthodontics & Craniofacial Research |
| R139 | Rodrigo Lorenzi Poluha | Comparative study of arthrocentesis with concentric-needle cannula with classic concentric needle: A randomized single-blind controlled clinical trial | Journal of cranio-Maxillo-Facial Surgery |
| R140 | Sally Magdi | Friction versus frictionless mechanics during maxillary en-masse retraction in adult patients with Class I bimaxillary dentoalveolar protrusion: a randomized clinical trial | European Journal of Orthodontics |
| R141 | Seung-Yun Shin | Soft tissue outcomes following alveolar ridge preservation with/without primary flap closure for periodontally damaged extraction socket: A randomized clinical trial | Clinical Implant Dentistry and Related Research |
| R142 | B. Sasikala | Evaluation of the efficacy of autologous conditioned serum versus dextrose prolotherapy in internal derangement of the TMJ — A pilot study | Journal of Cranio-Maxillo-Facial Surgery |
| R143 | Fatemeh Tavakoli | Evaluation of the Effects of Punica granatum Flower Tablets on Pain and Healing of Minor Recurrent Aphthous Stomatitis: A Randomized Clinical Trial | Clinical and Experimental Dental Research |
| R144 | Silvio Augusto Bellini-Pereira | Treatment stability with bonded versus vacuum‐formed retainers after 12 months in adolescents and young adults: A randomized clinical trial | Orthodontics & Craniofacial Research |
| R145 | Pavithran Ashokkumar | Effects of melatonin on postoperative pain and sensory recovery following zygomaticomaxillary complex fractures — A randomized controlled trial | Journal of Cranio-Maxillo-Facial Surgery |
| R146 | Vincent J. J. Donker | Digital versus conventional surgical guide fabrication: A randomized crossover study on operator preference, difficulty, effectiveness, and operating time | Clinical and Experimental Dental Research |
| R147 | Karim M. Fawzy El-Sayed | Micro‐needling versus acellular dermal matrix in RT1 gingival recession coverage: A randomized clinical trial | Journal of Periodontal Research |
| R148 | Javed Ashraf | Effectiveness of auditory distraction on the management of dental anxiety in patients undergoing tooth extraction at a tertiary care hospital in Islamabad | Clinical and Experimental Dental Research |
| R149 | Mohamed Shawky | Assessment of bone gain and neurosensory affection with the sandwich osteotomy technique for vertically deficient posterior mandible using a full digital workflow versus conventional protocol: A randomized split mouth study | Clinical Implant Dentistry and Related Research |
| R150 | Ning Li | Parotideomasseteric fascia flap is an effective management for prevention of postoperative salivary collection arising from radical surgery of oral cancer | Journal of Cranio-Maxillo-Facial Surgery |
| R151 | Gianrico Spagnuolo | Effect of Needle Insertion Angle on Pain During Labial Infiltration Anesthesia of the Anterior Maxilla: A Randomized Clinical Trial | Clinical and Experimental Dental Research |
| HS001 | Yoshiko Ariji | Effects of the combined use of segmentation or detection models on the deep learning classification performance for cyst‐like lesions of the jaws on panoramic radiographs: Preliminary research | Oral Science International |
| HS002 | Ana Carolina Fragoso Motta | Impairment of oral health and its impact on the quality of life of patients with Sjögren’s syndrome | Oral Science International |
| HS003 | Oguzhan Baydar | Comparison of linear and angular measurements in mandibular symphyseal region using lateral cephalometric and cone beam CT images | Oral Science International |
| HS004 | Eri Sawada | Comparing apparent diffusion coefficient values of the masticatory muscles on diffusion‐weighted magnetic resonance imaging in patients with temporomandibular joint osteoarthrosis | Oral Science International |
| HS005 | Akifumi Enomoto | Prevalence of oral chronic graft versus host disease after allogeneic stem cell transplantation | Oral Science International |
| HS006 | Mikihiko Kogo | 3D morphological changes of the lip and face in patients with cleft lip and alveolus: The comparison of the lip development of incomplete and complete cleft lips under 3 months of age | Oral Science International |
| HS007 | Murat Aydin | Quantifying the ice test in halitosis patients | Oral Science International |
| HS008 | Hirotaka Muraoka | Efficacy of apparent diffusion coefficient values for normal submandibular glands and acute submandibular sialadenitis | Oral Science International |
| HS009 | Naohisa Hirahara | The association between rheumatoid arthritis and masticatory muscles through the utilization of diffusion‐weighted magnetic resonance imaging | Oral Science International |
| HS010 | Afagh Moattari | Correlation between sampling site and prevalence of human papilloma virus (HPV) in patients with oral cancer referred to Shiraz, Iran | Oral Science International |
| HS011 | Serkan Taş | Cross-cultural adaptation, validity, and reliability of the Turkish version of the Oral Behaviours Checklist | Oral Science International |
| HS012 | Yoshitaka Kise | Performance of a deep learning system for simultaneously diagnosing radiolucent and radiopaque lesions in the anterior maxilla on panoramic radiographs | Oral Science International |
| HS013 | Kazumichi Sato | Prevalence of panoramic radiographs findings of cementum-osseous dysplasia with calcification in the department of oral surgery at hospital: A retrospective study | Oral Science International |
| HS014 | Taner Ozturk | Assessment of crista galli morphology and morphometry in individuals with cleft lip and palate: A three-dimensional analysis | Oral Science International |
| HS015 | Ken-ichiro Sakata | Serum albumin is a potential predictor of delayed healing after minor dental surgery among older patients aged over 65 years: A retrospective study | Oral Science International |
| HS016 | Dr. Keiko Aota | Activation of Janus kinase 2 contributes to the autoimmune pathology in the salivary glands of patients with Sjögren's syndrome | Oral Science International |
| HS017 | Kwan-Soo Park | Risk of bleeding during flapless immediate implant placement after tooth extraction in patients taking antithrombotics | Oral Science International |
| HS018 | Michitaka Somoto | Effect of mental arithmetic stress on periodontal sensation in the upper molar and tactile sensation in the cheek | Oral Science International |
| HS019 | Shigehiro Abe | Clinical study on the impact of the COVID-19 pandemic on mandibular fractures at a regional core hospital for emergency and critical care medicine in Tokyo, Japan | Oral Science International |
| HS020 | Gottumukkala NVS Sruthima | Clinical and patient reported outcomes using autologous fibrin sealant as adjunct to diode light amplification by stimulated emission of radiation-assisted gingival depigmentation: A randomized controlled trial | Oral Science International |
| HS021 | Ryutaro Ono | Elevated alveolar bone mineral density in female patients undergoing bisphosphonate therapy: A pilot cross-sectional study using intraoral radiographs | Oral Science International |
| HS022 | Kanji Nohara | Olfactory and gustatory functions, appetite, and nutritional status in Alzheimer's disease in a care facility | Oral Science International |
| HS023 | Enrica Giammarinaro | The Effectiveness of an Incremental Approach to Nonsurgical Periodontal Therapy with the Use of Adjunctive Slow-Release Locally Administered 0.02% Hypochlorite Formulation: A Randomized Clinical Study | Journal of International Oral Health |
| HS024 | Irna Sufiawati | Comparison of Total Antioxidant Capacity Level in Patients with HIV/AIDS and Healthy Individuals | Journal of International Oral Health |
| HS025 | Sagnik Bhattacharya | Correlation between Dental Caries and Salivary pH, Mineral Content, and Salivary Immunoglobulin Levels in Adult Population of Northern Suburban Region of Kolkata, India | Journal of International Oral Health |
| HS026 | Aravind Kumar Subramanian | Posteroanterior Cephalometric Immediate Assessment of Infrazygomatic Crest Mini-Implants: A Retrospective Study | Journal of International Oral Health |
| HS027 | Elizangela Partata Zuza | Palatogingival Groove: Prevalence, Characteristics and Implications in a Cross-Sectional Study in Rio de Janeiro-Brazil | Journal of International Oral Health |
| HS028 | Hassan M. H. Negm | Do Polymorphisms Predispose Dental Caries Susceptibility in Egyptian Adults? A Cross-Sectional Study | Journal of International Oral Health |
| HS029 | Rekha R. Koduganti | Evaluation of Root Biomodification as an Adjunct to Platelet-Rich Fibrin Versus Amniotic Membrane and Coronally Advanced Flap in Class I and Class II Gingival Recession Defects: A Randomized Controlled Study | Journal of International Oral Health |
| HS030 | Anushree Ningombam | Comparative Estimation of C-terminal Cross-linked Telopeptide of Type I Collagen Levels in Gingival Crevicular Fluid in Diabetic and Nondiabetic Patients with Chronic Periodontitis: A Prospective Clinical Study | Journal of International Oral Health |
| HS031 | Ungsinun Intarakamhang | Development and Validation of “OHL-Ortho” Measurement Tool and Causal Model of Oral Health Behavior among Adult Orthodontic Patients | Journal of International Oral Health |
| HS032 | Arvina Rajasekar | Quantification of Red Complex Microorganisms among Patients with Different Surface-Modified Dental Implants: A Prospective Clinical Study | Journal of International Oral Health |
| HS033 | Karthikeya Patil | Facilitated Self-Medication Practice in Pharmacies in Combating Oral Health Needs: A Questionnaire-Based Study | Journal of International Oral Health |
| HS034 | Ungsinun Intarakamhang | Development of Oral Health Program on Knowledge and Oral Health Literacy among Students and Families | Journal of International Oral Health |
| HS035 | Mai Zakaria | Association Between Periodontitis and Hypertension Among a Sample of Adult Egyptian Patients: A Hospital-Based Cross-Sectional Study | Journal of International Oral Health |
| HS036 | Donald B. Giddon | No Correlation between Sexually Dimorphic Ratios of the Sizes of Fourth to Second Digits and Hypothesized Ratios of the Sizes of the Central to Lateral Incisors | Journal of International Oral Health |
| HS037 | Mohamed Hassan | A Cross-Sectional Analysis of Prevalence of Dental Anomalies by CBCT Images: An Institutional-Based Retrospective Study | Journal of International Oral Health |
| HS038 | Nasir H. Siti | Orthodontic Patient’s Perspectives, Attitudes, and Readiness Toward Teleorthodontics | Journal of International Oral Health |
| HS039 | César Cayo-Rojas | Anxiety Associated Painful Temporomandibular Disorders in Peruvian Dental Students During the Major Waves of COVID-19 Infection: A Cohort Study | Journal of International Oral Health |
| HS040 | Nawwal Alwani Mohd Radzi | Perceived Barriers in Digitalizing Oral Health Promotion: Phenomenological Study among Malaysian Dental Public Health Specialists | Journal of International Oral Health |
| HS041 | Lavanya Govindaraju | Quality of Obturation and the Behavior of Children Treated Under Nitrous Oxide Conscious Sedation: A Double-Blinded Split-Mouth Randomized Clinical Trial | Journal of International Oral Health |
| HS042 | Arvina Rajasekar | Efficacy of Fibro-Gide® in Coronally Advanced Flap for the Treatment of Multiple Gingival Recession: A Prospective Clinical Study | Journal of International Oral Health |
| HS043 | Rania S. Mosallam | Clinical Evaluation of “Snowplow” Technique Versus Bulk-Fill Technique in Restoration of Class II Cavities: A Randomized Clinical Trial | Journal of International Oral Health |
| HS044 | Eriska Riyanti | Evaluation of Dentists’ Knowledge in Bandung City Regarding Patients with Special Health Care Needs and Behavior Management in Dental Care | Journal of International Oral Health |
| HS045 | Julissa Amparo Dulanto Vargas | Accuracy of the Mincer Method for Dental Age Estimation in a Honduran Population | Journal of International Oral Health |
| HS046 | Boosana Kaboosaya | Impact of Interproximal Features on Marginal Bone Level Changes of Autograft, Allograft, and Xenograft after Functional Loading: A Retrospective Study | Journal of International Oral Health |
| HS047 | Asem M. Kamel | Expression of Regulatory T Cell and Related Interleukins in Gingivitis Versus Stage 3, Grade B Generalized Periodontitis: Synergy or Cacophony—A Cross-Sectional Study | Journal of International Oral Health |
| HS048 | Arif Yezdani | Serum Osteocalcin – A Biochemical Marker for Pubertal Growth Assessment | APOS Trends in Orthodontics |
| HS049 | Neam Agha | Assessment of Arch Dimensions in Children with Unilateral Cleft Lip and Palate Treated Surgically by Furlow Double-Opposing Z-Plasty Protocol | APOS Trends in Orthodontics |
| HS050 | Mathew Thomas Maliael | Quantitative Assessment of Ramal Bone Width and the Proximity of the Inferior Canal for the Predictable Insertion of Ramal Implants: Cone-Beam Computed Tomography Study | APOS Trends in Orthodontics |
| HS051 | Fabio Ramina | Marginal ridge alignment and interproximal bone levels: Evaluation of a possible correlation | APOS Trends in Orthodontics |
| HS052 | Nabeel Almotairy | Age and gender differences in maxillary canine impaction severity and orthodontic treatment difficulty – A retrospective cross-sectional study | APOS Trends in Orthodontics |
| HS053 | Sridevi Padmanabhan | Temporomandibular joint and skeletal changes in response to Twin block and Advansync appliance therapy – A three dimensional study | APOS Trends in Orthodontics |
| HS054 | Yiqiang Qiao and Abdelrahman M.A. Mohamed | Cephalometric evaluation of posterior airway space in Chinese and Egyptian races | APOS Trends in Orthodontics |
| HS055 | Zeynep Çoban Büyükbayraktar | The effects of orthodontic treatment type, depression, and treatment need on perceived pain intensity | APOS Trends in Orthodontics |
| HS056 | Adnan Sarfaraz Khan | Evaluation of a correlation between the severity of root resorption of adjacent teeth and level of difficulty of impacted canine – A cone-beam computed tomography study | APOS Trends in Orthodontics |
| HS057 | Mona Bajestan | An evaluation of maxillary expansion after Phase I orthodontic treatment with clear aligners using model analysis and cone-beam computed tomography | APOS Trends in Orthodontics |
| HS058 | Yu Li | A standardized hands-on protocol effectively enhanced orthodontic education on bonding clear aligner attachments | APOS Trends in Orthodontics |
| HS059 | Paolo Manzo | Accuracy of the infrazygomatic orthodontic bone screws digital planning and surgical guided positioning: An observational study | APOS Trends in Orthodontics |
| HS060 | Benny M. Soegiharto | Indonesian orthodontists and laypeople’s perception of the four components of smile analysis in individuals with various vertical skeletal patterns | APOS Trends in Orthodontics |
| HS061 | Farah Yousry Eid | Cone-beam computed tomography assessment of bone quality and quantity following laser-assisted orthodontic tooth movement: A randomized controlled trial | APOS Trends in Orthodontics |
| HS062 | Matthew B. Harper | Mandibular canine development as an indicator of maxillary cleft site canine development | APOS Trends in Orthodontics |
| HS063 | Satinder Pal Singh | Effect of maxillary distraction osteogenesis and LeFort-1 advancement orthognathic surgery on soft-tissue thickness and anterior soft-tissue to hard-tissue movement ratios among patients with complete unilateral cleft lip and palate | APOS Trends in Orthodontics |
| HS064 | Ankita Shriram Khurdal | Evaluation of augmented reality and social media on patient motivation to undergo fixed orthodontic treatment | APOS Trends in Orthodontics |
| HS065 | Satinder Pal Singh | Comparison of alignment efficacy and arch dimensions changes with superelastic-, heat-activated-, and seven stranded coaxial nickel-titanium archwires during fixed orthodontic treatment – A double-blind randomized clinical trial | APOS Trends in Orthodontics |
| HS066 | Eenal Bhambri | Effect of leukocyte platelet-rich fibrin on the rate of canine movement – A prospective and randomized control trial | APOS Trends in Orthodontics |
| HS067 | Chen-Jung Chang | Investigation of the correlation of midpalatal suture maturation stage with chronological age and cervical vertebral maturation stage | APOS Trends in Orthodontics |
| HS068 | Nazanin Nasr | Evaluation of natural head position changes in the treatment of class II malocclusions with functional appliance | APOS Trends in Orthodontics |
| HS069 | Li Qian | Orthodontic traction of impacted teeth involving gold chain bonding: A retrospective study on success rate and associated factors | APOS Trends in Orthodontics |
| HS070 | Nawaf Hamad Al Shammary | Adherence in adult orthodontic settings: Understanding orthodontists’ predictors | APOS Trends in Orthodontics |
| HS071 | Elena Galan | THE PREVALENCE OF MANDIBULAR MIDLINE DEVIATION IN CHILDREN AND ADOLESCENTS WITH DIFFERENT TYPES OF MALOCCLUSIONS | Romanian Journal of Oral Rehabilitation |
| HS072 | Stefana Luca | OPTIMIZING WOUND CARE: THE IMPORTANCE OF NEGATIVE PRESSURE WOUND THERAPY FOR VARIOUS ETIOLOGIES AND LOCALIZATIONS | Romanian Journal of Oral Rehabilitation |
| HS073 | Norina Forna, Lecturer Claudiu Topoliceanu claudiu | ASSESSMENT OF FACTORS INFLUENCING THE ESTHETIC, FUNCTIONAL AND BIOLOGICAL STATUS OF POSTERIOR COMPOSITE RESINS RESTORATIONS | Romanian Journal of Oral Rehabilitation |
| HS074 | Aureliana Caraiane, Doriana Agop Forna | IMPROVING BONE DENSITY BY USING VERSAH BURS IN ORAL IMPLANTOLOGY | Romanian Journal of Oral Rehabilitation |
| HS075 | Melania Olimpia Cojocaru | EVALUATION OF METABOLIC BIOCHEMICAL TESTS, BODY MASS INDEX VALUES AND PERIODONTAL / PERI-IMPLANT CLINICAL PARAMETERS OF PATIENTS REHABILITATED WITH PROSTHETIC DENTAL IMPLANTS OF AT LEAST 1 YEAR | Romanian Journal of Oral Rehabilitation |
| HS076 | Valentina Trifan | COMPLEX APPROACH IN THE TREATMENT OF ANGLE CLASS III MALOCLUSION | Romanian Journal of Oral Rehabilitation |
| HS077 | Cristina Maria Mihai, Alexandru Cosmin Pantazi, Iuliana Magdalena Starcea | EVALUATION OF RISK FACTORS FOR RECURRENT DIABETIC KETOACIDOSIS IN CHILDREN WITH TYPE 1 DIABETES | Romanian Journal of Oral Rehabilitation |
| HS078 | Florina Popa, Constantin Marcu | DENTAL DISEASE IN CHILDREN DIAGNOSED WITH LIFE-LIMITING DISEASES. RETROSPECTIVE STUDY | Romanian Journal of Oral Rehabilitation |
| HS079 | Oana Mihalache | CERVICAL COLUMN INJURIES AND THEIR MANAGEMENT DURING THE SARS COV 2 PANDEMIC | Romanian Journal of Oral Rehabilitation |
| HS080 | Magda Cuciureanu | HEARING LOSS PATIENTS` TREATMENT APPROACHES, MENTAL HEALTH ISSUES, AND SOCIAL ORAL PATHOLOGIES- PART 1 | Romanian Journal of Oral Rehabilitation |
| HS081 | Elena Roxana Bogdan Goroftei, Cristian Guțu | OBSERVATIONAL STUDY OF POST-COVID-19 SYNDROME IN HEALTH CARE WORKERS INFECTED WITH SARS-COV-2 VIRUS: GENERAL AND ORAL CAVITY COMPLICATIONS | Romanian Journal of Oral Rehabilitation |
| HS082 | Dan Lolos | THE SIGNIFICANCE OF COLOR CHOICE IN AESTHETIC ALL-CERAMIC RESTORATIONS: A COMPARATIVE ANALYSIS BETWEEN CLINICIANS AND PATIENTS | Romanian Journal of Oral Rehabilitation |
| HS083 | Manuela Lalu | HEADACHE, TEMPOROMANDIBULAR DISORDERS AND SELF-MEDICATION: FINDINGS FROM A LARGE SAMPLE QUESTIONNAIRE STUDY INVOLVING 1019 PARTICIPANTS | Romanian Journal of Oral Rehabilitation |
| HS084 | Cătalina Iulia Săveanu, Toma Vasilica, Dror Aizenbud | CONGENITALLY MISSING OF THE MAXILLARY LATERAL INCISOR: ORTHODONTIC CLOSURE OF THE SPACE | Romanian Journal of Oral Rehabilitation |
| HS085 | Alexandra Roi | THE EVALUATION OF LOCO-REGIONAL ANESTHESIA ACCIDENTS AND COMPLICATIONS IN ORAL AND MAXILLOFACIAL SURGERY | Romanian Journal of Oral Rehabilitation |
| HS086 | Tatiana Chisnoiu, Adriana Luminița Bălașa | INTERPLAY BETWEEN INTESTINAL MICROBIOTA AND RISK FACTORS IN THE FIRST THREE YEARS OF LIFE | Romanian Journal of Oral Rehabilitation |
| HS087 | Laurenta Lelia Mihai, Ana Maria Sciuca | THE IMPACT OF DENTAL ANXIETY ON PERIODONTAL DISEASE | Romanian Journal of Oral Rehabilitation |
| HS088 | Mihaela Jana Tuculina, Bogdan Dimitriu | PREVALENCE AND ANATOMICAL PARTICULARITIES OF THE SECOND MESIOBUCCAL CANAL OF THE PERMANENT UPPER FIRST MOLAR IN THE ROMANIAN POPULATION IN A CLINICAL ENVIRONMENT USING CONE BEAM COMPUTED TOMOGRAPHIC IMAGING- IN VIVO STUDY | Romanian Journal of Oral Rehabilitation |
| HS089 | Oana-Elena Ciurcanu, Carmen Nicoleta Savin | DENTAL HEALTHCARES SYSTEM DURING THE COVID-19 PANDEMIC AND IMPACT OF DIGITAL TECHNOLOGIES | Romanian Journal of Oral Rehabilitation |
| HS090 | Doriana Agop Forna, Mariana Ilie | CLASSIC IMPLANT -PROSTHETIC THERAPY VERSUS GUIDED IMPLANT -PROSTHETIC THERAPY | Romanian Journal of Oral Rehabilitation |
| HS091 | Tatiana Chisnoiu, Alexandru Cosmin Pantazi | CORRELATION BETWEEN METABOLIC CONTROL AND OXIDATIVE STRESS IN TYPE 1 DIABETES: MALONDIALDEHYDE AS A PROTECTIVE FACTOR | Romanian Journal of Oral Rehabilitation |
| HS092 | Adina Armencia, Mădălina Duceac (Covrig) | KNOWLEDGE REGARDING THE INDICATIONS, CONTRAINDICATIONS AND ADVANTAGES OF CBCT EXAMINATIONS AMONG DENTISTS AND SPECIALISTS | Romanian Journal of Oral Rehabilitation |
| HS093 | Irina Grădinaru, Vlad Dănilă | STUDY ON QUALITY OF LIFE CORRELATED WITH SENSE OF SAFETY, AND DENTAL ANXIETY | Romanian Journal of Oral Rehabilitation |
| HS094 | Kamel Earar, Ramona Diana Feier | EVALUATION OF THE MASTICATORY FUNCTION RESTITUTION IN THE EVENT OF PARTIAL TOOTHLESS WITH THE HELP OF FLEXIBLE MATERIALS | Romanian Journal of Oral Rehabilitation |
| HS095 | Ahmed BA | THE REMOVABLE PROSTHESIS AT THE BAMAKO UNIVERSITY DENTAL HOSPITAL-EPIDEMIOLOGICAL STUDY | Romanian Journal of Oral Rehabilitation |
| HS096 | Anca-Oana Dragomirescu, Elena-Claudia Coculescu | MORPHOLOGICAL CHARACTERISTICS OF MANDIBULAR SYMPHYSIS AND SAGITTAL INCLINATION OF LOWER INCISORS IN CLASS III MALOCCLUSION ACCORDING TO FACIAL DIVERGENCE PATTERN | Romanian Journal of Oral Rehabilitation |
| HS097 | Norina Forna, Doriana Agop-Forna | CLINICAL AND RADIOLOGICAL ASSESSMENT OF THE PERIODONTAL AND PERI-IMPLANT HEALTH STATUS IN PATIENTS TREATED BY IMPLANT-PROSTHETIC THERAPY | Romanian Journal of Oral Rehabilitation |
| HS098 | Mihai Săndulescu, Ion Alexandru Popovici | A CONDYLOGRAPHIC STUDY OF VARIATIONS OF THE BENNETT ANGLE AND ITS IMPORTANCE IN NATURAL TEETH- AND IMPLANT-SUPPORTED RESTORATIONS | Romanian Journal of Oral Rehabilitation |
| HS099 | Bratu Cristina, Buștiuc Steliana-Gabriela | CLINICAL ASPECTS OF THE ORAL MUCOSA IN PATIENTS WITH SLEEP-OCCURRING BRUXISM | Romanian Journal of Oral Rehabilitation |
| HS100 | Daniela Maria Pop | THE ADVANTAGES OF THE IMPLEMENTATION OF DIGITAL FLOW IN THE WORKING TIME MANAGEMENT OF DENTAL PROSTHESES RESTORATIONS | Romanian Journal of Oral Rehabilitation |
| HS101 | Mihai Săndulescu | PNEUMATIZATION PATTERNS IN THE TEMPORAL COMPONENT OF THE TEMPOROMANDIBULAR JOINT – A CONE-BEAM COMPUTED TOMOGRAPHY ORIGINAL STUDY AND REVIEW | Romanian Journal of Oral Rehabilitation |
| HS102 | Stefana Luca | PRINCIPLES OF FACE BURNS MANAGEMENT: PERSONAL EXPERIENCE | Romanian Journal of Oral Rehabilitation |
| HS103 | Alexandra Roi | EVALUATION OF ACCIDENTS AND COMPLICATIONS OF MAXILLARY THIRD MOLAR EXTRACTION | Romanian Journal of Oral Rehabilitation |
| HS104 | Ludmila Lozneanu | HIGHLIGHTING THE PHARYNGOCUTANEOUS FISTULA RISK FACTORS AFTER TOTAL LARYNGECTOMY | Romanian Journal of Oral Rehabilitation |
| HS105 | Ahmed Ba | NEED FOR PROSTHETIC TREATMENT IN COMMUNE I OF BAMAKO | Romanian Journal of Oral Rehabilitation |
| HS106 | Oana Raluca Temneanu, Letiția Doina Duceac | IMAGING PRINCIPLES IN DIAGNOSIS OF NASOPHARYNGEAL LESIONS USING COMPUTED-TOMOGRAPHY AND MAGNETIC RESONANCE IN PAEDIATRIC PATIENTS | Romanian Journal of Oral Rehabilitation |
| HS107 | Ionut Taraboanta, Galina Pancu | EVALUATION OF SMOKING EFFECTS ON SALIVARY PARAMETERS IN CARIOUS DISEASE | Romanian Journal of Oral Rehabilitation |
| HS108 | Maria Antonela Beldiman, Eugeniu Mihalas | CORRELATIONS BETWEEN VITAMIN D LEVEL AND THE PRESENCE OF LARGE CHRONIC PERIAPICAL LESIONS IN PATIENTS UNDERGOING COMPLEX ORAL REHABILITATION TREATMENT | Romanian Journal of Oral Rehabilitation |
| HS109 | Claudia Florina Bogdan-Andreescu, Oana Botoacă | THE EFFECT OF DIETARY COMPONENTS ON THE ORAL MICROBIOME COMPOSITION IN SCHOOL-AGED CHILDREN AND ADOLESCENTS | Romanian Journal of Oral Rehabilitation |
| HS110 | Marilena Bataiosu | ASSESSMENT OF PTX3 LEVELS IN GINGIVAL CREVICULAR FLUID AND GINGIVAL INFLAMMATION DURING TOOTH ERUPTION IN CHILDREN WITH TYPE 1 DIABETES VERSUS CHILDREN WITHOUT SYSTEMIC CONDITIONS | Romanian Journal of Oral Rehabilitation |
| HS111 | Monica Mihaela Scutariu, Andra Tărăboanță-Gamen | GENDER PROFILE OF HEALTHCARE-ASSOCIATED INFECTIONS IN ORAL AND MAXILLOFACIAL SURGERY CLINIC IAȘI (2011-2018) | Romanian Journal of Oral Rehabilitation |
| HS112 | Cristina-Crenguţa Albu, Claudia Florina Bogdan-Andreescu | COMPARISON OF ANTIMICROBIAL ACTIVITY OF TWO COMMERCIAL TOOTHPASTES | Romanian Journal of Oral Rehabilitation |
| HS113 | Gabriela Păduraru, Laura Otilia Boca | THE CONNECTION BETWEEN ORAL-GUT AXIS AND PEDIATRIC INFLAMMATORY BOWEL DISEASE | Romanian Journal of Oral Rehabilitation |
| HS114 | Măgirescu Alexandra | ANALYSIS OF PATIENT`S SATISFACTION AND REASONS FOR CLINICAL PRESENTATION REGARDING PROSTHODONTIC TREATMENTS | Romanian Journal of Oral Rehabilitation |
| HS115 | Diana-Andreea Ilinca, Alexandru Nemtoi | SURGICAL AND CBCT CORRELATION IN ACQUIRED CHOLESTEATOMA IN THE NORTH-EAST REGION OF ROMANIA | Romanian Journal of Oral Rehabilitation |
| HS116 | Sorana Maria Bucur, Ada Gabriela Delean | COMPARATIVE STUDY ON THE GUIDANCE OF ORTHODONTIC TREATMENTS THROUGH ARTIFICIAL INTELLIGENCE-BASED SOFTWARE AND CLASSIC SOFTWARE | Romanian Journal of Oral Rehabilitation |
| HS117 | Victor Vlad Costan | STUDY ON LIFE QUALITY OF PATIENTS DIAGNOSED WITH HEAD AND NECK CANCER | Romanian Journal of Oral Rehabilitation |
| HS118 | Geanina Totolici, Ana Magdalena Bratu | DETECTING OF CONJUNCTIVAL MICROANGIOPATHY IN CHILDREN AND TEENAGERS WITH TYPE I DIABETES-A NEW PROPOSED APPROACH | Romanian Journal of Oral Rehabilitation |
| HS119 | Marius Traian Leretter | NEW APPROACHES TO LASER TREATMENT FOR ORAL PERI-IMPLANT MUCOSITIS | Romanian Journal of Oral Rehabilitation |
| HS120 | Cristian Cojocaru | THE DETECTION OF SARS-COV-2 IN NASOPHARYNGEAL SAMPLES USING AN ANTIGENIC TEST | Romanian Journal of Oral Rehabilitation |
| HS121 | Cristina Claudia Tarniceriu | ANATOMO-MORPHOLOGICAL STUDY AND NEW ORIGINAL CLASSIFICATION OF CONCHA BULLOSA | Romanian Journal of Oral Rehabilitation |
| HS122 | Ana Maria Țâncu, Elena Claudia Coculescu | PREVALENCE AND ENDODONTIC MANAGEMENT OF SEPARATED INSTRUMENTS INSIDE THE ROOT CANAL | Romanian Journal of Oral Rehabilitation |
| HS123 | Ana Sîrghie | MORPHOMETRIC AND VOLUMETRIC CBCT ANALYSIS OF THE ORBITS IN PATIENTS FROM NORTH-EASTERN PART OF ROMANIA | Romanian Journal of Oral Rehabilitation |
| HS124 | Marius Valeriu Hînganu | CARDIOVASCULAR PATTERN IN HIP OSTEOPOROSIS ETIOPATHOGENY- A SILENT DENTAL RISK | Romanian Journal of Oral Rehabilitation |
| HS125 | Yuriy Melnychuk | CYTOLOGICAL ASSESSMENT OF HEALING ODONTOGENIC INFLAMMATORY PROCESSES WHEN APPLYING HYDROGELS | Romanian Journal of Oral Rehabilitation |
| HS126 | Sebastian Candrea, Irina Lupșe | IN VIVO ASSESSMENT OF CHARCOAL TOOTHPASTE EFFECT ON ENAMEL APPEARANCE - A NATURAL ALTERNATIVE FOR DENTAL AESTHETICS | Romanian Journal of Oral Rehabilitation |
| HS127 | Cristian Guțu, Elena Roxana Bogdan Goroftei | ANTIMICROBIAL RESISTANCE TO KLEBSIELLA PNEUMONIAE IN A NORTH EASTERN ROMANIAN HOSPITAL: a descriptive study | Romanian Journal of Oral Rehabilitation |
| HS128 | Mariana Păcurar, Ada Gabriela Delean | MONITORING AND INFORMATION FOR CARIES RISK CONTROL- BETWEEN ADVANTAGES AND LIMITATIONS | Romanian Journal of Oral Rehabilitation |
| HS129 | Cristina-Crenguța Albu, Mihaela Tănase | CARIES EXPERIENCE IN CHILDREN WITH SEVERE EARLY CHILDHOOD CARIES | Romanian Journal of Oral Rehabilitation |
| HS130 | Zsuzsanna Bardocz-Veres | STUDY OF MORPHOLOGICAL VARIATIONS OF THE UPPER FIRST PERMANENT MOLAR IN ASSOCIATION WITH NONSYNDROMIC TOOTH AGENESIS | Romanian Journal of Oral Rehabilitation |
| HS131 | Marius Valeriu Hînganu, Monica Mihaela Scutariu | MORPHOLOGICAL AND VASCULAR CHARACTERISTICS OF THE SUPERFICIAL MUSCLO-APONEUROTIC SYSTEM OF THE ORAL REGION | Romanian Journal of Oral Rehabilitation |
| HS132 | Cristina Maria Mihai, Tatiana Chisnoiu, Ancuta Lupu | SERUM LACTATE – PREDICTIVE FACTOR IN SEPTIC SHOCK IN INFANTS AND CHILDREN | Romanian Journal of Oral Rehabilitation |
| HS133 | Ovidiu Stamatin, Veronica Serban Pintiliciuc | HEARING LOSS (HL) PATIENTS’ TREATMENT APPROACHES, MENTAL HEALTH ISSUES, AND SOCIAL ORAL PATHOLOGIES - PART 2 - TREATMENT PLAN | Romanian Journal of Oral Rehabilitation |
| HS134 | Dorin Ioan Cocoș, Radu Mircea Sireteanu Cucui | DENTITION CHANGES IN GENETIC DISEASES | Romanian Journal of Oral Rehabilitation |
| HS135 | Magda Ecaterina Antohe | CORRELATIONS BETWEEN THE DENTAL MEDICINE STUDENTS’ OPINIONS ABOUT ONLINE LEARNING AND THEIR MOTIVATIONAL PERSISTENCE SKILLS | Romanian Journal of Oral Rehabilitation |
| HS136 | Anca-Cristina Perpelea, Ruxandra Sfeatcu | THE TWOFOLD PERSPECTIVE OF SCHOOLCHILDREN’S ORAL HEALTH | Romanian Journal of Oral Rehabilitation |
| HS137 | Alexandra Mihaela Stoica | THE BUFFER CAPACITY OF NIGHT SALIVA SECRETION ON STREPTOCOCCUS MUTANS DYNAMICS IN YOUNG CHILDREN: A COMPREHENSIVE STUDY | Romanian Journal of Oral Rehabilitation |
| HS138 | Laura Elisabeta Checherita, Magda Calina Barlean | INTERDISCIPLINARITY APPROACHES IN CONTEXT OF COMPLEX INTRAORAL REHABILITATION TREATMENT AT YOUNG PATIENTS WITH SOCIAL ANXIETY - PART 2 – DIAGNOSTIC AND TREATMENT | Romanian Journal of Oral Rehabilitation |
| HS139 | Gabriel Valeriu Popa | INSIGHT INTO HOW AMBIENT TEMPERATURE AFFECTS ORAL HEALTH AND SALIVARY OXIDATIVE STRESS IN CHILD ATHLETES DURING COMPETITIVE SPORTS | Romanian Journal of Oral Rehabilitation |
| HS140 | Cernei Eduard Radu | RETROSPECTIVE STUDY ON THE FREQUENCY OF ORO-MAXILLOFACIAL TRAUMAS AT ”SF. SPIRIDON” HOSPITAL IN IAȘI (2021-2022) | Romanian Journal of Oral Rehabilitation |
| HS141 | Cristian Constantin Budacu | GOLDEN QUADRILATERAL OF THE MAXILLARY BONE | Romanian Journal of Oral Rehabilitation |
| HS142 | Daniela Păcurar | CHRONIC VIRAL HEPATITIS IN CHILDREN - SOCIO-ECONOMIC IMPLICATIONS IN ROMANIA | Romanian Journal of Oral Rehabilitation |
| HS143 | Maria-Alexandra Martu | CLINICAL STUDY ON THE ASSESSMENT OF LOCAL STATUS IN PATIENTS WITH PERIODONTAL DISEASE AND DEPRESSION | Romanian Journal of Oral Rehabilitation |
| HS144 | Maria-Alexandra Martu, Irina-Georgeta Sufaru | INVESTIGATING THE IMPACT OF SYSTEMIC ALENDRONATE ON PERIODONTAL PARAMETERS IN OSTEOPOROSIS AND PERIODONTITIS PATIENTS. AN INTERVENTIONAL AND PROSPECTIVE STUDY | Romanian Journal of Oral Rehabilitation |
| HS145 | Sînziana-Călina Silişteanu | CHANGES IN NUTRITIONAL STATUS AND THEIR INFLUENCE ON BONE MASS | Romanian Journal of Oral Rehabilitation |
| HS146 | Carmen Liliana Defta, Oana Botoacǎ | APPLICATION OF ARTIFICIAL INTELLIGENCE IN DENTAL CARIES PREDICTION RELATED TO DIET AND ORAL HYGIENE | Romanian Journal of Oral Rehabilitation |
| HS147 | Otilia Boisteanu, Daniela Sulea | COMPARATIVE STUDY BETWEEN POSTOPERATIVE PARATHYROID DEFICIENCY HYPOCALCEMIA IN THOSE WITH REIMPLANTATION VERSUS THOSE WITHOUT REIMPLANTATION | Romanian Journal of Oral Rehabilitation |
| HS148 | Laura Elisabeta Checheriţă, Veronica Serban Pintiliciuc, Liana Aminov | TEMPOROMANDIBULAR MANIFESTATION AN IMPORTANT ISSUE IN REHABILITATION TREATMENT PLAN AT PATIENTS WITH OBESITY PATHOLOGY AND STRESS DISORDERS.- diagnostic and treatment -part 2- | Romanian Journal of Oral Rehabilitation |
| HS149 | Mona Ionas | EVALUATION OF CURRENT KNOWLEDGE LEVEL ON DATA SECURITY OF PEOPLE WORKING IN A MEDICAL ENVIRONMENT | Romanian Journal of Oral Rehabilitation |
| HS150 | Doriana Agop Forna, Alketa Qafmolla | HISTO-MORPHOLOGICAL STUDY OF FUNCTIONAL OVERLOAD (CLINICAL EXPERIMENTAL STUDY) | Romanian Journal of Oral Rehabilitation |
| HS151 | Maria Alexandra Martu, Elena-Odette Luca | STUDY OF THE EFFECTS OF SELECTIVE OCCLUSAL ADJUSTMENT THERAPY ON THE BACTERIOLOGICAL PROFILE IN OCCLUSAL TRAUMA | Romanian Journal of Oral Rehabilitation |
| HS152 | Anca Sava, Maria-Antonela Beldiman, Oana Țănculescu | SCREENING OF TEMPOROMANDIBULAR DISORDERS | Romanian Journal of Oral Rehabilitation |
| HS153 | Zsuzsanna Bardocz-Veres | EFFECT OF DIFFERENT CLEANING PROCEDURES ON THE SURFACE ROUGHNESS OF VACUUM-FORMED RETAINER MATERIALS | Romanian Journal of Oral Rehabilitation |
| HS154 | Andrei Georgescu, Antonia Moldovanu | NON-INTERVENTION VERSUS REPAIR/REPLACEMENT DECISIONS IN POSTERIOR COMPOSITE RESTORATIONS AGED 3-5 YEARS: A RETROSPECTIVE STUDY | Romanian Journal of Oral Rehabilitation |
| HS155 | Emanuela Lidia Crăciunescu | NUTRITIONAL PROFILING AND SENSORY EVALUATION OF NOVEL HOT CHOCOLATE RECIPES: A COMPARATIVE STUDY WITH A COMMERCIAL BRAND | Romanian Journal of Oral Rehabilitation |
| HS156 | Mihaela-Roxana Boțilă, Oana Andreea Diaconu | RETROSPECTIVE STUDY ON THE EVOLUTION OF TEETH WITH ENDODONTIC TREATMENT IN A GROUP OF PATIENTS FROM CRAIOVA – ROMANIA | Romanian Journal of Oral Rehabilitation |
| HS157 | Florin Iacob | EFFECTS OF CANNABIDIOL EXTRACT FROM ELECTRONIC CIGARETTES ON ORAL HEALTH | Romanian Journal of Oral Rehabilitation |
| HS158 | Maria-Alexandra Martu, Vasilica Toma | STUDY ON THE EVALUATION OF PERIODONTAL CLINICAL PARAMETERS IN PEDIATRIC PATIENTS POST-SARS-COV-2 INFECTION | Romanian Journal of Oral Rehabilitation |
| HS159 | Mihaela Ionescu, Diana Elena Vlăduțu | THE NUMBER OF LOST TEETH - A POTENTIAL PREDICTIVE MARKER FOR THE CARDIOVASCULAR DISEASES IN A SAMPLE OF HOSPITALIZED ADULTS | Romanian Journal of Oral Rehabilitation |
| HS160 | Cristina Popa, Elena-Raluca Baciu | POTENTIALLY MALIGNANT LESIONS IN THE ORAL CAVITY: A RETROSPECTIVE ANALYSIS | Romanian Journal of Oral Rehabilitation |
| HS161 | Octavian Cătălin Ciobotaru, Iulian Cătălin Bratu | OCULAR REFRACTIVE STATUS IN CHILDREN AND ADOLESCENTS WITH TYPE 1 DIABETES | Romanian Journal of Oral Rehabilitation |
| HS162 | Cristina Dascălu | EVALUATION OF PERIAPICAL AREA IN ROOT-FILLED TEETH WITH PERIAPICAL LESIONS: A CBCT STUDY | Romanian Journal of Oral Rehabilitation |
| HS163 | Juliánna Szakács, Oana Raluca Antonescu | CHANGES INDUCED BY PHYSICAL EXERCISE AND THERAPEUTIC SWIMMING ON THE HEALTH STATUS OF MENOPAUSAL AND POSTMENOPAUSAL WOMEN WITH OSTEOPOROSIS | Romanian Journal of Oral Rehabilitation |
| HS164 | Elena Claudia Coculescu, Anca Oana Dragomirescu | ENDODONTIC ANATOMY OF SECOND MAXILLARY PREMOLARS AND THEIR RELATION TO MAXILLARY SINUS AS INDICATED BY CONE-BEAM COMPUTED TOMOGRAPHY | Romanian Journal of Oral Rehabilitation |
| HS165 | Cristina Dascălu | CBCT-PAI SCORES IN ROOT-FILLED TEETH WITH ENDODONTIC TREATMENT FAILURE: A RETROSPECTIVE STUDY | Romanian Journal of Oral Rehabilitation |
| HS166 | Elena Cristina Andrei | CLINICAL AND MORPHOLOGICAL ASPECTS OF GINGIVAL OVERGROWTH INDUCED BY FIXED ORTHODONTIC THERAPY | Romanian Journal of Oral Rehabilitation |
| HS167 | Dorin Ioan Cocoș, Alexandra-Camelia Pogăcian-Maier | RADIOGRAPHIC DETERMINATION OF THE DIFFICULTY IN EXTRACTION OF THE THIRD IMPACTED MANDIBULAR MOLARS | Romanian Journal of Oral Rehabilitation |
| HS168 | A. Ivan (first author) | OVEREXPRESSION OF IGF1 PROGNOSTIC FACTOR IN CHILDHOOD OSTEOSARCOMAS | Romanian Journal of Oral Rehabilitation |
| HS169 | Magda Ecaterina Antohe | THE EFFICIENCY OF QUALITATIVE DATA CLUSTERING IN MEDICAL AND SOCIO-ECONOMIC SURVEYS – COMPARATIVE STUDY | Romanian Journal of Oral Rehabilitation |
| HS170 | Gabriela Geletu, Roxana Vasluianu | RISK PERCEPTION OF WORK-RELATED INFECTIONS AMONG DENTAL STUDENTS IN IASI, ROMANIA | Romanian Journal of Oral Rehabilitation |
| HS171 | Marina-Cristina Giurgiu, Anca Silvia Dumitriu | ROMANIAN CHILDREN'S PERCEPTION OF DENTAL CAVITIES AND GINGIVAL BLEEDING IN CORRELATION WITH PERSONAL ORAL BEHAVIOURS – A CROSS SECTIONAL STUDY | Romanian Journal of Oral Rehabilitation |
| HS172 | Anca Dragomirescu, Elena Zabrac | ROOT PERFORATIONS IN ENDODONTICALLY-TREATED POSTERIOR TEETH: A CONE-BEAM COMPUTED TOMOGRAPHY STUDY | Romanian Journal of Oral Rehabilitation |
| HS173 | Claudiu Topoliceanu, Antonia Moldovanu | EVALUATION OF THE REMINERALIZATION CAPACITY OF SALIVA (IMK) IN PATIENTS WITH SJÖGREN SYNDROME | Romanian Journal of Oral Rehabilitation |
| HS174 | Victor Vlad Costan | STUDY ON LIFE QUALITY IN SURGICALLY TREATED HEAD & NECK CANCERS PATIENTS: A FOLLOW-UP STUDY | Romanian Journal of Oral Rehabilitation |
| HS175 | Andreea Simona Pop | DO THE UNTREATED SURFACES OF ORTHODONTIC MINI-IMPLANTS HAVE OSSEOINTEGRATION? | Romanian Journal of Oral Rehabilitation |
| HS176 | Alexandra Elena Done, Cristina Teodora Preoteasa | DENTAL ANXIETY AND DENTAL ATTENDANCE FREQUENCY OF DENTAL STUDENTS VERSUS STUDENTS MAJORING IN OTHER FIELDS | Romanian Journal of Oral Rehabilitation |
| HS177 | Ahmed Ba | THE AESTHETIC NEEDS OF PATIENTS IN THE DEPARTMENT OF REMOVABLE PROSTHESIS FROM THE CHU-CNOS OF BAMAKO | Romanian Journal of Oral Rehabilitation |
| HS178 | Paul Lucian Nedelea | QUALITATIVE STUDY ON THE RESILIENCE OF MEDICAL STAFF IN EMERGENCY UNITS IN THE COVID-19 PANDEMIC | Romanian Journal of Oral Rehabilitation |
| HS179 | Kamel Earar, Norina Consuela Forna | ENDOCROWN AS A SOLUTION FOR PROSTHETIC RESTORATION OF THE LATERAL TEETH WITH ENDODONTIC TREATMENT | Romanian Journal of Oral Rehabilitation |
| HS180 | A. Ivan (first author) | STUDY ON THE OVEREXPRESSION OF IGF1 AND CRIP 1 IN CHILDREN OSTEOSARCOMAS | Romanian Journal of Oral Rehabilitation |
| HS181 | Codruta Victoria Tigmeanu | KNOWLEDGE, ATTITUDES AND PRACTICES RELATED TO PREVENT ORAL COMPLICATIONS DURING CHEMOTHERAPY AMONG PATIENTS ATTENDING AN ONCOLOGY CLINIC IN ROMANIA | Romanian Journal of Oral Rehabilitation |
| HS182 | Horia Octavian Manolea | APPLICATIONS OF DIGITAL TECHNOLOGY IN DATA TRANSFER FROM THE DENTAL OFFICE TO THE DENTAL LAB | Romanian Journal of Oral Rehabilitation |
| HS183 | Octavian Marius Dincă, George Cristian Vlădan | THE IMPACT OF PURINE DERIVATIVES THERAPY FOR PATIENTS WITH MEDICATION-RELATED OSTEONECROSIS OF THE JAW: PRELIMINARY RESULTS FROM A PILOT STUDY | Romanian Journal of Oral Rehabilitation |
| HS184 | Cristina Dascălu, Adina Constantin | EVALUATION OF ROOT-FILLED TEETH BY PERIAPICAL AND ENDODONTIC STATUS SCALE (PESS): A CBCT STUDY | Romanian Journal of Oral Rehabilitation |
| HS185 | Anca Dragomirescu, Elena Coculescu | APICAL PERIODONTITIS IN MAXILLARY MOLARS WITH MISSED SECOND MESIO-BUCCAL ROOT CANAL: A CBCT STUDY | Romanian Journal of Oral Rehabilitation |
| HS186 | Costuleanu Marcel, Agop-Forna Doriana | REHABILITATION OF ALVEOLAR BONE THROUGH GUIDED TISSUE REGENERATION WITH AUTOGENOUS BONE AND XENOGRAFTS: CBCT STUDY | Romanian Journal of Oral Rehabilitation |
| HS187 | Cristina Bartok-Nicolae, Steliana-Gabriela Buștiuc | INFLUENCE OF TOBACCO ON COLOR PARAMETERS OF CANINES IN ADOLESCENTS | Romanian Journal of Oral Rehabilitation |
| HS188 | Mihaela Pantea | RELIABILITY OF FRANKFURT MANDIBULAR ANGLE MEASUREMENT ON PANORAMIC RADIOGRAPHIES CONSIDERING SOME PATIENT POSITION VARIATIONS | Romanian Journal of Oral Rehabilitation |
| HS189 | Livia Bobu, Petruța Siminiuc | ASSESSMENT OF ORAL HEALTH BEHAVIOR IN ADOLESCENTS WITH VARIOUS SOCIAL BACKGROUNDS | Romanian Journal of Oral Rehabilitation |
| HS190 | Nema Khedr Sayed | THE USE OF TRIPHASIC HYALURONAN-CHITOSAN-STRONTIUM CHLORIDE MEMBRANE IN TREATING INTRABONY DEFECT “RANDOMIZED CLINICAL TRIAL” | Romanian Journal of Oral Rehabilitation |
| HS191 | A. Ba (first author) | PREPROSTHETIC PREPARATIONS EDENTULUS AT BAMAKO CHU-CNOS | Romanian Journal of Oral Rehabilitation |
| HS192 | Gabriela Stoleriu, Oana Monica Duca | THE EARLY STAGE ADENOCARCINOMA OF THE GALLBLADDER INCIDENTALLY DIAGNOSED | Romanian Journal of Oral Rehabilitation |
| HS193 | Anca-Oana Dragomirescu, Cristina-Crenguţa Albu | GENETIC POLYMORPHISMS OF INTERLEUKINS IL-1A, IL-1B, AND IL-1RN IN PATIENTS WITH PERIODONTAL DISEASE AND DENTO-MAXILLARY ANOMALIES | Romanian Journal of Oral Rehabilitation |
| HS194 | Maria Chisnoiu | CLINICAL ASPECTS OF RECORDING OCCLUSAL CONTACTS USING CONVENTIONAL METHODS AND INTRA-ORAL SCANNING | Romanian Journal of Oral Rehabilitation |
| HS195 | Gabriela Ghiga | THE RELATIONSHIP BETWEEN CHILDHOOD OBESITY AND DENTAL HEALTH. AN OBSERVATIONAL STUDY | Romanian Journal of Oral Rehabilitation |
| HS196 | Ancuta Lupu, Nicuta Manolache | PARTICULAR ASPECTS OF THE ORAL MUCOSA AS PREDICTIVE FACTORS FOR THE EVOLUTION OF PSORIASIS | Romanian Journal of Oral Rehabilitation |
| HS197 | Ştefan-Dimitrie Albu, Matei Georgian Brăila | THE SPECTRUM OF BIOCHEMICAL, HORMONAL, AND METABOLIC CHANGES - A DETERMINING FACTOR IN THE MANAGEMENT OF PSYCHOSOCIAL AND MEDICAL IMPLICATIONS OF POLYCYSTIC OVARY SYNDROME | Romanian Journal of Oral Rehabilitation |
| HS198 | Ancuta Lupu, Cristina Stanescu | STUDY ON THE IMPACT OF ORAL EROSIONS ON THE QUALITY OF LIFE OF PATIENTS WITH LICHEN PLANUS | Romanian Journal of Oral Rehabilitation |
| HS199 | Laurențiu Pascu | THE USE OF CENTRIC RELATION IN COMPLEX ORAL REHABILITATIONS. A PERSONALIZED SURVEY-BASED STUDY | Romanian Journal of Oral Rehabilitation |
| HS200 | A. Ba (first author) | THE AESTHETIC NEEDS OF PATIENTS IN THE DEPARTMENT OF REMOVABLE PROSTHESIS FROM THE CHU-CNOS OF BAMAKO | Romanian Journal of Oral Rehabilitation |
| HS201 | Agop-Forna Doriana | PRE-PROSTHETIC AND PRE-IMPLANT THERAPEUTIC MANAGEMENT OF EDENTULOUS PATIENTS TO IMPROVE BIOMECHANICAL STABILITY OF FUTURE HYBRID PROSTHESES | Romanian Journal of Oral Rehabilitation |
| HS202 | Claudia Florida Costea | THE DIAMETERS OF THE CIRCLE OF WILLIS ARTERIES IN A ROMANIAN POPULATION AND THEIR SIGNIFICANCE IN CEREBROVASCULAR DISEASES | Romanian Journal of Oral Rehabilitation |
| HS203 | Anamaria Bechir | COMPARATIVE RESULTS IN GUMMY SMILE TREATMENT WITH TWO DIFFERENT TYPES OF THERAPY | Romanian Journal of Oral Rehabilitation |
| HS204 | Gabriela Calin | MULTIDISCIPLINARY APPROACH OF CAUSAL IMBALANCES OCLUZAL-POSTURE REHABILITATION | Romanian Journal of Oral Rehabilitation |
| HS205 | Loredana Liliana Hurjui, Anca Haisan | THE CLINICAL EXPRESSION OF THROMBOCYTOPENIA AND THE HEALTH IMPACT OF THE ORAL CAVITY | Romanian Journal of Oral Rehabilitation |
| HS206 | Cosmin Ifrim | DIGITAL ASSESSMENT OF DENTAL OCCLUSION | Romanian Journal of Oral Rehabilitation |
| HS207 | Ana-Petra Lazăr | SOCIO-BEHAVIORAL DETERMINANTS FOR SEVERE EARLY CHILDHOOD CARIES (S-ECC) | Romanian Journal of Oral Rehabilitation |
| HS208 | George Cristian Vlădan | THE IMPACT OF PURINE DERIVATIVES THERAPY FOR PATIENTS WITH MEDICATION-RELATED OSTEONECROSIS OF THE JAW: PRELIMINARY RESULTS FROM A PILOT STUDY – THE SEQUEL | Romanian Journal of Oral Rehabilitation |
| HS209 | Stefania Dinu | COMPARATIVE ANALYSIS OF INTERMAXILLARY REGISTRATION: ANTERIOR JIG METHOD VS. CLASSIC APPROACH IN ANTERIOR REHABILITATION WITH LATERAL OCCLUSAL CONTACT PRESERVATION | Romanian Journal of Oral Rehabilitation |
| HS210 | Emanuela Lidia Petrescu | ETHICAL AND LEGAL CONSIDERATIONS IN RESEARCH STUDIES INVOLVING HUMAN EMBRYOS | Romanian Journal of Oral Rehabilitation |
| HS211 | Irina-Georgeta Șufaru, Fabian Cezar Lupu | COMPARATIVE STUDY OF THE EFFECTS OF A-PRF VERSUS GTR IN THE TREATMENT OF COMBINED ENDO PERIODONTAL LESIONS | Romanian Journal of Oral Rehabilitation |
| HS212 | Mihaela Salceanu, Danisia Haba | DIAGNOSIS OF THE VARIATION IN THE SIZE OF PERIAPICAL LESIONS BY USING CT BEFORE AND AFTER RADIOTHERAPY IN PATIENTS WITH ENT CANCER | Romanian Journal of Oral Rehabilitation |
| HS213 | Anca Melian, Danisia Haba | THE SIDE EFFECTS OF RADIOTHERAPY IN THE PATIENT WITH ENT CANCER | Romanian Journal of Oral Rehabilitation |
| HS214 | Janice Townsend | OHIO SCHOOL NURSES' PERCEPTIONS OF SCHOOL ABSENCES FOR DENTAL CARE | Journal of Dentistry for Children |
| HS215 | Robert S Jones | RETROSPECTIVE EVALUATION OF MODERATE SEDATION VISITS THAT USED ORAL MEPERIDINE AND HYDROXYZINE WITH ORAL OR INTRANASAL MIDAZOLAM | Journal of Dentistry for Children |
| HS216 | John M. Burnheimer | PREVALENCE OF SLEEP-DISORDERED BREATHING IN CHILDREN AGES SEVEN TO 10 YEARS: A COMPARATIVE STUDY BEFORE AND AFTER COVID-19 LOCKDOWN | Journal of Dentistry for Children |
| HS217 | Akanksha Juneja | THE IMPACT OF PARENTING STYLES ON PEDIATRIC DENTAL BEHAVIOR AND ANXIETY IN THE DENTAL OPERATORY | Journal of Dentistry for Children |
| HS218 | Erenay Alpayçetin | PARENTS’ KNOWLEDGE AND ATTITUDES REGARDING TEETHING SIGNS AND SYMPTOMS | Journal of Dentistry for Children |
| HS219 | Dean Phan | SILVER DIAMMINE FLUORIDE USAGE IN GENERAL DENTISTRY OFFICES IN LOUISIANA | Journal of Dentistry for Children |
| HS220 | Bianca Spuri Tavares | LATE ERUPTION OF MANDIBULAR CENTRAL INCISOR IN SMALL FOR-GESTATIONAL-AGE INFANTS: A COHORT STUDY | Journal of Dentistry for Children |
| HS221 | Raymond Lee | The Effect of Temperament on Outcomes of Opioid and Non-Opioid Pediatric Dental Sedation | Journal of Dentistry for Children |
| HS222 | Paulo Antônio Martins-Júnior | Early Childhood Caries and Its Consequences Impact Sleep in Preschool Children | Journal of Dentistry for Children |
| HS223 | Rekhalakshmi Kamatham | Effectiveness of Animal-Assisted Activity on Pain Perception and Anxiety of Children Undergoing Intraoral Local Anesthetic Administration | Journal of Dentistry for Children |
| HS224 | Manuel Restrepo | Acceptance of Behavior Guidance Techniques in Pediatric Dentistry Between American and Colombian Parents | Journal of Dentistry for Children |
| HS225 | Kimberly J. Hammersmith | COVID-19 Pandemic-Related Behavioral and Attitudinal Changes in Caregivers | Journal of Dentistry for Children |
| HS226 | Janice A. Townsend | Filled Opioid Prescriptions Following Pediatric Dental Procedures Among Medicaid-Insured Children in Ohio | Journal of Dentistry for Children |
| HS227 | Morankar Rahul | Efficacy of American Academy of Pediatric Dentistry Best Practice Recommendations for Diagnosis and Management of Deep Caries in Primary Teeth | Journal of Dentistry for Children |
| HS228 | Keri Discepolo | Caregivers’ Perceptions of Dental Therapists | Journal of Dentistry for Children |
| HS229 | Halima Abukabbos | In-Office Pediatric Emergency Dental Visits During and After the COVID Pandemic | Journal of Dentistry for Children |
